# MiR-708 promotes steroid-induced osteonecrosis of femoral head, suppresses osteogenic differentiation by targeting SMAD3

**DOI:** 10.1038/srep22599

**Published:** 2016-03-02

**Authors:** Cheng Hao, Shuhua Yang, Weihua Xu, Jacson K. Shen, Shunan Ye, Xianzhe Liu, Zhe Dong, Baojun Xiao, Yong Feng

**Affiliations:** 1 Orthopedic Hospital, Union Hospital, Tongji Medical College, Huazhong University of Science and Technology, Wuhan, Hubei, PR China; 2 Sarcoma Biology Laboratory, Department of Orthopaedic Surgery, Massachusetts General Hospital and Harvard Medical School, 55 Fruit Street, Jackson 1115, Boston, Massachusetts 02114.

## Abstract

Steroid-induced osteonecrosis of femoral head (ONFH) is a serious complication of glucocorticoid (GC) use. We investigated the differential expression of miRs in the mesenchymal stem cells (MSCs) of patients with ONFH, and aimed to explain the relationship between GC use and the development of MSC dysfunction in ONFH. Cells were collected from bone marrow of patients with ONFH. Samples were assigned to either GCs Group or Control Group at 1:1 matched with control. We then used miRNA microarray analysis and real-time PCR to identify the differentially expressed miRs. We also induced normal MSCs with GCs to verify the differential expression above. Subsequently, we selected some of the miRs for further studies, including miRNA target and pathway prediction, and functional analysis. We discovered that miR-708 was upregulated in ONFH patients and GC-treated MSCs. SMAD3 was identified as a direct target gene of miR-708, and functional analysis demonstrated that miR-708 could markedly suppress osteogenic differentiation and adipogenesis differentiation of MSCs. Inhibition of miR-708 rescued the suppressive effect of GC on osteonecrosis. Therefore, we determined that GC use resulted in overexpression of miR-708 in MSCs, and thus, targeting miR-708 may serve as a novel therapeutic biomarker for the prevention and treatment of ONFH.

Osteonecrosis of femoral head (ONFH) is a progressive disease with bone marrow and osteocyte death resulting in collapse of the femoral head. Intensification of therapy with glucocorticoids are frequently used to treat a wide range of autoimmune and inflammatory disorders^1^,^2^. However, one of the most common therapy-related and dose-limiting toxicities of these therapies is glucocorticoid-induced osteonecrosis^3^. The majority of symptomatic cases of osteonecrosis occur within the first two years of treatment. Osteonecrosis can result in debilitation and adversely affect quality of life, often requiring surgical intervention. So far, there are no effective preventive measures for glucocorticoid-induced ONFH.

Multipotent mesenchymal stem cells (MSCs) are a population of stem cells that have the potential to differentiate and develop into multiple tissues^4^,^5^,^6^. MSCs derived from bone marrow, which are non-hemopoietic (CD34-), remain ideal candidates for different cellular therapies for human orthopedic disorders^7^,^8^,^9^. It has been suggested that the development of some diseases are closely related to these cells, as decreased MSC activity in the bone marrow is related to non-traumatic ONFH^10^. Furthermore, dysfunctional MSCs from GC-associated ONFH showed reduced proliferation ability, elevated reactive oxygen species levels, and depressed mitochondrial membrane potential^11^. Moreover, glucocorticoid suppresses bone formation through their effects on MSCs^2^. On the other hand, activation of dexamethasone’s (Dex) canonical signaling pathway is necessary for inducing MSC adipogenic differentiation^12^. Thus, the identification of factors that regulate the osteogenic and adipogenic differentiation of MSCs holds potential for identifying novel targets to prevent glucocorticoid-induced ONFH.

MicroRNAs (miR) are a large family of small non-coding (17–25 nucleotides) single-stranded endogenous RNAs that have been identified as regulators of diverse biological processes, including cell proliferation, apoptosis, differentiation, and cell cycle progression. MiRs regulate gene expression by binding to the 3′ untranslated regions (3′-UTRs) of their target mRNAs via either promoting degradation of target mRNAs or inhibiting their translation^13^,^14^. Bioinformatic studies have suggested that miRs may regulate one-third of the transcriptome, suggesting the essential role of miRs in regulating gene expression^15^. Increasing evidence has demonstrated that miRs have critical functions in regulating MSC differentiation and other cellular properties, such as proliferation, survival, and migration^16^. Recently, a growing body of results has suggested that miRs have important roles in GC-associated pathophysiology^2^,^17^,^18^. However, the role of miRs in MSCs mediated by GCs-related ONFH is still unclear.

In our study, we applied miR microarray profiling to screen differential expression of miRs in GC-associated ONFH. We then identified miR-708 to be highly expressed in GC-ONFH, and used GCs to treat normal MSCs in gradient concentrations *in vitro* to verify high miR-708 expression. SMAD3, a SMAD family member, is a signal transducer and transcriptional modulator that mediates multiple signaling pathways, which was identified as a direct target of miR-708. Importantly, a miR-708 inhibitor rescued the GC suppression of MSC dysfunction. Our findings suggest that miR-708 may serve as a novel therapeutic target for the prevention and treatment of osteonecrosis and other bone metabolism-related diseases.

## Results

### The osteogenic differentiation capacity of GC-MSCs was subdued and the adipogenesis differentiation capacity was enhanced when compared with normal MSCs

**Figure 1:**
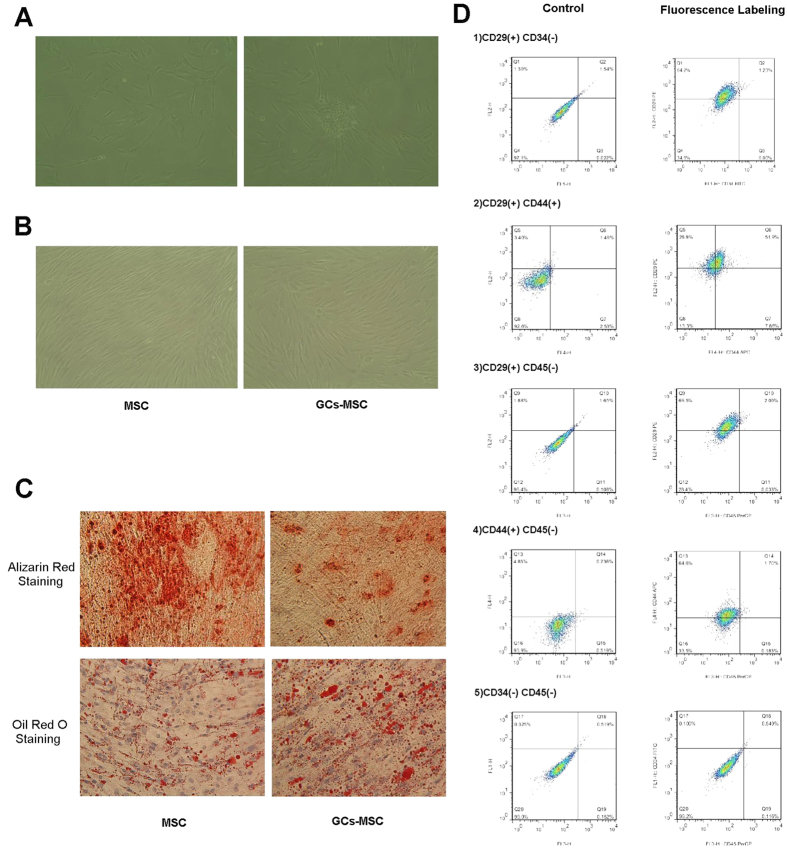
The osteogenic differentiation capacity of GCs-MSC was subdued and the adipogenesis differentiation capacity was enhanced when compared with normal MSCs. Images (100×) taken by inverted microscope showed MSCs in primary culture on the 7th day (**A**) and 12th day (**B**). GC-MSCs showed slower cell reproduction and some morphological variation compared with normal MSCs. When MSCs and GC-MSCs reached 80%–90% confluence, they were treated with osteoblast differentiation medium and adipogenic differentiation medium. Alizarin red S staining and Oil Red O staining was performed at 14 days (**C**), which show the change of coloration in GC-MSCs after staining. The double channel flow cytometry (**D**) indicated that our cells were human MSCs. Images showing negative control without fluorescence antibodies are in the left column. The displacement of splashes on the CD29-PE and CD44-APC channel are obvious.

After 5–7 days of primary culture, the number of erythrocytes and other suspension cells tended to decrease, and each of the colonies contained hundreds of MSCs, which were spindle shaped and flat (Fig. 1A). After 10–12 days of culture the cells proliferated rapidly and reached a confluence greater than 80% (Fig. 1B), which looked like shoal of fish. GC-MSCs showed slower cell reproduction and some morphological variation when compared with normal MSCs. The osteogenic and adipogenesis induction of MSCs and GC-MSCs were respectively stained with alizarin red S and oil red O (Fig. 1C), and the change of coloration in GC-MSCs after staining was distinct, which positively proved the differentiation capacity of our cells. At the same time, the results showed that the osteogenic differentiation capacity of GC-MSCs was subdued and that the adipogenesis differentiation capacity was enhanced when compared with normal MSCs.

As we know that MSCs are difficult to identify by simple markers, we characterized four phenotypes known to be associated with human MSCs by flow cytometry analysis of expressed surface antigens that differ from hemocytes and other monocytes. Three samples randomly chosen from the patients above were tested. The results showed that MSCs were uniformly positive for CD29 and CD44 and negative for hematopoietic lineage markers, including CD34 and CD45 (Fig. 1D)^19^. These results indicated that the culture cells were human MSCs.

### Microarray analysis revealed differential expression profiling of miRs between MSCs of GCs Group and Control Group

**Figure 2:**
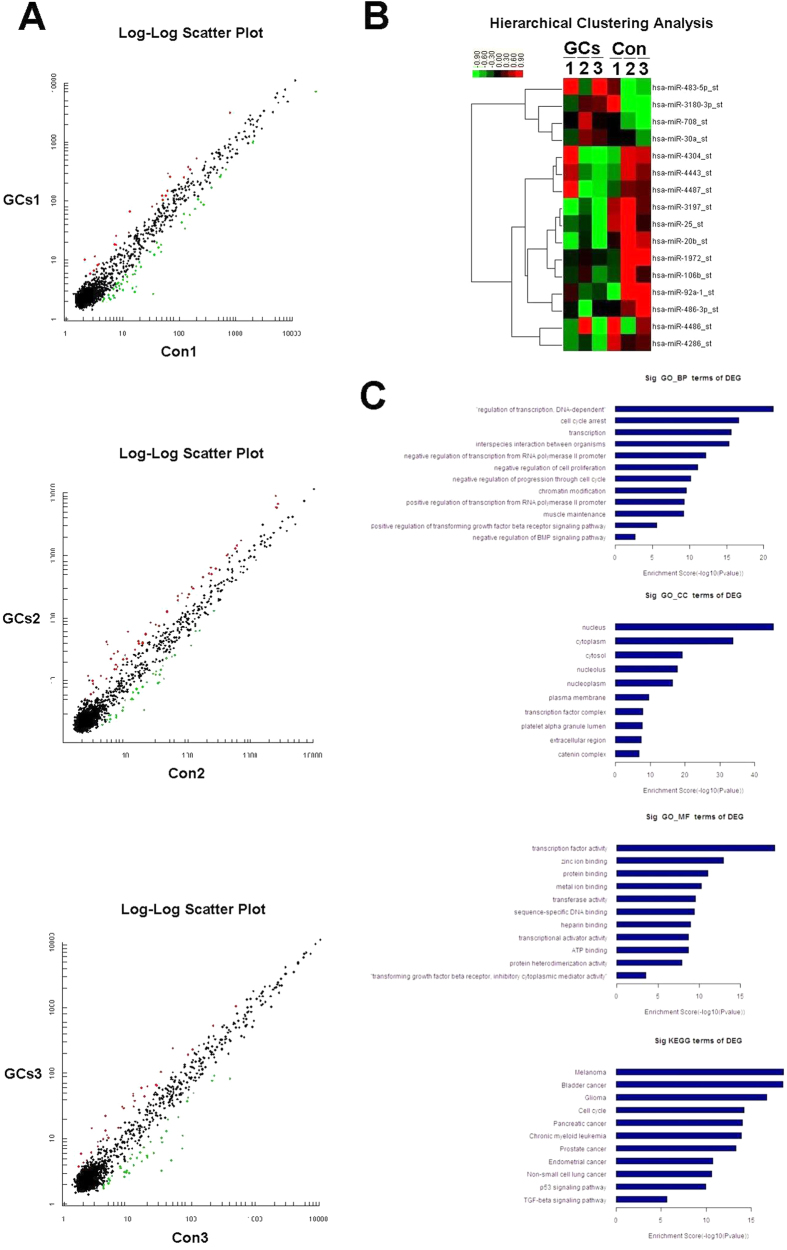
Microarray analysis revealed differential expression profiling of miRs between MSCs of GCs Group and Control Group. By microarray and subsequent related analysis, select miRs were chosen for the following studies. Three Log-Log Scatter Plots (**A**) show the comparison of each pair in miRs microarray analysis. Splashes with red color stand for those overexpressed miRs and green stand for those underexpressed miRs between each pair, which we are shown in Supplementary Tables 3 to 5. The hierarchical clustering (**B**) in the lower right corner show the differentially expressed miRs between GCs Groups and Control Groups, which we selected in Suppplementary Table 6 and were differentially expressed (*p* < 0.05). The color scale shown on the top illustrates the relative expression level of the indicated miRs across all samples: red denotes high expression levels, whereas green denotes low expression levels. Significant GO enrichment analysis and KEGG pathway analysis (**C**) predicted the most likely Biological Process (BP), Cellular Component (CC), Molecular Function (MF), and pathways. The vertical axis is the GO or pathway category, and the horizontal axis is the –log10 *p* value of the GO category or the enrichment of pathways.

According to the miR microarray analysis, which was previously described for miRs expression, the discrepant expression of miRs between GCs Group and Control Group was detected in MSCs from patients GCs 1, 2, 3 and Con 1, 2, 3. As we mentioned in the materials and methods section, we choose those six samples from sample pools and made Control Group match each sample in GCs Group by matching 1:1 with control in age, gender, and osteonecrosis classification^20^. The characteristics and mark number of the samples are summarized in Supplementary Table 1. The free miR QC Tool software was applied for data summarization, normalization, and quality control. Log-log scatter plot image showed differential expression of miRs between sample GCs 1 and Con 1 (Fig. 2A and Supplementary Table 3 online), and there were 24 miRs overexpressed and 50 miRs underexpressed in GCs 1 MSCs when compared with Con 1 MSCs. In Supplementary Table 4 (online), there were 52 miRs overexpressed and 35 miRs underexpressed in GCs 2 MSCs when compared with Con 2 MSCs, and 21 miRs overexpressed and 39 miRs underexpressed in GCs 3 MSCs in Supplementary Table 5 (online). According to the previous design, differentially expressed miRs that appeared consistently in at least two pairs were selected in Supplementary Table 6 (online). In addition, the log-log scatter plot image for those miRs and the hierarchical clustering of differentially expressed miRs selected in Supplementary Table 6 (online) were shown in Fig. 2A,B.

On the basis of the results above, many factors were taken into consideration, including the consistency of miR microarray analysis and the previous reports related to miRs and cell proliferation, differentiation, and migration^21^,^22^,^23^. We focused more on those miRNAs which had already been reported playing an important role in various kinds of cellular biology processes, such as miR-708, miR-25, miR-20b, and miR-30a. And we also eliminated some miRNAs which had one major data contrast in those three pairs, such as miR-3180-3p, miR-4304, miR-4486 and miR-4487. We then selected two overexpressed miRs (hsa-mir-483-5p and hsa-mir-708) and six underexpressed miRs (hsa-mir-92a-1, hsa-mir-20b, hsa-mir-25, hsa-mir-486-3p, hsa-mir-30a, and hsa-mir-106b) from the GCs groups for further verification, and we predicted that some of these miRs may play important roles in the development of GC-ONFH by MSC dysfunction.

### GCs induce high miR-708 expression in MSCs *in vivo* and *in vitro*

**Figure 3:**
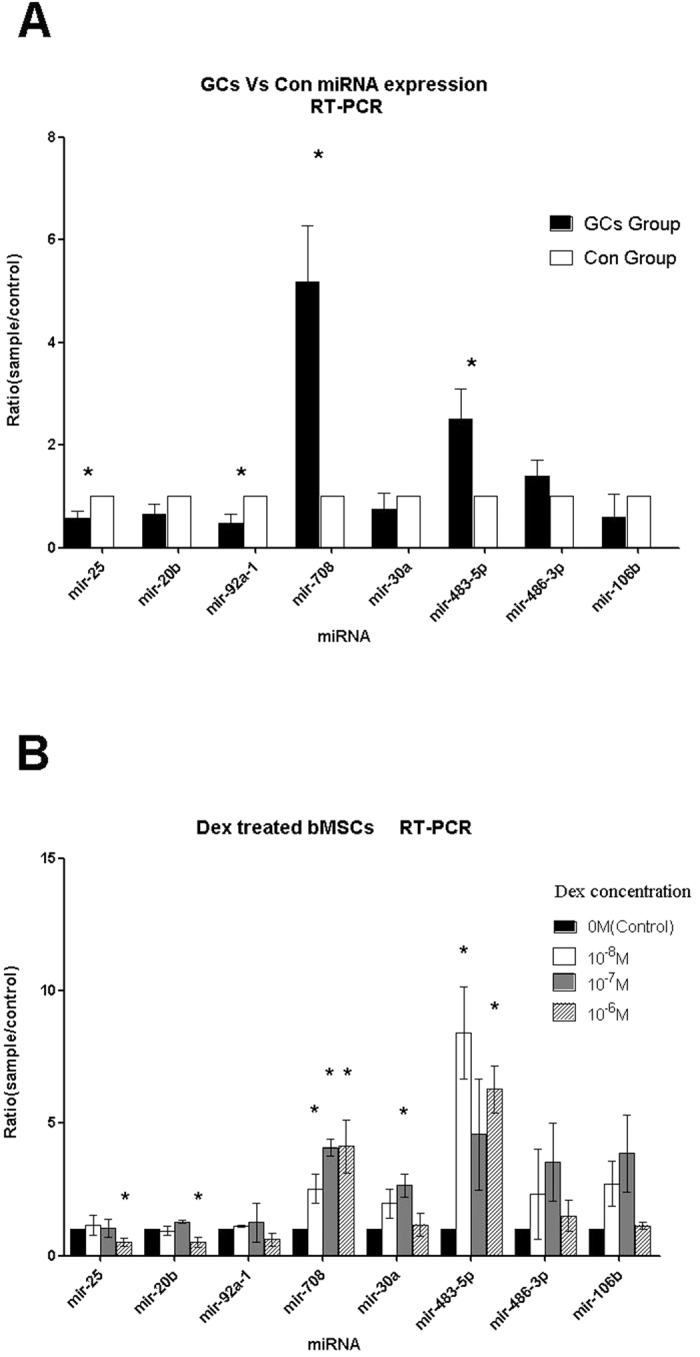
GCs induce high miR-708 expression in MSCs. The RT-PCR verification after Microarray analysis and GC intervention analysis verified and screened our miRs selected by microarray analysis for further studies. The results showed miR-708 high expression *in vivo* (**A**) and *in vitro* (**B**), which were stabilized and consistent with our microarray analysis. In Histogram A, miRs are listed on the X-axis, and the Y-axis refers to the relative expression levels of miRs between GCs Group MSCs and Con Group MSCs. The symbol * means *p* < 0.05 by significant paired *t*-test. Histogram B showed the RT-PCR results in the experiments with gradient concentration Dex treated MSCs *in vitro*. The symbol * means *p* < 0.05 by significant paired *t*-test. The legend in the top right corner shows the gradient concentration (0 M, 10^−8^ M, 10^−7^ M, and 10^−6^ M) of Dexamethasone.

To confirm the identification of these differentially expressed miRs, we used qRT-PCR to detect the expression levels of the eight miRs we selected by microarray analysis. We tested the expression of miRs between GCs Group and Control Group in MSCs samples from patients GCs 4, 5, 6 and Con 4, 5, 6, which were picked from sample pools and shown in Supplementary Table 1 (online) with 1:1 matched controls. The purity and integrity of the total RNA were tested by spectrophotometry and gel-electrophoresis, and a histogram was done to show the results of RT-PCR after comparing those three pairs (Fig. 3A). RT-PCR results showed that hsa-mir-25, hsa-mir-92a-1, hsa-mir-708, and hsa-mir-483-5p were significantly differentially expressed (*p* < 0.05) between GCs Groups and Con Groups in accordance with the microarray analysis above. However, the expression of the other four miRs showed no statistical difference.

To confirm the relationship between GC use and the expression change of the selected miRs further, we designed this experiment *in vitro*. qRT-PCR results (Fig. 3B) of the miRs after inducing gradient concentration Dex showed the following results. After treatment by Dex, the expression changes of hsa-mir-708 and hsa-mir-483-5p in MSCs were in agreement with the previous results of the microarray analysis and qRT-PCR verification precisely, and Dex induced the expression of hsa-mir-708 significantly in a dose-dependent manner. Furthermore, the expression of hsa-mir-25 and hsa-mir-20b were decreased at a Dex concentration of 10^−6^ M. These results above demonstrated that only miR-708 was highly expressed *in vivo* and *in vitro* by GCs. Therefore, the miR-708 was considered as the chosen miR for further target validation and functional verification because of its stable and significant expression change in all the experiments above.

### SMADs were predicted as the target of miR-708

To define the possible function of selected miRs in MSCs dysfunction, we used computational predictions to identify possible targets by online software. MiRNAs exert their effects mainly through binding to the untranslated regions of target gene mRNAs. Only the results of hsa-mir-708 were shown here as it was our gene of interest. MiR target prediction was determined using the online software which we showed in the method section below. There were 7341 genes put forward as the putative targets of hsa-mir-708 by at least one of those 10 programs shown in Supplementary Table 7. SUM represented the times of repetition in the prediction results, and we paid more attention to genes that got a higher SUM value (≥4), which mean a more reliable relationship of the predictions (Supplementary Table 8).

To organize those possible target genes into hierarchical categories and identify their potential regulatory network, GO enrichment analysis was performed to identify biological processes. The significance of enrichment of those genes was scored using a weighting algorithm. A two-sided Fisher’s exact test and a chi-square x^2^ test were used to classify the GO category, and the *q*-value was calculated to correct the *p*-value. We chose only GOs that had a *p*-value of <0.001^24^. GO enrichment analysis of those genes in Suppplementary Table 8, which were predicted as possible targets of miR-708 by online software, was shown in Fig. 2C. And the consequence was divided into three parts, including Biological Process (BP), Cellular Component (CC), and Molecular Function (MF). We then listed the components that had higher enrichment scores by online software MAS 3.0. The results predicted that miR-708 and its target genes may play an important role in regulation of transcription, cell cycle arrest, negative regulation of cell proliferation, and positive regulation of transforming growth factor beta (TGF-beta) receptor signaling pathway (Supplementary Table 9). These regulating effects were mostly located in the nucleus and cytoplasm. Simultaneously, the results of KEGG pathway analysis (Fig. 2C) showed significant correlation between some pathways and the target genes of hsa-mir-708; these were cell cycle, a number of cancer, p53 signaling pathway, and TGF-beta signaling pathway.

**Figure 4:**
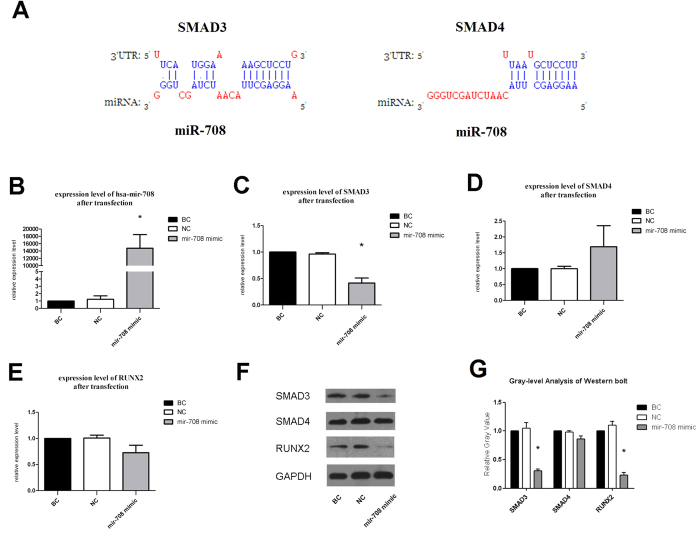
The transfection of miR-708 mimic markedly decreased the expression levels of SMAD3 and RUNX2 in MSCs. Schematic (**A**) shows the potential hsa-mir-708 binding site at the 3′-UTR of SMAD3 and SMAD4 as predicted. Histograms show RT-PCR results (**B–E**) and blot, gray images of western blot analysis with 50 nM (**F,G**) at 48 h after transfection. In the histograms the Y-axis refers to the relative expression levels of miRs and mRNAs compared with BC group, and the treatments are listed on the X-axis. The symbol * means *p* < 0.05 by significant paired *t*-test. Western blot was done three times. The gray level of the images shows the expression levels of proteins, which illustrated the function of miR-708 in TGF-beta signaling pathway and cell differentiation capacity.

By considering the results of GO enrichment analysis and the signaling pathways listed in KEGG, we focused on miR-708 and some of its potential target genes. Our prediction suggested that the expression change of miR-708 may influenc the expression level of its target genes, and result in MSCs dysfunction in the end. Thus, we again searched the genes listed in Supplementary Table 8. Among the target genes predicted by those algorithms, we selected SMAD3 and SMAD4 for they ranked highly among the predicted genes and had been reported to function as an important regulator of cell differentiation^25^,^26^. According to the principle of complementary pairing, the potential hsa-mir-708 binding sites at the 3′-UTR of SMAD3 and SMAD4 were found by prediction software (Fig. 4A). The binding type of SMAD3 and SMAD4 were respectively 8mer (pos 2) and 7mer (pos 1). Taken together, we predicted that hsa-mir-708 may directly regulate the expression of SMAD3 or SMAD4 through the binding site in the 3′-UTR. All the predictions needed to be confirmed by further verification in MSCs.

### The transfection of miR-708 mimic markedly decreased the expression levels of SMAD3 and RUNX2 in MSCs

As we predicted above, hsa-mir-708 was very likely to target SMAD3 or SMAD4, which are both involved in TGF-beta signaling pathway and osteogenic differentiation of MSCs. Much research on the relationship and interactional mechanism among SMADs, RUNX2 and TGF-beta signaling pathway has been reported recently. SMAD3 interacts with RUNX2 and confers functional TGF-beta stimulation of collagenase-3 expression, then effects osteoblast differentiation^26^. Further studies show that RUNX2 dose not directly interact with SMAD3 but rather recruits p300/CBP or histone deacetylase (HDAC) which is physically associate with both RUNX2 and SMAD3^27^. Guiqian Chen *et al*. state that the coordinated activity of Runx2 and TGF-β/BMP-activated Smads is critical for formation of the skeleton. Recent advances in molecular and genetic studies using gene targeting in mice enable a better understanding of TGF-β/BMP signaling in bone and in the signaling networks underlying osteoblast differentiation and bone formation^28^. Spontaneously for target verification, we used RT-PCR and Western blot analysis to test the expression levels of SMAD3, SMAD4, and RUNX2 after the transfection of miR-708 mimic in normal MSCs and miR-708 inhibitor in GC-MSCs. The survival rate of MSCs after transfection could have been up to 90% in our conditions at the concentration of 50 nM based on our early experiments.

The results showed that MSCs transfected with miR-708 mimic at 48 h significantly increased the expression level of hsa-mir-708 in normal MSCs when compared with NC Group and BC Group (Fig. 4B). MSCs transfected with miR-708 mimic also decreased the expression levels of SMAD3 and RUNX2 as demonstrated by RT-PCR (Fig. 4C–E), and markedly decreased the protein expression levels of SMAD3 and RUNX2 by Western blot analysis at 48 h after the transfection in 50 nM concentration (Fig. 4F). Gray-level analysis of western blot images demonstrated that the decrease of the protein expression levels of SMAD3 and RUNX2 were statistically significant (*p* < 0.05) (Fig. 4G). However, the decrease of the protein expression levels of SMAD4 was not obvious.

### MiR-708 directly targeted the 3′-UTR of SMAD3

**Figure 5:**
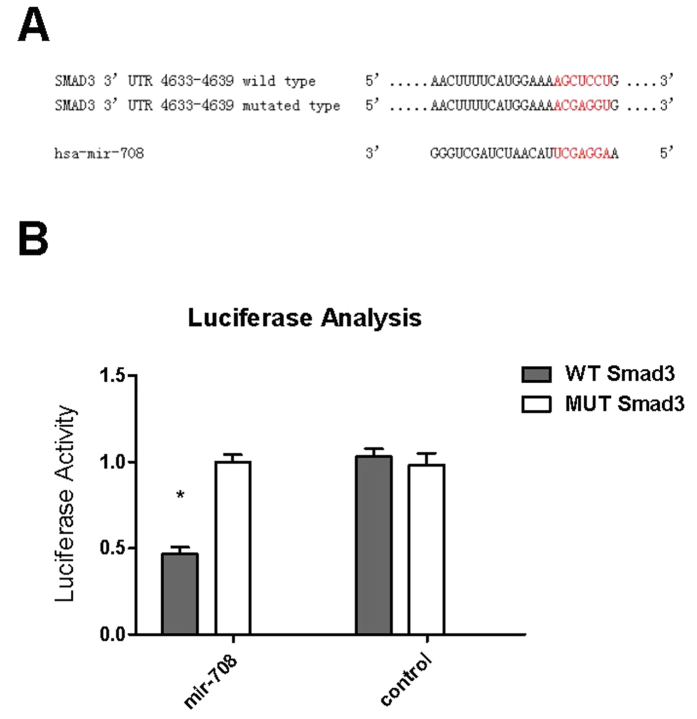
MiR-708 directly targeted the 3′-UTR of SMAD3. The 3′-UTR of SMAD3 has the putative binding sites with miR-708 (**A**). The psiCHECK2-SMAD3 3′-UTR WT and psiCHECK2-SMAD3 3′-UTR mutated were co-transfected with miR-708 mimic negative control or miR-708 mimic into MSCs cells (**B**). After 48 hours of transfection, luciferase activities were measured (n = 3). The data shown represent mean standard deviation, *p* < 0.05, n = 3. Abbreviations: NC, negative control; SMAD3, SMAD family member 3; UTR, untranslated regions.

To determine whether miR-708 could directly regulate the 3′-UTR of SMAD3, we generated luciferase reporter constructs that contained the putative miR-708 binding sites in the 3′-UTR of SMAD3 and the mutated putative miR-708 binding sites (Fig. 5A). Consistent with our previous results, the reporter assays demonstrate that co-transfection of miR-708 mimic inhibited reporter activity of pciCHECK2-SMAD3 relative to co-transfection of mimic negative control or psiCHECK2-mut-SMAD3, These data indicate that SMAD3 is a direct target of miR-708 (Fig. 5B).

### MiR-708 recovered the osteogenic differentiation capability of GC-treated MSCs

**Figure 6:**
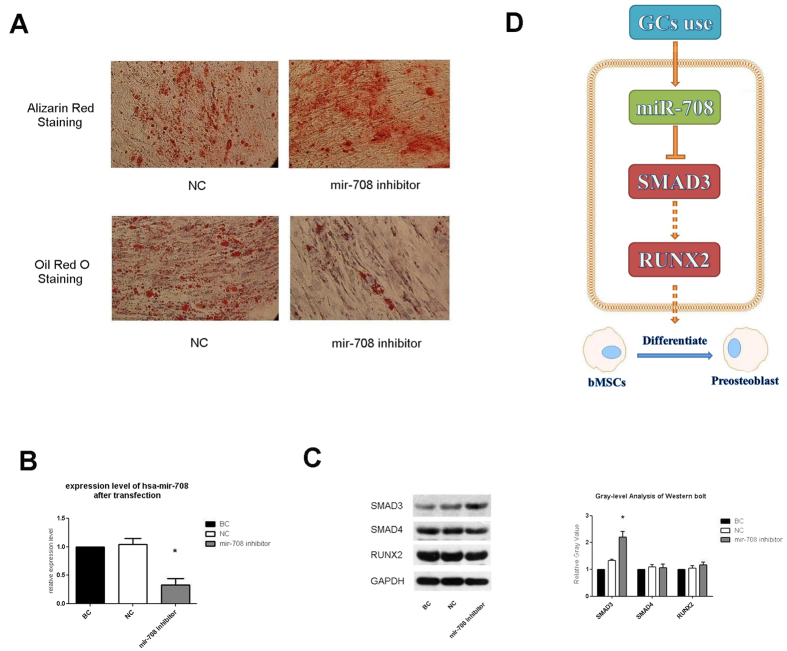
GC-MSCs regained their osteogenic differentiation capability by repressing the expression level of miR-708. Microscope images (**A**), RT-PCR results (**B**), and blot, gray images of western blot analysis at 48 h (**C**) after transfection are shown. Glucocorticoid treatments induce the high expression of miR-708 which was considered as the chosen miR for further target validation and functional verification because of its stable and significant expression change in all the experiments above. Our findings provided evidence for GC-induced necrosis of femoral head via miR-708 directly regulating SMAD3 expression and indirectly regulating RUNX2 expression (**D**). In this SMADs-depended signaling pathway, SAMD3 and RUNX2 interact with other transcription factors to trigger target genes expression and lead to functional change.

To determine the effects of miR-708 on MSCs ostesgenic differentiation, we transfected negative control and miR-708 inhibitor into each groups of GC-MSCs for three days, and then treated with osteoblast differentiation medium and adipogenic differentiation medium for additional 14 days (Fig. 6A). GC-MSCs with upregulated miR-708 showed inhibition of osteogenic differentiation capabilities, which presented as inhibition of cell functions. However, GC-MSCs with downregulated miR-708 showed enhancement of osteogenic differentiation and inhibition of adipogenesis differentiation capability (Fig. 6A), which indicated that the GC-MSCs regained their osteogenic differentiation capability by recovering the expression level of miR-708 in cells. As above, transfection with miR-708 inhibitor at 48 h suppressed the expression level of hsa-mir-708 in GC-MSCs (Fig. 6B). Furthermore, transfection with miR-708 inhibitor increased the expression levels of SMAD3 as exhibited by Western blot when compared with NC Group and BC Group at 50 nM concentration (Fig. 6C).

We found that no changes were observed in RUNX2 after inhibition of mir-708 at 48 h after the transfection. However, Hiroshi Kaji *et al*. previously reported that SMAD3 decease the level of RUNX2 at confluent MC3T3-E1 cells corresponding to 7 d culture, on the other hand SMAD3 overexpression enhanced the level of RUNX2 at 14 d cultures^29^. These studies suggested that SMAD3 play an important role in the control of mineralization partly though RUNX2, which depend upon cell differentiation stage. Therefore we attributed the un-significant expression change of RUNX2 to detection time point.

However, the expression change of SMAD4 was not significant (Figs 4D and 6C). Therefore, according to the experiments above we considered that the interrelation between mir-708 and SMAD4 may not exist in fact, but only in our forecast.

In conclusion, these data suggested that the GC use led to overexpression of hsa-mir-708 in human MSCs *in vivo* and *in vitro*. The upregulated miR-708 suppressed the expression of SMAD3, and finally resulted in MSC dysfunction of osteogenic differentiation and the development of GC-ONFH (Fig. 6D).

## Discussion

Bone is formed through two distinct phases, one of which is intramembranous ossification. In this process bones are shaped directly from condensations of mesenchymal cells without a cartilage intermediate^30^. Bone is continuously remodeled through life *in vivo* and an imbalance in this process can result in bone disease, such as osteonecrosis. The integrity and function of bone are maintained by an exquisite balance between osteoclasts and osteoblasts. However the decrease of the osteogenic potential of mesenchymal stem cells (MSCs) can break this balance^31^, which lead to the decrease of osteogenesis and accumulation of adipogenesis and osteonecrosis in bones. Recently research showed high concentrations of Dex exerted negative effects on MSC proliferation and apoptosis^32^. Furthermore, Dex suppresses on osteogenic differentiation of MSCs^2^. Consistent with previous studies, our results confirmed that the osteogenic differentiation capacity of GC-MSCs was subdued and the adipogenesis differentiation capacity was enhanced when compared with normal MSCs. Interestingly, glucocorticoid induced osteonecrosis develops in 9–40% of patients receiving long-term exposure to high doses therapy, although it may also occur with short-term, some without glucocorticoid induced osteoporosis^33^.

In recent years, miRs have emerged as new regulatory molecules of gene expression that play critical roles in stem cell function^34^. Most miRs have been described as signaling network nodes that participate in modulating intricate osteoblastic differentiation processes. For example, miR-21, miR-26a, and miR-196 have been shown to target this pathway and play an important roles in the osteogenic differentiation of MSCs^35^,^36^,^37^. More importantly, some studies have demonstrated that miR-188 and miR-194 are key regulators that switch between osteoblast and adipocyte differentiation between osteogenesis and adipogenesis of BMSCs^38^,^39^. In our study, microarray analysis revealed the differential expression profiling of miRs between MSCs of GCs Group and Control Group, including mir-708. We then used qRT-PCR to verify these expression changes of those miRs selected by microarray analysis, and further experiments showed that GCs induce high miR-708 expression in MSCs both *in vivo* and *in vitro*. Similarly, it has been demonstrated that glucocorticoid treatments induce the high expression of miR-708^40^. We must also declare that some other miRs which showed differential expression in our experiments, such as miR-20b or miR-25, may play an important role in osteogenic differentiation of human stem cell^41^. And they may possibly act synergistically with mir-708 in this mechanism. But in consideration of the inconsistent results among miR microarray analysis, quantitative RT-PCR verification and glucocorticoid intervention *in vitro*, only mir-708 was considered as the chosen miR because of its stable and significant expression change in all the experiments above.

TGF-beta signaling and BMP signaling pathway were the two most important pathways in osteoblastic differentiation. Through putative targets prediction, we found that miR-708 may regulate SMADs, which are an important component of TGF-beta signaling. TGF-beta initiates signaling by binding and activating the membrane-anchored type II and type I receptor Ser/Thr kinases, which subsequently phosphorylate the effectors SMAD2/3. Phosphorylated SMAD2/3 can then form complexes with the common SMAD, SMAD4, and translocate into the nucleus, which results in the activation or repression of downstream target genes to regulate cell function and metabolism^42^. Following TGF-beta/BMPs induction, the SMADs converge at the RUNX2 gene to control mesenchymal precursor cell, and the coordinated activity of RUNX2 and TGF-beta/BMPs-activated SMADs is critical for formation of the skeleton^28^. Our functional verification at the cellular level showed that the transfection with miR-708 mimic significantly increased the expression level of hsa-mir-708 in normal MSCs when compared with NC Group and BC Group. Transfections with miR-708 mimic also decreased the expression levels of SMAD3 and RUNX2 by RT-PCR and markedly decreased the protein expression levels of SMAD3 and RUNX2 as demonstrated by Western blot analysis. Furthermore, the luciferase reporter assay determined that miR-708 could directly regulate the 3′-UTR of SMAD3. Our findings provide evidence for GC-induced necrosis of femoral head via miR-708 directly regulating SMAD3 expression. Because RUNX2 is likely subjected to multiple levels of regulation, we tried to identify whether RUNX2 would be a direct target of miR-708. There was no direct connection between them (data not shown). A series of researches below comfirmed that SMAD3 was really the key factor to osteogenic differentiation, and there was some intermediary working as the linkage between SMAD3 and RUNX2. Class IIa HDACs act as corepressors for TGF-β/Smad3-mediated transcriptional repression of Runx2 function in differentiating osteoblasts and are cell-intrinsic regulators of osteoblast differentiation^43^. Filamin B inhibits Runx2 activity through the Smad3 pathway by regulating Smad3 phosphorylation^44^. Dingwall M *et al*. indicate that Smad3 is an important mediator of retinoic acid activity during mesenchymal stem cell differentiation by interfering with CCAAT/Enhancer Binding Protein beta (C/EBPβ) where it binds to a negative regulatory element within the RUNX2 promoter and inhibits its expression^45^. And other studies show that the regulation of RUNX2 activity by SMAD3 is mediated by controlling the expression of Msx2, Menin or Smad-binding element (SBE) under TGF-beta-stimulated conditions^27^,^46^,^47^. Therefore, we inferred that SMAD3 played a major role as the induction of RUNX2 expression, and regulated osteogenic differentiation.

Giving that GC use leads to the dysfunction of MSCs, we considered whether decreasing the expression level of miR-708 could recover the osteogenic differentiation capability of GC-MSCs. In our study, we transfected negative control and miR-708 inhibitor into each groups of GC-MSCs, and the MSCs showed enhancement of osteogenic differentiation and inhibition of adipogenesis differentiation capability by morphological staining. Transfection of miR-708 inhibitor also rescued the expression of SMAD3 as shown by qPT-PCR and Western blot analysis, and on the other hand, reaffirmed that SMAD3 msut be the target of mir-708. There are other studies that have demonstrated that miR-29a and miR-216a can protect against glucocorticoid-induced bone loss and rescue dexamethasone suppression of osteogenesis^2^,^48^, and this result is very similar to that of miR-708. Our findings demonstrate that miR-708 inhibits osteogenic differentiation and targeting miR-708 rescues the suppressive effects of Dex on osteogenic differentiation and adipogenesis differentiation of MSCs through the SMAD3 pathway.

In summary, we are the first to confirm that miR-708 is inversely correlated with osteonecrosis, and targeting miR-708 can not only promote osteogenic differentiation *in vitro*, but also can effectively antagonize the suppression of Dex on osteoblast differentiation and adipogenesis differentiation through increasing SMAD3 expression, which may result in the interaction between SMAD3 and RUNX2, and the activation of the TGF-beta signaling pathway. Modulation of miR-708 signaling will be an innovative method for alleviating the deleterious effect of glucocorticoid treatment on bone mass homeostasis (Fig. 6D).

## Materials and Methods

### Source and grouping of MSCs samples

With approval from the ethics committee of Union Hospital and written informed consents, the bone marrow samples of patients with GC-ONFH (n = 13) and patients with ONFH after a previous fracture of the femoral neck (n = 21) were obtained during operation. Those patients underwent total hip arthroplasty (THA) between August 2013 and June 2014 at Wuhan Union Hospital, Huazhong University of Science and Technology. All experimental protocols were approved by the Ethics Committee at Tongji Medical College, Huazhong University of Science and Technology, and informed consent was obtained from all subjects. All experiments were performed in accordance with the approved guidelines and regulations. We then stipulated specific inclusive criteria for those patients to build comparison relationships between these samples. Each chosen individuals satisfied the following conditions: age from 20 to 50, no smoking or alcoholism history, no underlying diseases, including hypertension, diabetes mellitus, hyperlipemia, cardiopathy, infectious disease, or congenital disease. Those patients were definitively diagnosed to have osteonecrosis of femoral head by image examination before operation and pathological diagnosis after surgery. For GC-induced ONFH, the steroid exposure threshold was 1800 mg GCs or its equivalent over four weeks^49^, and for control patients there was no GC use history. And these samples were brought into sample pools for further studies.

After screening, six patients with GC-ONFH and 11 patients with previous fractures of femoral neck were assigned to GCs Group and Control Group accordingly from sample pools. We then choose six patients from Control Group to match each patient in GCs Group, by matching 1:1 with control in age, gender, and osteonecrosis classification^20^. Their characteristics and mark number are summarized in Supplementary Table 1.

### Isolation and culture of human MSCs

Our bone marrow samples (5–10 ml) were procured from the proximal end of the femur after inserting the tapered awl into the femoral canal during THA^11^. Human MSCs were isolated and cultured as the previously reported^9^. Mononuclear cells from the bone marrow were separated by an equal volume of 1.073 g/ml Percoll solution (Sigma, USA) at 2000 rpm for 30 min. Then, cells were collected and resuspended in complete culture medium of low glucose Dulbecco’s modified Eagle’s medium (HyClone, USA), 10% fetal bovine serum (Gibco, USA), 100 U/ml penicillin, and 100 mg/ml streptomycin. The cells were seeded at 5000 cells/cm^2^ in 25-cm^2^ culture flasks (Corning, USA) and incubated at 37 °C with 5% carbon dioxide and 95% humidity. After three days, the non-adherent cells were removed and cultures re-fed every three days. Cells were cultured for 10–12 days until they attained a confluence ≥80%, then were digested using a solution of 0.25% trypsin and 0.02% EDTA (Invitrogen) and replated at a 1:2 dilution for the initial subculture. After this treatment three times, MSCs were ready for further experiments. These cells were expanded by successive subculture for about 7–8 passages keeping the biological characteristics of primary culture cell, with each passage after 3–5 days.

### Cell identification

We performed the immunophenotype analysis on MSCs by flow cytometry. MSCs were digested and resuspended in PBS (HyClone, USA) at a concentration of 10^6^ cells/ml and washed twice with PBS. We used 500 μl cell resuspension solution and stained the cells with 5 μl mouse anti-human CD29-PE, CD34-FITC, CD44-APC, and CD45-PerCP antibodies^50^ for 30 min at room temperature. After rinsing twice with cleaning solution and resuspending in 500 μl of cleaning solution, the cells were analyzed by flow cytometry to identify human MSC phenotypes.

For osteogenic induction, the MSCs were treated in human mesenchymal stem cell osteogenic differentiation medium (catalog No. HUXMA-90021; Cyagen, China) for 7–21 days. Medium was changed every two days. After differentiation, the osteogenic differentiation medium was removed from the wells, and the cells were fixed with 2 ml of a 4% formaldehyde solution in PBS for 30 minutes. Then, the cells were stained with 1 ml of alizarin red S solution (catalog No. S0141; Cyagen, China) for 30 minutes. The images were visualized under a light microscope (Leica DMIRB, Heidelberg, Germany).

To induce adipogenesis, the MSCs were treated in human mesenchymal stem cell adipogenic differentiation medium (catalog No. HUXMA-90031; Cyagen, China) for 7–21 days, changing the medium every two days. After differentiation, we removed the medium from each well and fixed the cells with 2 ml of a 4% formaldehyde solution for 30 minutes. Then, the cells were stained with 1 ml of oil red O working solution (catalog No. S0131; Cyagen, China) for 30 minutes and visualized with a light microscope (Leica DMIRB).

### RNA extraction

Total RNA of MSCs was extracted using Trizol reagent (TAKARA, Japan) through standard protocols according to the instructions of the manufacturer. RNAs were resuspended in DEPC treated ddH_2_O, and then the purity and integrity of the total RNA were tested by spectrophotometry and gel electrophoresis.

### Mir microarray analysis

The total RNA samples of GCs 1–3 and Con 1–3 were sent to Beijing CapitalBio Corporation (China) for processing. MiRs were enriched from total RNA with mirVana^®^ miR Isolation Kit (Ambion, Foster City, CA, USA) and labeled with FlashTag^TM^ Biotin RNA Labeling Kit. Then, they were hybridized on a miR microarray (Affymetrix 3.0). Images of the miR microarrays were acquired by Affymetrix GeneChip^®^ Scanner 3000. The figure signals were transformed to digital signals using image analysis software (LuxScan 3.0; CapitalBio) with the free miR QC Tool software for data summarization, normalization, and quality control.

Differentially expressed miRs between GCs Group and Con Group were analyzed using the SAM software. Samples were 1:1 matched as previously described. MiRs that fulfilled the criteria of *p* value < 0.05 and fold change ≥2 or fold change ≤0.5 between each pair of samples were considered to be significantly different. Heat map was constructed using the Cluster 3.0 package software. Among the comparison of these three pair, differentially expressed miRs that appeared in at least 2 pairs were selected for further experiments.

### Real-time RT-PCR

RNAs were analyzed by quantitative RT-PCR as previous described^51^. For the detection of mature miRs, verification of microarray analysis and miRs function, total RNAs were reverse transcribed using specific Bulge-Loop^TM^ miR qRT-PCR primer (Ribobio Co., Ltd, Guangzhou, China) and the PrimeScript^TM^ RT reagent Kit (TAKARA, Code No. RR037A) according to recommended guidelines of the manufacturers. The mRNA transcripts were quantified by real-time PT-PCR using the SYBR^®^ Premix Ex Taq^TM^ (TAKARA, Code No. RR420A) and the Applied Biosystems StepOnePlus^TM^ Real-Time PCR System. The thermo-cycling program was set as: 95 °C for 10 min to denature DNA templates, followed by 40 cycles at 95 °C for 15 s, 60 °C for 30 s and 72 °C for 30 s, finally an additional dissociation step to ensure the specificity of amplification. All samples were also run on a 3% agarose gel to confirm specificity. Respective β-Actin and U6 small nuclear RNA genes were used as endogenous normalization controls for protein-encoding genes and miRs to be detected. Primers used for amplification are listed in Supplementary Table 2.

### Glucocorticoid treatment of MSCs

To investigate the expression change of miRs in MSCs caused by glucocorticoids intervention *in vitro*, the cells from three randomly picked patients from Control Group were seeded into 6-well plates at 10^4^ cells/cm^2^ after three culture passages, and were grown to about 70% confluences over 24–48 h in complete culture medium. Dexamethasone (Sigma, USA) was tested *in vitro* at gradient concentrations (0 M, 10^−8^ M, 10^−7^ M, and 10^−6^ M)^52^. Drugs were added to the complete culture medium according to the gradient concentration above; 0 M as control were treated by isopyknic normal saline (NS). After three days of intervention, the culture medium was discarded and new medium with gradient concentration Dex was added. The cells were harvested after 7 days of Dex intervention for RT-PCR. By this means, we obtained GC-MSCs as a cell model with 10^−6^ M Dex intervention *in vitro*.

### Mir target prediction

MiR target prediction was determined using the online software, DIANA-microT v3.0 (http://diana.cslab.ece.ntua.gr/microT/)^53^, TargetScan Human 6.2 (http://targetscan.org/)^54^, miRanda 3.0, miRWalk, RNAhybrid, PicTar 4, PicTar 5 (http://pictar.bio.nyu.edu), and other common software (http://www.umm.uni-heidelberg.de/apps/zmf/mirwalk/micrornapredictedtarget.html) for miRs target prediction. The miRBase website (http://www.mirbase.org/) was used to search for predicted targets. Then, those predicted target lists were analyzed for association with Gene Ontology (GO) terms and KEGG pathway analysis using online software MAS 3.0 (Molecule Annotation System, CapitalBio, Beijing, http://bioinfo.capitalbio.com/mas3/)^55^, which is based on the integration of public databases. Two underexpressed miRs (hsa-miR-25, hsa-miR-92a-1) and one overexpressed miR (hsa-miR-708) were selected from the experiments above for target verification.

### Functional analysis and target verification

We selected hsa-miR-708 for target verification and functional analysis. Studies were performed with miR-708 precursor molecules (miR-708 mimic), miR-708 inhibitor, miR-708 precursor molecules negative control (NC), and blank control (BC). The miR-708 mimic, miR-708 inhibitor, and NC were designed and synthesized by GenePharma Company (http://www.genepharma.com/) and the base sequences were shown as follows: miR-708 mimic sense 5′-AAGGAGCUUACAAUCUAGCUGGG-3′, miR-708 mimic antisense 5-CAGCUAGAUUGUAAGCUCCUUUU-3, miR-708 inhibitor sense 5′-AAAAGGAGCUUACAAUCUAGCUG-3′, miR-708 inhibitor antisense 5′-CAGUACUUUUGUGUAGUACAA-3′, NC sense 5′-UUCUCCGAACGUGUCACGUTT-3′, NC antisense 5′-ACGUGACACGUUCGGAGAATT-3′.

To ensure our cells used in the functional analysis were normal and not affected by GCs, the MSCs from three patients of the Control Group were randomly selected and seeded into 12-well plates at 10^4^ cells/cm^2^ after three culture passages, and were grown to about 70% confluence over 24–48 h in complete culture medium. Then, cells were transfected using Lipofectamine 2000 reagent (Invitrogen, USA) according to the manufacturer’s protocol with 50 nM miR mimic and NC. At 48 h after transfection, the cells were lysed for RNA extraction and the expression levels of miR-708 and the chosen genes in Supplementary Table 2 were detected by RT-PCR as described above. Proteins from cells were prepared for Western blot analysis at the same time.

For Western blot, cells were lysed in RIPA buffer (Sigma, USA) plus protease inhibitors. Equal amounts of protein were loaded and separated on 10% sodium dodecyl sulfate polyacrylamide gels (SDS-PAGE) and transferred onto polyvinylidene fluoride (PVDF) membranes, and then blocked by incubation with 5% fat-free milk in PBST buffer at 25 °C for 1 h. The membranes were probed overnight with the following monoclonal primary antibodies: SMAD3 antibody (1:1000, CST #8685), SMAD4 antibody (1:1000, Abcam #ab40759), and RUNX2 antibody (1:1000, Abcam #ab76956). Then, the membranes were incubated with horseradish peroxide-conjugated secondary antibodies at 25 °C for 1 h. GAPDH was used as loading control. The blots were visualized using an enhanced chemiluminescence kit (Pierce, IL, USA) according to the manufacturer’s recommended instructions. Additionally, GC-MSCs (10^−6^ M Dexamethasone) were also transfected with miR inhibitor, mimic, and NC, and the relevant RT-PCR, western blot analysis, differentiation, and morphology staining of the GC-MSCs 48 h after transfection were performed as mentioned above for functional analysis.

### Luciferase reporter assay

The luciferase 3′-UTR reporter constructs were generated by introducing the 3′-UTR of the SMAD3 gene carrying the putative miR-708 binding site into the luciferase reporter vector. We first amplified the SMAD3 3′-UTR sequences by PCR using specific primers. The primers used for SMAD3 WT: Fw: 5-CCGCTCGAGGTTCCCCCAAAATAAACGTGTCCCC-3′ (underline represents XhoI site), Rev: 5′-ATTTGCGGCCGCTCAGACTGAGCTCCTGGCACAGGAG-3′ (underline represents NotI site). For SMAD3 3′-UTR mutation, the primers used were: Fw: 5′-ATTCTAGGCGATCGCTCGAGGCAGAAGTAATGTATACTCTAGTAT-3′, Rev: 5′-AACGAATTCCCGGGCTCGAGTCAGACTGAGCTCCTGGCACACCTCGTTTTCC-3′ (underline represents mutation sequence). The PCR products were cloned into psiCHECK2 (Promega, USA). All constructions were confirmed by sequence analysis. 293T cell were transferred to 24-well plates at 70% confluence 24 h before transfection. All transfections were conducted using Lipofectamine 2000 (Invitrogen, USA). The psiCHECK2-SMAD3 3′-UTR WT and the psiCHECK2-SMAD3 3′-UTR mutated were used as reporter constructs and were co-transfected into cells with hsa-miR-708 mimics or mimics negative control at a final concentration of 50 nM. Transfections were performed in duplicate and all experiments were repeated several times. After 48 hours, firefly luciferase activity and luciferase activity were determined in cell lysates according to the manufacturers’ recommended protocols (Dual-Luciferase Reporter Assay System).

### Statistics

All results were presented as the mean ± SD from at least three independent experiments. The statistical analysis was carried out using computer software SPSS V 21.0. Comparisons were done using the paired t-test, a value of *p* less than 0.05 was considered statistically significant. GraphPad Prism 5 software was used for statistical analysis charting.

## Additional Information

**How to cite this article**: Hao, C. *et al*. MiR-708 promotes steroid-induced osteonecrosis of femoral head, suppresses osteogenic differentiation by targeting SMAD3. *Sci. Rep.* **6**, 22599; doi: 10.1038/srep22599 (2016).

## Supplementary Material

Supplementary Information

Supplementary Tables
