# Supplementary material for: MiR-708 promotes steroid-induced osteonecrosis of femoral head, suppresses osteogenic differentiation by targeting SMAD3
Source: Sci Rep. 2016 Mar 2;6:22599. doi: 10.1038/srep22599 (PMC4773864; doi:10.1038/srep22599)
Supplement: Supplementary Information [file srep22599-s1.doc]

**MiR-708 promotes steroid-induced osteonecrosis of femoral head, suppresses osteogenic differentiation by targeting SMAD3**

Cheng Hao^1#^, Shuhua Yang^1^, Weihua Xu^1#^, Jacson K Shen^2^, Shunan Ye^1^, Xianzhe Liu^1^, Zhe Dong^1^, Baojun Xiao^1^ and Yong Feng^1.2^*

**AUTHOR AFFILIATIONS**

1 Orthopedic Hospital, Union Hospital, Tongji Medical College, Huazhong University of Science and Technology, Wuhan, Hubei, PR China

2 Sarcoma Biology Laboratory, Department of Orthopaedic Surgery, Massachusetts General Hospital and Harvard Medical School, 55 Fruit Street, Jackson 1115, Boston, Massachusetts 02114

^#^Cheng Hao and Weihua Xu contributed equally to this work

*Correspondence to: Yong Feng, Orthopedic Hospital, Union Hospital, Tongji Medical College, Huazhong University of Science and Technology, Wuhan, Hubei, PR China. Telephone: 86-27-8535-1627, Fax: 86-27-8535-1627, E-mail: fengyong1980@gmail.com

**ADDITIONAL INFORMATION**

**Supplementary information** accompanies this paper at http://www.nature.com/srep

**Competing financial interests:** The authors declare no competing financial interests.

**How to cite this article**:

This work is licensed under a Creative Commons Attribution 4.0 International License. The images or other third party material in this article are included in the article’s Creative Commons license, unless indicated otherwise in the credit line; if the material is not included under the Creative Commons license, users will need to obtain permission from the license holder to reproduce the material. To view a copy of this license, visit http://creativecommons.org/licenses/by/4.0/

**SUPPLEMENTARY TABLES**

Table 1. Characteristics and mark number of the chosen patients.

Table 2. RT-PCR Oligonucleotide Primers.

Table 3. Different expression of miRs between sample GCs1 and Con 1.

Table 4. Different expression of miRs between sample GCs2 and Con 2.

Table 5. Different expression of miRs between sample GCs3 and Con 3.

Table 6. Different expression of miRs in at least two pairs.

Table 7. Target gene prediction of hsa-mir-708 by online software.

Table 8. Target genes of hsa-mir-708 (SUM ≥ 4).

Table 9. GO enrichment analysis for the possible target genes of miR-708.
