# Supplementary material for: MiR-708 promotes steroid-induced osteonecrosis of femoral head, suppresses osteogenic differentiation by targeting SMAD3
Source: Sci Rep. 2016 Mar 2;6:22599. doi: 10.1038/srep22599 (PMC4773864; doi:10.1038/srep22599)
Supplement: Supplementary Tables [file srep22599-s2.pdf]

MiR-708 promotes steroid-induced osteonecrosis of femoral head, suppresses osteogenic differentiation by targeting SMAD3

Cheng Hao[1#], Shuhua Yang[1], Weihua Xu[1#], Jacson K Shen[2], Shunan Ye[1], Xianzhe Liu[1], Zhe Dong[1], Baojun Xiao[1] and Yong Feng[1.2*]

AUTHOR AFFILIATIONS

1 Orthopedic Hospital, Union Hospital, Tongji Medical College, Huazhong University of Science and Technology, Wuhan, Hubei, PR China

2 Sarcoma Biology Laboratory, Department of Orthopaedic Surgery, Massachusetts General Hospital and Harvard Medical School, 55 Fruit Street, Jackson 1115, Boston, Massachusetts 02114

[#]Cheng Hao and Weihua Xu contributed equally to this work

*Correspondence to: Yong Feng, Orthopedic Hospital, Union Hospital, Tongji Medical College, Huazhong University of Science and Technology, Wuhan, Hubei, PR China. Telephone: 86-27-8535-1627, Fax: 86-27-8535-1627, E-mail: fengyong1980@gmail.com

Table 1. Characteristics and mark number of chosen patients

mark number	age	gender	Ficat stage	GCs use history
GCs 1*	28	female	II	yes
Con 1*	32	female	II	no
GCs 2	46	male	II	yes
Con 2	48	male	II	no
GCs 3	39	male	III	yes
Con 3	35	male	III	no
GCs 4	31	male	II	yes
Con 4	27	male	II	no
GCs 5	45	female	IV	yes
Con 5	46	female	IV	no
GCs 6	36	male	III	yes
Con 6	37	male	III	no

* means GCs Group patient 1 and Control Group patient 1, and so on.

Table 2. RT-PCR Oligonucleotide Primers

RNA name	Primer sequence 5`→3`
β-Actin Forward	GTCCACCGCAAATGCTTCTA
β-Actin Reverse	TGCTGTCACCTTCACCGTTC
SMAD3 Forward	CCTCTCCAGCAATAATCCGAA
SMAD3 Reverse	TGCCCAATTTTCTTTACCAGT
SMAD4 Forward	ACTTGGCATCTCTACATTGTCC
SMAD4 Reverse	GCCACATCTATTTTGCTTGCT
RUNX2 Forward	GGACGAGGCAAGAGTTTCAC
RUNX2 Reverse	GAGGCGGTCAGAGAACAAAC

Table 3. Different expression of miRNAs between sample GCs1 and Con 1

ProbeSetID	GCs 1	GCs 1.call	Con 1	Con 1.call	Ratio	Ratio.call
24 miRNAs overexpression in GCs 1 MSCs						
hsa-miR-1207-5p_st	88.48672	P	40.86261	P	2.1655	P
hsa-miR-125a-3p_st	79.44888	P	33.82151	P	2.3491	P
hsa-miR-1268_st	122.9203	P	51.15377	P	2.403	P
hsa-miR-1268b_st	104.0176	P	51.75887	P	2.0097	P
hsa-miR-1275_st	376.8623	P	144.6312	P	2.6057	P
hsa-miR-1307_st	340.3954	P	158.3513	P	2.1496	P
hsa-miR-138-1-star_st	288.178	P	64.76934	P	4.4493	P
hsa-miR-138_st	3124.454	P	798.2524	P	3.9141	P
hsa-miR-1587_st	18.27409	P	7.309891	P	2.4999	P
hsa-miR-23a-star_st	256.4548	P	69.45473	P	3.6924	P
hsa-miR-25-star_st	18.09319	P	7.685211	P	2.3543	P
hsa-miR-3138_st	10.13937	P	2.18684	A	4.6365	P
hsa-miR-3141_st	149.2077	P	63.35411	P	2.3551	P
hsa-miR-3909_st	8.134102	P	3.663411	P	2.2204	P
hsa-miR-4298_st	519.0026	P	204.6377	P	2.5362	P
hsa-miR-4304_st	66.17074	P	13.61831	P	4.859	P
hsa-miR-4443_st	123.7881	P	58.70681	P	2.1086	P
hsa-miR-4487_st	25.30063	P	7.805188	P	3.2415	P
hsa-miR-543_st	6.484666	P	3.168579	P	2.0466	P
hsa-miR-615-5p_st	8.406401	P	3.925661	A	2.1414	P
hsa-miR-671-3p_st	28.62154	P	13.05424	P	2.1925	P
hsa-miR-770-5p_st	5.911391	P	2.71108	A	2.1805	P
hsa-miR-92a-1_st	6.98929	P	3.457691	A	2.0214	P
hsa-miR-941_st	11.43651	P	3.275517	A	3.4915	P
50 miRNAs underexpression in GCs 1 MSCs						
hsa-let-7i-star_st	2.109525	A	6.109076	P	0.3453	P
hsa-miR-1231_st	3.046926	A	10.18689	P	0.2991	P
hsa-miR-124_st	2.627133	A	35.00599	P	0.075	P
hsa-miR-125b-2-star_st	2.482835	A	6.993793	P	0.355	P
hsa-miR-1270_st	2.263109	A	4.773544	P	0.4741	P
hsa-miR-151-3p_st	13.52336	P	30.73665	P	0.44	P
hsa-miR-152_st	60.80596	P	122.6588	P	0.4957	P
hsa-miR-154_st	2.110948	A	4.677903	P	0.4513	P
hsa-miR-181a_st	167.1619	P	375.215	P	0.4455	P
hsa-miR-1908_st	272.2883	P	557.9352	P	0.488	P
hsa-miR-195_st	5.597762	P	15.59494	P	0.3589	P
hsa-miR-196b_st	2.253664	P	7.23118	P	0.3117	P
hsa-miR-214-star_st	3.530133	P	17.43221	P	0.2025	P
hsa-miR-224-star_st	6.469038	P	17.40992	P	0.3716	P

hsa-miR-2277-3p_st	7.726739	P	17.61835	P	0.4386	P
hsa-miR-3180-3p_st	7.582824	P	21.36118	P	0.355	P
hsa-miR-3185_st	106.2435	P	219.8447	P	0.4833	P
hsa-miR-3188_st	3.904307	A	7.862194	P	0.4966	P
hsa-miR-3195_st	33.32552	P	127.4547	P	0.2615	P
hsa-miR-3196_st	975.9194	P	2004.934	P	0.4868	P
hsa-miR-3197_st	5.765062	A	16.53351	P	0.3487	P
hsa-miR-31_st	1007.72	P	2057.169	P	0.4899	P
hsa-miR-331-3p_st	3.451929	A	7.873329	P	0.4384	P
hsa-miR-34a_st	73.11859	P	191.0961	P	0.3826	P
hsa-miR-34c-3p_st	16.26691	P	43.60644	P	0.373	P
hsa-miR-34c-5p_st	2.90753	A	16.55114	P	0.1757	P
hsa-miR-3615_st	5.166142	A	12.27675	P	0.4208	P
hsa-miR-3622a-5p_st	3.535996	P	7.634859	P	0.4631	P
hsa-miR-3663-3p_st	57.63831	P	154.0791	P	0.3741	P
hsa-miR-409-5p_st	3.618925	P	8.895913	P	0.4068	P
hsa-miR-4286_st	4.582221	P	10.22683	P	0.4481	P
hsa-miR-4317_st	5.563763	P	11.33158	P	0.491	P
hsa-miR-4321_st	2.554576	A	5.678205	P	0.4499	P
hsa-miR-4465_st	2.228638	A	4.864153	P	0.4582	P
hsa-miR-4486_st	13.48293	P	48.83792	P	0.2761	P
hsa-miR-4497_st	7028.993	P	26263.59	P	0.2676	P
hsa-miR-4498_st	4.042892	A	8.54569	P	0.4731	P
hsa-miR-4532_st	85.71832	P	273.8879	P	0.313	P
hsa-miR-4634_st	12.24729	P	36.28807	P	0.3375	P
hsa-miR-4665-5p_st	19.76414	P	40.56366	P	0.4872	P
hsa-miR-4674_st	95.18689	P	244.3705	P	0.3895	P
hsa-miR-4695-3p_st	3.23268	P	8.992185	P	0.3595	P
hsa-miR-4721_st	18.05624	P	48.95474	P	0.3688	P
hsa-miR-4730_st	4.221957	P	12.20957	P	0.3458	P
hsa-miR-4734_st	255.0684	P	531.2877	P	0.4801	P
hsa-miR-4745-5p_st	334.6163	P	670.5026	P	0.4991	P
hsa-miR-484_st	2.283751	A	5.807216	P	0.3933	P
hsa-miR-887_st	9.241057	P	20.14201	P	0.4588	P
hsa-miR-943_st	3.195108	P	7.02274	P	0.455	P
hsa-miR-99a_st	42.46676	P	113.5987	P	0.3738	P

Table 4. Different expression of miRNAs between sample GCs2 and Con 2

ProbeSetID	GCs 2	GCs 2.call	Con 2	Con 2.call	Ratio	Ratio.call
52 miRNAs overexpression in GCs 2 MSCs						
hsa-miR-1228-star_st	209.8376	P	99.4548	P	2.1099	P
hsa-miR-1231_st	9.963836	P	3.018058	A	3.3014	P
hsa-miR-1909_st	16.48049	P	7.495262	P	2.1988	P
hsa-miR-1915_st	1456.104	P	615.4554	P	2.3659	P
hsa-miR-210_st	605.955	P	278.2881	P	2.1774	P
hsa-miR-214_st	5680.75	P	2634.234	P	2.1565	P
hsa-miR-2861_st	1044.837	P	440.3198	P	2.3729	P
hsa-miR-3124-5p_st	6.097641	P	2.813545	A	2.1672	P
hsa-miR-3180-3p_st	10.76487	P	4.992606	P	2.1562	P
hsa-miR-3180_st	11.8036	P	4.123191	A	2.8627	P
hsa-miR-3185_st	192.3462	P	69.58349	P	2.7643	P
hsa-miR-3188_st	11.78586	P	2.559243	A	4.6052	P
hsa-miR-3195_st	41.80313	P	4.995592	P	8.368	P
hsa-miR-3196_st	996.6777	P	421.9544	P	2.3621	P
hsa-miR-34c-3p_st	26.72535	P	6.503896	P	4.1091	P
hsa-miR-3615_st	17.96207	P	6.923045	P	2.5945	P
hsa-miR-3621_st	39.37968	P	16.4656	P	2.3916	P
hsa-miR-3648_st	22.29397	P	10.77479	P	2.0691	P
hsa-miR-3663-3p_st	74.37399	P	34.54029	P	2.1533	P
hsa-miR-3665_st	6731.312	P	2774.795	P	2.4259	P
hsa-miR-3940-5p_st	506.6394	P	213.9685	P	2.3678	P
hsa-miR-3960_st	5808.405	P	2591.711	P	2.2411	P
hsa-miR-4281_st	224.4868	P	69.12266	P	3.2477	P
hsa-miR-4324_st	15.20699	P	7.268849	P	2.0921	P
hsa-miR-4436b-5p_st	22.22273	P	5.895072	P	3.7697	P
hsa-miR-4466_st	1721.334	P	715.4944	P	2.4058	P
hsa-miR-4467_st	25.61971	P	9.811595	P	2.6112	P
hsa-miR-4486_st	60.03091	P	11.10027	P	5.4081	P
hsa-miR-4497_st	8902.426	P	2573.851	P	3.4588	P
hsa-miR-4505_st	84.44164	P	25.57227	P	3.3021	P
hsa-miR-4507_st	55.60133	P	21.38451	P	2.6001	P
hsa-miR-4516_st	1287.78	P	576.8301	P	2.2325	P
hsa-miR-4532_st	124.5749	P	46.59902	P	2.6733	P
hsa-miR-4634_st	37.47656	P	5.174047	A	7.2432	P
hsa-miR-4649-5p_st	30.64105	P	14.42561	P	2.1241	P
hsa-miR-4651_st	126.5477	P	46.81691	P	2.703	P
hsa-miR-4674_st	237.3726	P	105.1655	P	2.2571	P
hsa-miR-4689_st	40.34288	P	18.77866	P	2.1483	P
hsa-miR-4695-5p_st	54.45475	P	21.09485	P	2.5814	P

hsa-miR-4707-5p_st	249.3134	P	80.46018	P	3.0986	P
hsa-miR-4721_st	29.12842	P	7.693567	P	3.7861	P
hsa-miR-4734_st	310.225	P	114.2007	P	2.7165	P
hsa-miR-4741_st	78.20836	P	30.63719	P	2.5527	P
hsa-miR-4745-5p_st	499.0054	P	241.2746	P	2.0682	P
hsa-miR-4758-5p_st	39.37746	P	18.78168	P	2.0966	P
hsa-miR-4763-3p_st	441.8569	P	176.2371	P	2.5072	P
hsa-miR-4800-3p_st	8.727445	P	3.074457	P	2.8387	P
hsa-miR-483-5p_st	15.14517	P	6.621949	P	2.2871	P
hsa-miR-485-5p_st	21.79527	P	9.208059	P	2.367	P
hsa-miR-491-5p_st	42.57394	P	16.30896	P	2.6105	P
hsa-miR-708_st	292.4446	P	122.4945	P	2.3874	P
hsa-miR-762_st	631.2592	P	238.0936	P	2.6513	P
35 miRNAs underexpression in GCs 2 MSCs						
hsa-miR-106b-star_st	36.45475	P	113.9353	P	0.32	P
hsa-miR-106b_st	128.9556	P	261.4259	P	0.4933	P
hsa-miR-1246_st	17.16216	P	39.52103	P	0.4343	P
hsa-miR-17-star_st	2.544924	A	7.100518	P	0.3584	P
hsa-miR-181d_st	2.173367	P	5.54943	P	0.3916	P
hsa-miR-1972_st	3.392031	A	20.15873	P	0.1683	P
hsa-miR-20b_st	6.378437	P	13.41755	P	0.4754	P
hsa-miR-212_st	7.514286	P	15.20402	P	0.4942	P
hsa-miR-25-star_st	12.36935	P	30.95476	P	0.3996	P
hsa-miR-25_st	9.053917	P	33.29024	P	0.272	P
hsa-miR-29b-1-star_st	10.73585	P	41.65932	P	0.2577	P
hsa-miR-29b_st	2.845495	P	5.839915	P	0.4872	P
hsa-miR-30a_st	6.257889	P	16.1631	P	0.3872	P
hsa-miR-3197_st	9.293846	A	34.98965	P	0.2656	P
hsa-miR-3613-5p_st	5.168764	P	12.79643	P	0.4039	P
hsa-miR-3651_st	22.4279	P	45.62141	P	0.4916	P
hsa-miR-378-star_st	2.8011	A	7.060409	P	0.3967	P
hsa-miR-378c_st	7.98418	P	19.06822	P	0.4187	P
hsa-miR-382_st	24.13431	P	55.63332	P	0.4338	P
hsa-miR-3972_st	4.934426	A	13.37248	P	0.369	P
hsa-miR-4304_st	12.38962	P	35.78807	P	0.3462	P
hsa-miR-4443_st	61.11358	P	135.1194	P	0.4523	P
hsa-miR-4487_st	5.015908	P	12.25874	P	0.4092	P
hsa-miR-486-3p_st	3.731085	P	9.232656	P	0.4041	P
hsa-miR-487b_st	25.42496	P	62.64977	P	0.4058	P
hsa-miR-497_st	2.343576	A	5.085016	P	0.4609	P
hsa-miR-505-star_st	14.99481	P	37.00866	P	0.4052	P
hsa-miR-551b-star_st	2.747215	A	5.542988	P	0.4956	P
hsa-miR-625_st	7.300247	P	15.19625	P	0.4804	P
hsa-miR-629-star_st	3.287994	P	8.943404	P	0.3676	P

hsa-miR-652_st	14.10784	P	31.74179	P	0.4445	P
hsa-miR-720_st	9.333636	P	21.96447	P	0.4249	P
hsa-miR-766_st	2.386085	A	5.80447	P	0.4111	P
hsa-miR-92a-1_st	5.019529	P	13.17309	P	0.381	P
hsa-miR-93-star_st	12.68089	P	27.21088	P	0.466	P

Table 5. Different expression of miRNAs between sample GCs3 and Con 3

ProbeSetID	GCs 3	GCs 3.call	Con 3	Con 3.call	Ratio	Ratio.call
21 miRNAs overexpression in GCs 3 MSCs						
hsa-miR-127-3p_st	1049.455	P	505.2288	P	2.0772	P
hsa-miR-127-5p_st	14.44282	P	2.746511	A	5.2586	P
hsa-miR-193a-3p_st	14.49871	P	7.061127	P	2.0533	P
hsa-miR-299-3p_st	13.2114	P	4.505029	P	2.9326	P
hsa-miR-3180-3p_st	11.35838	P	4.723107	A	2.4049	P
hsa-miR-335_st	3.776224	P	1.712869	A	2.2046	P
hsa-miR-3609_st	7.699952	P	3.440807	P	2.2378	P
hsa-miR-3613-5p_st	22.05914	P	4.51425	P	4.8866	P
hsa-miR-409-3p_st	189.09	P	88.53043	P	2.1359	P
hsa-miR-409-5p_st	14.70197	P	7.32889	P	2.006	P
hsa-miR-431-star_st	6.134541	P	2.6926	A	2.2783	P
hsa-miR-431_st	44.1214	P	18.47458	P	2.3882	P
hsa-miR-4484_st	66.98244	P	27.87196	P	2.4032	P
hsa-miR-483-5p_st	48.34686	P	12.2995	P	3.9308	P
hsa-miR-493_st	104.5967	P	33.57061	P	3.1157	P
hsa-miR-654-5p_st	37.8026	P	13.03812	P	2.8994	P
hsa-miR-665_st	44.51828	P	12.25253	P	3.6334	P
hsa-miR-708_st	190.984	P	89.01905	P	2.1454	P
hsa-miR-720_st	30.69121	P	8.190856	P	3.747	P
hsa-miR-874_st	65.52345	P	28.7247	P	2.2811	P
hsa-miR-933_st	10.24794	P	5.091783	A	2.0126	P
39 miRNAs underexpression in GCs 3 MSCs						
hsa-let-7b-star_st	2.003789	A	4.153776	P	0.4824	P
hsa-let-7f_st	76.197	P	212.9013	P	0.3579	P
hsa-miR-106b_st	75.20846	P	151.0142	P	0.498	P
hsa-miR-1184_st	8.815897	P	34.45475	P	0.2559	P
hsa-miR-138-1-star_st	91.31638	P	228.487	P	0.3997	P
hsa-miR-148a_st	3.017106	P	9.405887	P	0.3208	P
hsa-miR-1825_st	3.877048	P	12.37371	P	0.3133	P
hsa-miR-1972_st	3.070032	A	8.893475	P	0.3452	P
hsa-miR-19a_st	2.437328	P	5.071035	P	0.4806	P
hsa-miR-19b_st	4.497584	P	16.85729	P	0.2668	P
hsa-miR-20b_st	2.950442	P	10.64879	P	0.2771	P
hsa-miR-21_st	82.66455	P	406.4422	P	0.2034	P
hsa-miR-224_st	2.283751	P	5.221722	P	0.4374	P
hsa-miR-25_st	4.947055	P	11.19656	P	0.4418	P
hsa-miR-28-3p_st	10.34019	P	34.1643	P	0.3027	P
hsa-miR-30a_st	4.615528	P	9.387289	P	0.4917	P
hsa-miR-30c_st	4.656307	P	48.23899	P	0.0965	P

hsa-miR-3162-5p_st	6.1735	P	15.77745	P	0.3913	P
hsa-miR-3177-3p_st	2.567162	A	8.97224	P	0.2861	P
hsa-miR-3197_st	5.087512	A	14.04524	P	0.3622	P
hsa-miR-3679-5p_st	7.416359	P	18.00422	P	0.4119	P
hsa-miR-3911_st	13.09589	P	40.29239	P	0.325	P
hsa-miR-3935_st	1.836445	A	4.140778	P	0.4435	P
hsa-miR-4286_st	3.776224	P	7.606172	P	0.4965	P
hsa-miR-4304_st	3.241375	P	33.76715	P	0.096	P
hsa-miR-4436b-5p_st	9.009513	P	23.04123	P	0.391	P
hsa-miR-4443_st	49.56123	P	107.9548	P	0.4591	P
hsa-miR-4486_st	6.066138	P	25.76669	P	0.2354	P
hsa-miR-4487_st	5.722239	P	11.66737	P	0.4904	P
hsa-miR-4655-5p_st	4.378256	A	10.03739	P	0.4362	P
hsa-miR-4656_st	3.579862	A	7.767654	P	0.4609	P
hsa-miR-4669_st	21.17814	P	62.6009	P	0.3383	P
hsa-miR-4701-3p_st	5.1324	P	20.37171	P	0.2519	P
hsa-miR-4788_st	2.06756	A	5.076513	P	0.4073	P
hsa-miR-486-3p_st	6.994882	P	71.55325	P	0.0978	P
hsa-miR-486-5p_st	11.18014	P	72.9583	P	0.1532	P
hsa-miR-542-5p_st	3.209794	P	9.600534	P	0.3343	P
hsa-miR-885-5p_st	2.047729	A	5.027822	P	0.4073	P
hsa-miR-92a-1_st	5.485594	P	20.04762	P	0.2736	P

Table 6. Different expression of miRNAs in at least 2 pairs

ProbeSetID	GCs1 vs Con1	Ratio. call	GCs2 vs Con2	Ratio. call	GCs3 vs Con3	Ratio. call
hsa-miR-3180-3p_st	0.355	P	2.1562	P	2.4049	P
hsa-miR-483-5p_st	1.5296	P	2.2871	P	3.9308	P
hsa-miR-708_st	0.9936	P	2.3874	P	2.1454	P
hsa-miR-3197_st	0.3487	P	0.2656	P	0.3622	P
hsa-miR-4304_st	4.859	P	0.3462	P	0.096	P
hsa-miR-4443_st	2.1086	P	0.4523	P	0.4591	P
hsa-miR-4486_st	0.2761	P	5.4081	P	0.2354	P
hsa-miR-4487_st	3.2415	P	0.4092	P	0.4904	P
hsa-miR-92a-1_st	2.0214	P	0.381	P	0.2736	P
hsa-miR-1972_st	1.0762	A	0.1683	P	0.3452	P
hsa-miR-20b_st	0.5416	P	0.4754	P	0.2771	P
hsa-miR-25_st	0.5211	P	0.272	P	0.4418	P
hsa-miR-4286_st	0.4481	P	0.8079	P	0.4965	P
hsa-miR-486-3p_st	0.975	P	0.4041	P	0.0978	P
hsa-miR-106b_st	1.0523	P	0.4933	P	0.489	P
hsa-miR-30a_st	1.0785	P	0.3872	P	0.4917	P

Table 7. Target gene prediction of hsa-mir-708 by online software

MicroRNA	Target Gene	Prediction Program										SUM
		DIANAmt	miRanda	miRDB	miRWalk	RNAhybrid	PICTAR4	PICTAR5	PITA	RNA22	Targetscan	
hsa-mir-708	SMAD3	√	√					√			√	4
hsa-mir-708	SMAD4	√	√		√			√			√	5

Table 8. Target genes of hsa-mir-708 (SUM≥4)

MicroRNA	Gene	DIANAmT	miRanda	miRDB	miRWalk	RNAhybrid	PICTAR4	PICTAR5	PITA	RNA22	Targetscan	SUM
hsa-miR-708	AAK1	0	1	1	1	0	0	1	0	0	1	5
hsa-miR-708	AATK	1	1	0	1	0	0	1	0	0	1	5
hsa-miR-708	ABBA-1	1	1	0	1	0	0	1	0	0	1	5
hsa-miR-708	ABCA13	1	1	0	1	0	0	1	0	0	1	5
hsa-miR-708	ABCA3	1	1	0	1	0	0	1	0	0	0	4
hsa-miR-708	ABCA5	1	0	1	1	0	0	1	0	0	1	5
hsa-miR-708	ABCB8	1	1	0	0	0	0	1	0	0	1	4
hsa-miR-708	ABHD13	1	1	0	1	0	0	1	0	0	1	5
hsa-miR-708	ABHD2	1	0	0	1	0	0	1	0	0	1	4
hsa-miR-708	ABHD4	1	1	0	1	0	0	1	0	0	0	4
hsa-miR-708	ABI2	1	1	0	1	0	0	1	0	0	1	5
hsa-miR-708	ABLIM1	1	0	0	1	0	0	1	0	0	1	4
hsa-miR-708	ABLIM3	1	1	0	1	0	0	1	0	0	1	5
hsa-miR-708	ABTB2	1	1	0	1	0	0	1	0	0	0	4
hsa-miR-708	ACACA	1	1	0	1	0	0	0	0	0	1	4
hsa-miR-708	ACACB	0	1	0	1	0	0	1	0	0	1	4
hsa-miR-708	ACAD8	1	1	0	1	0	0	1	0	0	1	5
hsa-miR-708	ACHE	1	1	0	1	0	0	1	0	0	0	4
hsa-miR-708	ACOX3	1	1	0	1	0	0	0	0	0	1	4
hsa-miR-708	ACSF2	1	1	0	1	0	0	1	0	0	1	5
hsa-miR-708	ACTR2	1	1	0	1	0	0	1	0	0	0	4
hsa-miR-708	ACVR1B	1	1	0	1	0	0	1	0	0	1	5
hsa-miR-708	ADAM11	1	1	0	1	0	0	1	0	0	1	5
hsa-miR-708	ADAM19	1	1	0	1	0	0	1	0	0	1	5
hsa-miR-708	ADAM33	1	1	0	1	0	0	1	0	0	1	5
hsa-miR-708	ADAMTS14	1	1	0	1	0	0	1	0	0	1	5
hsa-miR-708	ADAMTSL1	1	1	0	1	0	0	1	0	0	0	4
hsa-miR-708	ADCY1	1	1	0	1	0	0	1	0	0	1	5
hsa-miR-708	ADCY2	1	1	0	0	0	0	1	0	0	1	4
hsa-miR-708	ADD2	1	1	0	1	0	0	1	0	0	1	5
hsa-miR-708	ADIPOQ	1	1	0	1	0	0	1	0	0	0	4
hsa-miR-708	ADM2	1	1	0	1	0	0	1	0	0	1	5
hsa-miR-708	ADRBK2	0	1	0	1	0	0	1	0	0	1	4
hsa-miR-708	AFAP1	1	1	0	1	0	0	0	0	0	1	4
hsa-miR-708	AFF1	1	1	0	1	0	0	1	0	0	1	5
hsa-miR-708	AFF4	1	1	1	1	0	0	1	0	0	1	6
hsa-miR-708	AGMAT	1	1	0	1	0	0	1	0	0	1	5
hsa-miR-708	AGPAT3	1	1	1	1	0	0	1	0	0	1	6
hsa-miR-708	AGPAT4	1	1	0	1	0	0	1	0	0	1	5

hsa-miR-708	AGRN	1	1	0	1	0	0	1	0	0	1	5
hsa-miR-708	AGTRL1	0	1	0	1	0	0	1	0	0	1	4
hsa-miR-708	AHDC1	1	1	0	1	0	0	1	0	0	1	5
hsa-miR-708	AHRR	1	1	0	1	0	0	1	0	0	1	5
hsa-miR-708	AIFM2	1	1	0	1	0	0	1	0	0	1	5
hsa-miR-708	AK2	1	0	0	1	0	0	1	0	0	1	4
hsa-miR-708	AKAP11	1	1	0	1	0	0	1	0	0	1	5
hsa-miR-708	AKT2	1	1	0	0	0	0	1	0	0	1	4
hsa-miR-708	ALDH1A2	1	1	0	1	0	0	1	0	0	1	5
hsa-miR-708	ALDH3A2	1	1	0	1	0	0	1	0	0	1	5
hsa-miR-708	ALDH5A1	1	1	0	1	0	0	1	0	0	1	5
hsa-miR-708	ALG2	1	1	0	1	0	0	1	0	0	1	5
hsa-miR-708	ALG9	1	1	0	1	0	0	0	0	0	1	4
hsa-miR-708	ALKBH1	1	1	0	1	0	0	1	0	0	0	4
hsa-miR-708	ALKBH5	1	1	0	1	0	0	1	0	0	0	4
hsa-miR-708	ALPK3	1	1	0	1	0	0	1	0	0	1	5
hsa-miR-708	ALPL	1	1	0	1	0	0	1	0	0	1	5
hsa-miR-708	ALPP	1	1	0	1	0	0	1	0	0	1	5
hsa-miR-708	AMIGO2	1	1	0	0	0	0	1	0	0	1	4
hsa-miR-708	AMMECR1	1	1	0	1	0	0	1	0	0	1	5
hsa-miR-708	AMMECR1L	1	1	0	1	0	0	1	0	0	1	5
hsa-miR-708	AMOTL1	1	1	0	1	0	0	1	0	0	1	5
hsa-miR-708	AMOTL2	1	1	0	0	0	0	1	0	0	1	4
hsa-miR-708	AMPD2	1	1	0	1	0	0	1	0	0	0	4
hsa-miR-708	AMPH	1	1	1	1	0	0	1	0	0	1	6
hsa-miR-708	AMZ1	1	1	0	1	0	0	1	0	0	1	5
hsa-miR-708	ANK1	1	1	0	0	0	0	1	0	0	1	4
hsa-miR-708	ANK2	1	1	0	1	0	0	1	0	0	1	5
hsa-miR-708	ANKRD13B	1	1	0	1	0	0	1	0	0	1	5
hsa-miR-708	ANKRD23	1	1	0	1	0	0	1	0	0	1	5
hsa-miR-708	ANKRD32	1	1	0	1	0	0	1	0	0	1	5
hsa-miR-708	ANKRD37	1	1	0	1	0	0	1	0	0	1	5
hsa-miR-708	ANKRD45	1	1	0	1	0	0	1	0	0	1	5
hsa-miR-708	ANKS6	1	1	0	1	0	0	1	0	0	1	5
hsa-miR-708	ANXA9	1	1	0	1	0	0	1	0	0	1	5
hsa-miR-708	AOF2	1	0	0	1	0	0	1	0	0	1	4
hsa-miR-708	AP1B1	1	1	0	1	0	0	1	0	0	1	5
hsa-miR-708	AP2A2	1	1	0	1	0	0	1	0	0	1	5
hsa-miR-708	AP2B1	1	1	1	1	0	0	1	0	0	1	6
hsa-miR-708	APAF1	1	1	0	1	0	0	1	0	0	1	5
hsa-miR-708	APBB2	1	1	0	0	0	0	1	0	0	1	4
hsa-miR-708	APEX2	1	1	0	1	0	0	1	0	0	1	5
hsa-miR-708	APOA1BP	0	1	0	1	0	0	1	0	0	1	4
hsa-miR-708	APOA2	1	1	0	1	0	0	1	0	0	1	5

hsa-miR-708	APOA5	1	1	0	1	0	0	1	0	0	1	5
hsa-miR-708	APOBEC4	1	1	0	1	0	0	1	0	0	0	4
hsa-miR-708	APOC3	1	1	0	1	0	0	1	0	0	1	5
hsa-miR-708	APOOL	1	1	0	1	0	0	1	0	0	0	4
hsa-miR-708	APPBP2	1	1	0	0	0	0	1	0	0	1	4
hsa-miR-708	AQP6	1	1	0	1	0	0	1	0	0	1	5
hsa-miR-708	ARAF	1	1	0	1	0	0	1	0	0	1	5
hsa-miR-708	ARC	1	1	0	1	0	0	1	0	0	1	5
hsa-miR-708	ARCN1	1	1	0	1	0	0	1	0	0	0	4
hsa-miR-708	ARF5	1	1	0	1	0	0	1	0	0	1	5
hsa-miR-708	ARFGAP2	1	1	0	1	0	0	1	0	0	1	5
hsa-miR-708	ARFIP2	1	1	0	1	0	0	1	0	0	0	4
hsa-miR-708	ARHGAP1	1	1	0	1	0	0	1	0	0	0	4
hsa-miR-708	ARHGAP17	1	1	0	1	0	0	1	0	0	0	4
hsa-miR-708	ARHGAP19	1	1	0	1	0	0	1	0	0	1	5
hsa-miR-708	ARHGAP24	1	1	0	1	0	0	1	0	0	1	5
hsa-miR-708	ARHGEF9	1	1	0	0	0	0	1	0	0	1	4
hsa-miR-708	ARID2	1	1	0	1	0	0	1	0	0	0	4
hsa-miR-708	ARL2	1	1	0	1	0	0	1	0	0	0	4
hsa-miR-708	ARL3	1	1	0	1	0	0	1	0	0	0	4
hsa-miR-708	ARMC5	1	1	0	1	0	0	1	0	0	0	4
hsa-miR-708	ARMC6	1	1	0	1	0	0	1	0	0	0	4
hsa-miR-708	ARNT2	1	1	0	1	0	0	1	0	0	1	5
hsa-miR-708	ARPP-19	1	1	0	1	0	0	1	0	0	1	5
hsa-miR-708	ARPP-21	1	1	0	1	0	0	0	0	0	1	4
hsa-miR-708	ARRDC1	1	1	0	1	0	0	1	0	0	1	5
hsa-miR-708	ARRDC2	1	0	0	1	0	0	1	0	0	1	4
hsa-miR-708	ARSJ	1	1	0	1	0	0	1	0	0	0	4
hsa-miR-708	AS3MT	1	1	1	1	0	0	1	0	0	1	6
hsa-miR-708	ASAHL	1	1	0	1	0	0	1	0	0	0	4
hsa-miR-708	ASB1	1	1	0	1	0	0	1	0	0	1	5
hsa-miR-708	ASB13	1	1	0	1	0	0	1	0	0	0	4
hsa-miR-708	ASB15	1	1	0	1	0	0	1	0	0	1	5
hsa-miR-708	ASB5	1	1	1	1	0	0	1	0	0	1	6
hsa-miR-708	ASB8	1	1	0	0	0	0	1	0	0	1	4
hsa-miR-708	ASCC3	1	1	0	1	0	0	0	0	0	1	4
hsa-miR-708	ASPA	1	1	1	1	0	0	1	0	0	1	6
hsa-miR-708	ASXL1	1	1	0	1	0	0	1	0	0	0	4
hsa-miR-708	ATAD2B	0	1	1	1	0	0	1	0	0	1	5
hsa-miR-708	ATCAY	0	1	0	1	0	0	1	0	0	1	4
hsa-miR-708	ATHL1	1	1	0	1	0	0	1	0	0	1	5
hsa-miR-708	ATMIN	1	1	0	1	0	0	1	0	0	0	4
hsa-miR-708	ATOH8	1	1	0	1	0	0	1	0	0	0	4
hsa-miR-708	ATP11A	1	1	0	1	0	0	1	0	0	1	5

hsa-miR-708	ATP2B3	1	1	0	1	0	0	1	0	0	1	5
hsa-miR-708	ATP5D	1	1	0	1	0	0	1	0	0	1	5
hsa-miR-708	ATP5SL	1	1	0	1	0	0	1	0	0	1	5
hsa-miR-708	ATP6V1A	1	1	1	1	0	0	1	0	0	1	6
hsa-miR-708	ATP6V1F	1	1	0	1	0	0	1	0	0	0	4
hsa-miR-708	ATP7A	1	1	0	1	0	0	1	0	0	0	4
hsa-miR-708	ATP8A1	1	1	0	1	0	0	1	0	0	1	5
hsa-miR-708	ATP9A	1	1	0	1	0	0	1	0	0	1	5
hsa-miR-708	ATPAF1	1	1	0	1	0	0	1	0	0	1	5
hsa-miR-708	ATRN	1	1	0	1	0	0	1	0	0	1	5
hsa-miR-708	ATRNL1	1	1	1	1	0	0	1	0	0	1	6
hsa-miR-708	ATXN1	1	0	0	1	0	0	1	0	0	1	4
hsa-miR-708	ATXN2L	1	1	0	1	0	0	1	0	0	0	4
hsa-miR-708	ATXN7L1	1	1	0	1	0	0	0	0	0	1	4
hsa-miR-708	AVPR1A	1	1	0	1	0	0	1	0	0	1	5
hsa-miR-708	AXIN2	1	1	0	1	0	0	1	0	0	0	4
hsa-miR-708	B3GNT3	1	1	1	0	0	0	1	0	0	1	5
hsa-miR-708	B4GALNT1	1	1	0	1	0	0	1	0	0	1	5
hsa-miR-708	B4GALNT3	1	1	0	1	0	0	1	0	0	1	5
hsa-miR-708	B4GALT3	1	1	0	1	0	0	1	0	0	1	5
hsa-miR-708	B4GALT4	1	1	0	1	0	0	0	0	0	1	4
hsa-miR-708	BACE1	1	1	0	1	0	0	1	0	0	1	5
hsa-miR-708	BAG1	0	1	1	1	0	0	1	0	0	1	5
hsa-miR-708	BAI1	1	1	0	1	0	0	1	0	0	1	5
hsa-miR-708	BAZ1B	1	1	0	1	0	0	1	0	0	1	5
hsa-miR-708	BBC3	1	1	0	1	0	0	1	0	0	0	4
hsa-miR-708	BBS1	1	1	0	1	0	0	1	0	0	1	5
hsa-miR-708	BBS10	1	1	1	1	0	0	1	0	0	1	6
hsa-miR-708	BBS5	1	1	0	1	0	0	1	0	0	1	5
hsa-miR-708	BCAM	1	1	0	1	0	0	1	0	0	1	5
hsa-miR-708	BCAT1	1	1	0	1	0	0	1	0	0	1	5
hsa-miR-708	BCL11B	1	1	0	1	0	0	1	0	0	0	4
hsa-miR-708	BCL2L11	1	1	0	1	0	0	1	0	0	1	5
hsa-miR-708	BCL2L15	1	1	0	1	0	0	1	0	0	1	5
hsa-miR-708	BCL7A	1	1	0	1	0	0	1	0	0	1	5
hsa-miR-708	BCR	1	1	0	1	0	0	1	0	0	1	5
hsa-miR-708	BDKRB2	1	1	0	1	0	0	1	0	0	1	5
hsa-miR-708	BEGAIN	1	0	1	1	0	0	1	0	0	1	5
hsa-miR-708	BET1	0	1	0	1	0	0	1	0	0	1	4
hsa-miR-708	BHLHB2	1	1	0	1	0	0	1	0	0	1	5
hsa-miR-708	BHMT2	1	1	0	1	0	0	1	0	0	1	5
hsa-miR-708	BICD2	1	1	0	1	0	0	1	0	0	0	4
hsa-miR-708	BIRC5	1	1	0	1	0	0	1	0	0	0	4
hsa-miR-708	BIRC6	1	1	0	1	0	0	1	0	0	0	4

hsa-miR-708	BIVM	1	0	0	1	0	0	1	0	0	1	4
hsa-miR-708	BLMH	1	1	0	1	0	0	1	0	0	1	5
hsa-miR-708	BMF	1	1	0	1	0	0	1	0	0	1	5
hsa-miR-708	BNIP1	1	1	0	1	0	0	0	0	0	1	4
hsa-miR-708	BRCA1	1	1	0	1	0	0	0	0	0	1	4
hsa-miR-708	BRD3	1	1	0	1	0	0	1	0	0	1	5
hsa-miR-708	BRF1	1	1	0	0	0	0	1	0	0	1	4
hsa-miR-708	BRPF3	1	1	0	1	0	0	1	0	0	1	5
hsa-miR-708	BRSK2	1	1	0	1	0	0	1	0	0	1	5
hsa-miR-708	BSN	1	1	0	1	0	0	1	0	0	1	5
hsa-miR-708	BTBD14B	1	1	0	1	0	0	1	0	0	1	5
hsa-miR-708	BTBD7	1	1	0	1	0	0	1	0	0	1	5
hsa-miR-708	BTLA	1	1	0	1	0	0	1	0	0	1	5
hsa-miR-708	BTRC	1	1	1	1	0	0	1	0	0	1	6
hsa-miR-708	BZRAP1	1	1	0	1	0	0	1	0	0	1	5
hsa-miR-708	C10orf118	1	1	0	1	0	0	1	0	0	1	5
hsa-miR-708	C10orf2	1	1	0	1	0	0	1	0	0	1	5
hsa-miR-708	C10orf25	1	1	0	0	0	0	1	0	0	1	4
hsa-miR-708	C10orf4	1	1	1	1	0	0	1	0	0	1	6
hsa-miR-708	C10orf46	1	1	0	1	0	0	1	0	0	0	4
hsa-miR-708	C10orf54	1	1	0	0	0	0	1	0	0	1	4
hsa-miR-708	C10orf56	1	1	0	1	0	0	1	0	0	0	4
hsa-miR-708	C10orf57	1	1	0	1	0	0	1	0	0	0	4
hsa-miR-708	C11orf56	1	1	0	1	0	0	1	0	0	0	4
hsa-miR-708	C11orf61	1	1	0	1	0	0	1	0	0	1	5
hsa-miR-708	C11orf79	1	1	0	1	0	0	1	0	0	0	4
hsa-miR-708	C12orf29	1	1	1	1	0	0	1	0	0	1	6
hsa-miR-708	C12orf53	1	1	0	1	0	0	1	0	0	1	5
hsa-miR-708	C13orf23	1	1	0	1	0	0	1	0	0	1	5
hsa-miR-708	C13orf30	1	1	0	1	0	0	1	0	0	1	5
hsa-miR-708	C14orf1	1	1	0	0	0	0	1	0	0	1	4
hsa-miR-708	C14orf101	1	1	0	1	0	0	1	0	0	1	5
hsa-miR-708	C14orf105	1	1	1	1	0	0	1	0	0	1	6
hsa-miR-708	C14orf108	1	1	1	1	0	0	1	0	0	1	6
hsa-miR-708	C14orf124	1	1	0	1	0	0	1	0	0	1	5
hsa-miR-708	C14orf130	1	1	0	1	0	0	1	0	0	1	5
hsa-miR-708	C14orf28	1	1	0	1	0	0	1	0	0	1	5
hsa-miR-708	C14orf4	0	1	0	1	0	0	1	0	0	1	4
hsa-miR-708	C14orf43	1	1	0	1	0	0	0	0	0	1	4
hsa-miR-708	C14orf83	1	1	0	1	0	0	1	0	0	1	5
hsa-miR-708	C15orf15	1	1	1	1	0	0	1	0	0	1	6
hsa-miR-708	C15orf17	1	1	0	1	0	0	1	0	0	1	5
hsa-miR-708	C15orf2	1	1	0	1	0	0	1	0	0	1	5
hsa-miR-708	C15orf52	1	1	0	1	0	0	1	0	0	1	5

hsa-miR-708	C16orf63	1	1	0	1	0	0	1	0	0	1	5
hsa-miR-708	C16orf72	1	1	0	1	0	0	1	0	0	1	5
hsa-miR-708	C16orf77	1	1	0	1	0	0	1	0	0	1	5
hsa-miR-708	C16orf78	1	1	0	1	0	0	1	0	0	1	5
hsa-miR-708	C17orf37	1	1	0	1	0	0	1	0	0	1	5
hsa-miR-708	C17orf56	1	1	0	1	0	0	1	0	0	0	4
hsa-miR-708	C17orf78	1	1	0	1	0	0	1	0	0	1	5
hsa-miR-708	C18orf1	1	0	0	1	0	0	1	0	0	1	4
hsa-miR-708	C18orf24	1	1	1	1	0	0	1	0	0	1	6
hsa-miR-708	C18orf25	1	1	0	1	0	0	1	0	0	1	5
hsa-miR-708	C19orf12	1	1	0	1	0	0	0	0	0	1	4
hsa-miR-708	C19orf21	1	1	0	1	0	0	1	0	0	1	5
hsa-miR-708	C19orf22	1	1	0	1	0	0	1	0	0	1	5
hsa-miR-708	C19orf25	1	1	0	1	0	0	1	0	0	1	5
hsa-miR-708	C19orf34	0	1	0	1	0	0	1	0	0	1	4
hsa-miR-708	C19orf47	1	1	0	1	0	0	1	0	0	1	5
hsa-miR-708	C19orf50	1	1	0	1	0	0	1	0	0	1	5
hsa-miR-708	C19orf60	1	0	0	1	0	0	1	0	0	1	4
hsa-miR-708	C1orf107	1	1	0	0	0	0	1	0	0	1	4
hsa-miR-708	C1orf113	1	0	0	1	0	0	1	0	0	1	4
hsa-miR-708	C1orf115	1	1	0	1	0	0	1	0	0	1	5
hsa-miR-708	C1orf119	0	1	1	1	0	0	0	0	0	1	4
hsa-miR-708	C1orf128	1	1	0	1	0	0	1	0	0	0	4
hsa-miR-708	C1orf144	1	1	0	1	0	0	0	0	0	1	4
hsa-miR-708	C1orf159	1	1	0	1	0	0	1	0	0	0	4
hsa-miR-708	C1orf161	1	1	0	1	0	0	1	0	0	0	4
hsa-miR-708	C1orf163	1	1	0	1	0	0	1	0	0	1	5
hsa-miR-708	C1orf177	1	1	0	1	0	0	1	0	0	0	4
hsa-miR-708	C1orf201	1	1	0	1	0	0	1	0	0	0	4
hsa-miR-708	C1orf212	0	1	0	1	0	0	1	0	0	1	4
hsa-miR-708	C1orf26	1	0	1	1	0	0	1	0	0	1	5
hsa-miR-708	C1orf27	1	1	1	1	0	0	1	0	0	1	6
hsa-miR-708	C1orf35	1	1	0	1	0	0	1	0	0	1	5
hsa-miR-708	C1orf61	1	1	0	1	0	0	1	0	0	1	5
hsa-miR-708	C1orf69	1	1	0	1	0	0	1	0	0	1	5
hsa-miR-708	C1orf90	1	1	0	1	0	0	1	0	0	1	5
hsa-miR-708	C1orf93	1	1	0	1	0	0	1	0	0	1	5
hsa-miR-708	C1orf95	0	1	0	1	0	0	1	0	0	1	4
hsa-miR-708	C1orf96	1	1	0	1	0	0	1	0	0	0	4
hsa-miR-708	C1QTNF1	1	1	0	1	0	0	1	0	0	1	5
hsa-miR-708	C1QTNF6	1	1	0	1	0	0	0	0	0	1	4
hsa-miR-708	C1RL	1	1	0	1	0	0	1	0	0	1	5
hsa-miR-708	C20orf11	1	1	0	1	0	0	1	0	0	1	5
hsa-miR-708	C20orf118	1	1	0	1	0	0	1	0	0	0	4

hsa-miR-708	C20orf121	1	1	0	1	0	0	0	0	0	1	4
hsa-miR-708	C20orf29	1	1	0	1	0	0	1	0	0	1	5
hsa-miR-708	C20orf3	1	1	0	1	0	0	1	0	0	1	5
hsa-miR-708	C20orf4	1	1	0	1	0	0	1	0	0	1	5
hsa-miR-708	C20orf59	1	1	0	1	0	0	1	0	0	1	5
hsa-miR-708	C20orf82	1	1	0	1	0	0	1	0	0	1	5
hsa-miR-708	C21orf29	1	1	0	1	0	0	1	0	0	0	4
hsa-miR-708	C22orf25	1	1	0	1	0	0	1	0	0	1	5
hsa-miR-708	C22orf29	1	1	0	1	0	0	1	0	0	1	5
hsa-miR-708	C22orf9	1	1	0	1	0	0	1	0	0	1	5
hsa-miR-708	C2orf18	1	1	0	1	0	0	1	0	0	1	5
hsa-miR-708	C2orf43	1	1	0	1	0	0	1	0	0	0	4
hsa-miR-708	C2orf60	1	1	0	1	0	0	1	0	0	1	5
hsa-miR-708	C3orf10	0	1	0	1	0	0	1	0	0	1	4
hsa-miR-708	C3orf17	1	1	1	1	0	0	1	0	0	1	6
hsa-miR-708	C3orf32	1	1	0	1	0	0	1	0	0	1	5
hsa-miR-708	C3orf52	1	1	0	1	0	0	1	0	0	1	5
hsa-miR-708	C3orf57	1	1	0	1	0	0	1	0	0	1	5
hsa-miR-708	C3orf62	1	1	0	1	0	0	1	0	0	0	4
hsa-miR-708	C5orf36	1	1	1	1	0	0	1	0	0	1	6
hsa-miR-708	C5orf4	0	1	0	1	0	0	1	0	0	1	4
hsa-miR-708	C6orf106	1	1	0	1	0	0	1	0	0	1	5
hsa-miR-708	C6orf151	1	1	0	1	0	0	1	0	0	1	5
hsa-miR-708	C6orf154	1	1	0	1	0	0	1	0	0	1	5
hsa-miR-708	C6orf223	1	1	0	1	0	0	1	0	0	1	5
hsa-miR-708	C6orf89	1	1	0	1	0	0	1	0	0	1	5
hsa-miR-708	C7orf20	0	1	0	1	0	0	1	0	0	1	4
hsa-miR-708	C7orf42	0	1	0	1	0	0	1	0	0	1	4
hsa-miR-708	C7orf50	1	1	0	1	0	0	0	0	0	1	4
hsa-miR-708	C7orf53	1	0	1	1	0	0	1	0	0	1	5
hsa-miR-708	C8orf40	1	0	0	1	0	0	1	0	0	1	4
hsa-miR-708	C8orf46	1	1	0	1	0	0	1	0	0	1	5
hsa-miR-708	C9orf100	1	1	0	1	0	0	1	0	0	1	5
hsa-miR-708	C9orf163	1	1	0	1	0	0	1	0	0	1	5
hsa-miR-708	C9orf19	1	1	0	1	0	0	1	0	0	1	5
hsa-miR-708	C9orf25	1	1	0	1	0	0	1	0	0	1	5
hsa-miR-708	C9orf3	1	1	0	1	0	0	1	0	0	1	5
hsa-miR-708	C9orf58	1	1	0	1	0	0	1	0	0	0	4
hsa-miR-708	C9orf7	1	1	0	1	0	0	1	0	0	1	5
hsa-miR-708	C9orf91	0	1	0	1	0	0	1	0	0	1	4
hsa-miR-708	CA13	1	1	0	1	0	0	1	0	0	1	5
hsa-miR-708	CA3	1	1	0	1	0	0	1	0	0	0	4
hsa-miR-708	CAB39L	1	1	0	1	0	0	1	0	0	1	5
hsa-miR-708	CABC1	1	1	0	1	0	0	1	0	0	0	4

hsa-miR-708	CABLES2	1	1	0	1	0	0	1	0	0	1	5
hsa-miR-708	CABP7	1	1	0	0	0	0	1	0	0	1	4
hsa-miR-708	CACNA1C	1	1	0	1	0	0	0	0	0	1	4
hsa-miR-708	CACNA1E	1	1	0	1	0	0	1	0	0	0	4
hsa-miR-708	CACNA1G	1	1	0	1	0	0	0	0	0	1	4
hsa-miR-708	CACNA1I	1	1	0	1	0	0	0	0	0	1	4
hsa-miR-708	CACNB1	1	1	0	1	0	0	1	0	0	0	4
hsa-miR-708	CACNG7	1	1	0	1	0	0	1	0	0	1	5
hsa-miR-708	CALB2	1	1	0	1	0	0	1	0	0	1	5
hsa-miR-708	CALCOCO2	1	1	0	1	0	0	1	0	0	1	5
hsa-miR-708	CALCR	1	1	0	1	0	0	1	0	0	0	4
hsa-miR-708	CALD1	1	1	0	1	0	0	1	0	0	1	5
hsa-miR-708	CALN1	1	0	0	1	0	0	1	0	0	1	4
hsa-miR-708	CALY	0	1	0	1	0	0	1	0	0	1	4
hsa-miR-708	CAMK2G	1	1	0	1	0	0	1	0	0	1	5
hsa-miR-708	CAMTA2	1	1	0	1	0	0	1	0	0	1	5
hsa-miR-708	CANT1	1	0	0	1	0	0	1	0	0	1	4
hsa-miR-708	CAPN1	1	1	0	1	0	0	1	0	0	0	4
hsa-miR-708	CAPN6	1	1	0	1	0	0	1	0	0	1	5
hsa-miR-708	CAPRIN1	1	1	0	1	0	0	1	0	0	1	5
hsa-miR-708	CARD14	1	1	0	1	0	0	1	0	0	0	4
hsa-miR-708	CASC3	1	1	0	1	0	0	1	0	0	1	5
hsa-miR-708	CASKIN1	1	1	0	0	0	0	1	0	0	1	4
hsa-miR-708	CASKIN2	1	1	0	0	0	0	1	0	0	1	4
hsa-miR-708	CASP2	1	1	1	1	0	0	1	0	0	1	6
hsa-miR-708	CASQ2	1	1	0	1	0	0	1	0	0	1	5
hsa-miR-708	CBFA2T2	1	0	0	1	0	0	1	0	0	1	4
hsa-miR-708	CBFB	1	1	1	1	0	0	1	0	0	1	6
hsa-miR-708	CBL	1	1	0	1	0	0	1	0	0	1	5
hsa-miR-708	CBLN2	1	1	0	1	0	0	1	0	0	1	5
hsa-miR-708	CBX2	1	1	0	1	0	0	1	0	0	1	5
hsa-miR-708	CBX7	1	1	0	1	0	0	1	0	0	1	5
hsa-miR-708	CCBE1	1	1	0	1	0	0	1	0	0	1	5
hsa-miR-708	CCDC109A	1	1	0	1	0	0	1	0	0	1	5
hsa-miR-708	CCDC113	1	1	1	1	0	0	1	0	0	1	6
hsa-miR-708	CCDC114	1	1	0	1	0	0	1	0	0	1	5
hsa-miR-708	CCDC117	1	1	0	1	0	0	1	0	0	1	5
hsa-miR-708	CCDC12	1	1	0	1	0	0	1	0	0	1	5
hsa-miR-708	CCDC121	1	0	0	1	0	0	1	0	0	1	4
hsa-miR-708	CCDC149	0	1	0	1	0	0	1	0	0	1	4
hsa-miR-708	CCDC25	1	1	0	1	0	0	1	0	0	1	5
hsa-miR-708	CCDC32	1	1	0	1	0	0	1	0	0	0	4
hsa-miR-708	CCDC4	1	1	0	1	0	0	1	0	0	1	5
hsa-miR-708	CCDC43	1	1	0	1	0	0	1	0	0	0	4

hsa-miR-708	CCDC6	1	1	0	1	0	0	1	0	0	1	5
hsa-miR-708	CCDC69	1	1	0	1	0	0	1	0	0	1	5
hsa-miR-708	CCDC78	1	1	0	1	0	0	0	0	0	1	4
hsa-miR-708	CCDC92	1	1	0	1	0	0	1	0	0	1	5
hsa-miR-708	CCDC93	1	1	0	1	0	0	1	0	0	1	5
hsa-miR-708	CCL19	1	1	0	1	0	0	1	0	0	1	5
hsa-miR-708	CCL5	1	1	0	1	0	0	1	0	0	0	4
hsa-miR-708	CCND2	1	1	0	1	0	0	1	0	0	1	5
hsa-miR-708	CCND3	1	1	0	1	0	0	1	0	0	1	5
hsa-miR-708	CCNE2	1	1	0	1	0	0	1	0	0	0	4
hsa-miR-708	CCNJL	1	1	0	1	0	0	1	0	0	0	4
hsa-miR-708	CCNL2	0	1	0	1	0	0	1	0	0	1	4
hsa-miR-708	CCR1	1	1	0	1	0	0	1	0	0	1	5
hsa-miR-708	CCRL2	1	1	0	1	0	0	0	0	0	1	4
hsa-miR-708	CD1A	1	1	0	1	0	0	1	0	0	1	5
hsa-miR-708	CD209	1	1	0	1	0	0	1	0	0	1	5
hsa-miR-708	CD276	1	1	0	1	0	0	1	0	0	1	5
hsa-miR-708	CD28	1	1	0	1	0	0	1	0	0	1	5
hsa-miR-708	CD2AP	1	1	0	1	0	0	1	0	0	1	5
hsa-miR-708	CD300LF	1	1	0	1	0	0	1	0	0	1	5
hsa-miR-708	CD300LG	1	1	0	1	0	0	1	0	0	0	4
hsa-miR-708	CD320	1	1	0	1	0	0	1	0	0	1	5
hsa-miR-708	CD33	1	1	0	1	0	0	1	0	0	1	5
hsa-miR-708	CD34	1	1	0	1	0	0	1	0	0	1	5
hsa-miR-708	CD38	1	1	0	1	0	0	1	0	0	1	5
hsa-miR-708	CD3G	1	1	0	1	0	0	1	0	0	1	5
hsa-miR-708	CD44	1	1	1	1	0	0	1	0	0	1	6
hsa-miR-708	CD47	1	1	0	1	0	0	1	0	0	1	5
hsa-miR-708	CD59	1	1	0	1	0	0	0	0	0	1	4
hsa-miR-708	CD97	1	1	0	0	0	0	1	0	0	1	4
hsa-miR-708	CD99L2	1	1	0	1	0	0	1	0	0	1	5
hsa-miR-708	CDC25B	1	1	0	1	0	0	1	0	0	0	4
hsa-miR-708	CDC34	1	1	0	1	0	0	1	0	0	0	4
hsa-miR-708	CDC40	1	1	0	1	0	0	1	0	0	0	4
hsa-miR-708	CDC42EP3	1	1	0	1	0	0	1	0	0	1	5
hsa-miR-708	CDC42EP4	1	1	0	1	0	0	1	0	0	1	5
hsa-miR-708	CDC42SE1	1	1	0	1	0	0	0	0	0	1	4
hsa-miR-708	CDC73	1	1	1	1	0	0	1	0	0	1	6
hsa-miR-708	CDCP1	1	1	0	1	0	0	1	0	0	1	5
hsa-miR-708	CDH18	1	1	0	1	0	0	1	0	0	1	5
hsa-miR-708	CDH5	1	1	0	1	0	0	1	0	0	1	5
hsa-miR-708	CDK5	1	1	0	1	0	0	1	0	0	0	4
hsa-miR-708	CDKN1A	1	1	0	1	0	0	0	0	0	1	4
hsa-miR-708	CDKN2B	1	1	0	1	0	0	1	0	0	1	5

hsa-miR-708	CDON	1	1	0	0	0	0	1	0	0	1	4
hsa-miR-708	CDRT4	0	1	0	1	0	0	1	0	0	1	4
hsa-miR-708	CDSN	1	1	1	1	0	0	1	0	0	1	6
hsa-miR-708	CDX1	1	1	0	1	0	0	1	0	0	1	5
hsa-miR-708	CEACAM6	1	1	0	0	0	0	1	0	0	1	4
hsa-miR-708	CEACAM7	1	1	0	1	0	0	1	0	0	1	5
hsa-miR-708	CEBPA	0	1	0	1	0	0	1	0	0	1	4
hsa-miR-708	CEBPG	1	1	0	1	0	0	1	0	0	0	4
hsa-miR-708	CENPB	1	1	0	1	0	0	1	0	0	0	4
hsa-miR-708	CENPF	1	1	1	1	0	0	1	0	0	1	6
hsa-miR-708	CENPN	1	1	0	1	0	0	1	0	0	1	5
hsa-miR-708	CENTB2	1	1	0	1	0	0	1	0	0	1	5
hsa-miR-708	CENTD3	1	1	0	1	0	0	1	0	0	1	5
hsa-miR-708	CENTG2	1	1	0	1	0	0	1	0	0	0	4
hsa-miR-708	CFL2	1	1	0	1	0	0	1	0	0	0	4
hsa-miR-708	CGNL1	1	1	0	1	0	0	1	0	0	1	5
hsa-miR-708	CHAD	1	1	0	1	0	0	1	0	0	1	5
hsa-miR-708	CHCHD4	1	1	0	1	0	0	0	0	0	1	4
hsa-miR-708	CHD3	1	1	0	0	0	0	1	0	0	1	4
hsa-miR-708	CHD5	1	1	0	1	0	0	1	0	0	1	5
hsa-miR-708	CHERP	1	1	0	1	0	0	1	0	0	1	5
hsa-miR-708	CHKA	1	1	0	1	0	0	1	0	0	0	4
hsa-miR-708	CHL1	1	1	0	0	0	0	1	0	0	1	4
hsa-miR-708	CHMP1A	1	1	0	1	0	0	1	0	0	1	5
hsa-miR-708	CHRAC1	1	1	0	1	0	0	1	0	0	1	5
hsa-miR-708	CHRM2	0	0	1	1	0	0	1	0	0	1	4
hsa-miR-708	CHRNA10	1	1	0	1	0	0	1	0	0	1	5
hsa-miR-708	CHRNA4	1	1	0	1	0	0	1	0	0	1	5
hsa-miR-708	CHRNB2	1	1	1	1	0	0	1	0	0	1	6
hsa-miR-708	CHST6	1	1	0	1	0	0	1	0	0	1	5
hsa-miR-708	CHSY3	0	1	0	1	0	0	1	0	0	1	4
hsa-miR-708	CIDEC	1	1	0	1	0	0	1	0	0	1	5
hsa-miR-708	CINP	1	1	0	1	0	0	1	0	0	0	4
hsa-miR-708	CIT	1	1	0	1	0	0	1	0	0	1	5
hsa-miR-708	CKAP2	1	1	0	1	0	0	1	0	0	1	5
hsa-miR-708	CLCN6	1	1	0	1	0	0	1	0	0	1	5
hsa-miR-708	CLDN1	1	1	0	1	0	0	1	0	0	0	4
hsa-miR-708	CLDN19	1	1	0	1	0	0	0	0	0	1	4
hsa-miR-708	CLDN6	1	1	0	1	0	0	1	0	0	1	5
hsa-miR-708	CLEC2B	1	1	0	1	0	0	1	0	0	1	5
hsa-miR-708	CLEC5A	1	1	0	0	0	0	1	0	0	1	4
hsa-miR-708	CLIP3	1	1	0	1	0	0	1	0	0	1	5
hsa-miR-708	CLN6	1	1	0	1	0	0	1	0	0	1	5
hsa-miR-708	CLN8	1	1	0	1	0	0	1	0	0	1	5

hsa-miR-708	CLNS1A	1	1	0	1	0	0	1	0	0	1	5
hsa-miR-708	CMIP	0	1	0	1	0	0	1	0	0	1	4
hsa-miR-708	CMTM4	1	1	0	1	0	0	1	0	0	1	5
hsa-miR-708	CMTM7	1	1	0	1	0	0	1	0	0	1	5
hsa-miR-708	CNGB1	1	1	0	1	0	0	1	0	0	1	5
hsa-miR-708	CNKSR1	0	1	0	1	0	0	1	0	0	1	4
hsa-miR-708	CNNM2	1	1	0	1	0	0	1	0	0	1	5
hsa-miR-708	CNNM3	1	1	0	1	0	0	1	0	0	1	5
hsa-miR-708	CNNM4	1	1	0	0	0	0	1	0	0	1	4
hsa-miR-708	CNOT6L	1	1	0	1	0	0	1	0	0	0	4
hsa-miR-708	CNP	1	1	0	1	0	0	1	0	0	1	5
hsa-miR-708	CNPY3	1	1	0	1	0	0	1	0	0	1	5
hsa-miR-708	CNPY4	1	1	0	1	0	0	1	0	0	1	5
hsa-miR-708	CNR1	1	1	0	1	0	0	1	0	0	1	5
hsa-miR-708	CNTFR	1	1	0	1	0	0	0	0	0	1	4
hsa-miR-708	CNTNAP1	1	1	0	1	0	0	1	0	0	1	5
hsa-miR-708	COBRA1	1	1	0	1	0	0	1	0	0	0	4
hsa-miR-708	COL17A1	1	1	0	1	0	0	1	0	0	1	5
hsa-miR-708	COL1A1	1	1	0	1	0	0	1	0	0	1	5
hsa-miR-708	COL6A1	1	1	0	1	0	0	1	0	0	1	5
hsa-miR-708	COL8A2	1	1	0	1	0	0	1	0	0	1	5
hsa-miR-708	COLQ	1	1	0	1	0	0	0	0	0	1	4
hsa-miR-708	COMMD6	1	1	0	1	0	0	1	0	0	0	4
hsa-miR-708	COMMD9	1	1	1	1	0	0	1	0	0	1	6
hsa-miR-708	COPG2	0	1	0	1	0	0	1	0	0	1	4
hsa-miR-708	CORO1B	1	1	0	1	0	0	1	0	0	1	5
hsa-miR-708	CORO1C	1	1	0	1	0	0	1	0	0	1	5
hsa-miR-708	COX19	0	1	0	1	0	0	1	0	0	1	4
hsa-miR-708	COX5B	1	1	0	1	0	0	1	0	0	0	4
hsa-miR-708	COX6B2	1	1	0	1	0	0	1	0	0	1	5
hsa-miR-708	CPD	1	1	0	1	0	0	1	0	0	1	5
hsa-miR-708	CPLX1	1	1	0	1	0	0	1	0	0	1	5
hsa-miR-708	CPNE5	1	1	0	0	0	0	1	0	0	1	4
hsa-miR-708	CPSF2	1	1	0	1	0	0	1	0	0	1	5
hsa-miR-708	CR1	1	1	0	1	0	0	1	0	0	1	5
hsa-miR-708	CRAT	1	1	0	1	0	0	1	0	0	1	5
hsa-miR-708	CREB1	1	1	0	1	0	0	1	0	0	1	5
hsa-miR-708	CREB3	1	1	0	0	0	0	1	0	0	1	4
hsa-miR-708	CREB3L2	1	1	0	1	0	0	1	0	0	1	5
hsa-miR-708	CREB5	1	1	0	1	0	0	1	0	0	1	5
hsa-miR-708	CREBL2	1	1	0	1	0	0	1	0	0	1	5
hsa-miR-708	CREG2	1	1	0	1	0	0	1	0	0	1	5
hsa-miR-708	CRHR1	1	1	0	1	0	0	1	0	0	0	4
hsa-miR-708	CRIM1	1	1	0	1	0	0	1	0	0	1	5

hsa-miR-708	CROT	1	1	1	1	0	0	1	0	0	1	6
hsa-miR-708	CRTC3	1	1	0	1	0	0	1	0	0	1	5
hsa-miR-708	CSF1	1	1	0	1	0	0	1	0	0	1	5
hsa-miR-708	CSF3	1	1	0	1	0	0	0	0	0	1	4
hsa-miR-708	CSMD2	1	0	0	1	0	0	1	0	0	1	4
hsa-miR-708	CSNK1G1	1	1	0	1	0	0	1	0	0	1	5
hsa-miR-708	CSNK2A1	1	1	0	1	0	0	0	0	0	1	4
hsa-miR-708	CST7	1	1	0	1	0	0	1	0	0	0	4
hsa-miR-708	CTAG2	1	1	0	1	0	0	1	0	0	0	4
hsa-miR-708	CTCFL	1	1	0	1	0	0	1	0	0	0	4
hsa-miR-708	CTDSPL	1	1	0	1	0	0	1	0	0	1	5
hsa-miR-708	CTF8	1	0	0	1	0	0	1	0	0	1	4
hsa-miR-708	CTNNBIP1	1	1	0	1	0	0	0	0	0	1	4
hsa-miR-708	CTNS	1	1	0	1	0	0	1	0	0	0	4
hsa-miR-708	CTSC	0	1	0	1	0	0	1	0	0	1	4
hsa-miR-708	CTSS	1	1	0	1	0	0	1	0	0	0	4
hsa-miR-708	CUL4B	1	1	0	1	0	0	1	0	0	1	5
hsa-miR-708	CXADR	1	1	0	1	0	0	1	0	0	1	5
hsa-miR-708	CXCL12	1	1	0	1	0	0	1	0	0	1	5
hsa-miR-708	CXCL5	1	1	0	1	0	0	1	0	0	1	5
hsa-miR-708	CXCL9	1	1	0	1	0	0	1	0	0	1	5
hsa-miR-708	CXCR3	1	1	0	1	0	0	1	0	0	0	4
hsa-miR-708	CXCR5	1	1	0	1	0	0	0	0	0	1	4
hsa-miR-708	CXorf15	1	1	0	1	0	0	1	0	0	0	4
hsa-miR-708	CYB561	1	1	0	1	0	0	1	0	0	0	4
hsa-miR-708	CYB5D1	1	1	0	1	0	0	1	0	0	1	5
hsa-miR-708	CYBASC3	1	0	0	1	0	0	1	0	0	1	4
hsa-miR-708	CYBB	1	1	0	1	0	0	1	0	0	1	5
hsa-miR-708	CYP11B1	1	1	0	1	0	0	1	0	0	1	5
hsa-miR-708	CYP11B2	1	1	0	1	0	0	1	0	0	1	5
hsa-miR-708	CYP26B1	1	1	0	1	0	0	1	0	0	1	5
hsa-miR-708	CYP27C1	1	1	0	1	0	0	1	0	0	1	5
hsa-miR-708	CYP2B6	1	1	0	1	0	0	1	0	0	1	5
hsa-miR-708	CYP2U1	1	1	0	1	0	0	1	0	0	1	5
hsa-miR-708	CYP4F22	1	1	0	1	0	0	1	0	0	1	5
hsa-miR-708	CYP4V2	1	1	0	1	0	0	1	0	0	0	4
hsa-miR-708	CYP8B1	1	1	0	1	0	0	1	0	0	1	5
hsa-miR-708	D4S234E	1	0	1	1	0	0	1	0	0	1	5
hsa-miR-708	DAB2IP	1	1	0	1	0	0	1	0	0	1	5
hsa-miR-708	DAK	1	1	0	1	0	0	1	0	0	1	5
hsa-miR-708	DALRD3	1	1	0	1	0	0	0	0	0	1	4
hsa-miR-708	DAO	1	1	0	1	0	0	1	0	0	0	4
hsa-miR-708	DAP	1	1	1	1	0	0	1	0	0	1	6
hsa-miR-708	DBF4B	1	1	0	1	0	0	1	0	0	0	4

hsa-miR-708	DBNDD1	1	0	0	1	0	0	1	0	0	1	4
hsa-miR-708	DBT	1	1	0	1	0	0	1	0	0	0	4
hsa-miR-708	DCHS1	1	1	0	1	0	0	1	0	0	1	5
hsa-miR-708	DCLK1	1	1	0	1	0	0	1	0	0	1	5
hsa-miR-708	DCLK3	1	1	0	1	0	0	1	0	0	1	5
hsa-miR-708	DCTD	1	1	0	1	0	0	1	0	0	1	5
hsa-miR-708	DCX	1	1	1	1	0	0	1	0	0	1	6
hsa-miR-708	DDAH1	1	1	0	1	0	0	0	0	0	1	4
hsa-miR-708	DDEF2	1	1	0	1	0	0	1	0	0	1	5
hsa-miR-708	DDN	1	1	0	1	0	0	1	0	0	0	4
hsa-miR-708	DDX18	1	1	0	1	0	0	1	0	0	1	5
hsa-miR-708	DDX21	1	1	0	1	0	0	1	0	0	1	5
hsa-miR-708	DDX28	1	1	0	1	0	0	1	0	0	0	4
hsa-miR-708	DDX4	1	0	0	1	0	0	1	0	0	1	4
hsa-miR-708	DDX56	1	1	0	1	0	0	1	0	0	1	5
hsa-miR-708	DDX60L	1	1	0	1	0	0	1	0	0	1	5
hsa-miR-708	DEDD	1	1	0	1	0	0	0	0	0	1	4
hsa-miR-708	DEPDC2	1	1	0	1	0	0	1	0	0	1	5
hsa-miR-708	DERL1	1	1	0	1	0	0	1	0	0	1	5
hsa-miR-708	DFFB	1	1	0	1	0	0	1	0	0	1	5
hsa-miR-708	DFNB31	1	1	0	1	0	0	1	0	0	1	5
hsa-miR-708	DGCR14	1	1	0	1	0	0	1	0	0	1	5
hsa-miR-708	DGCR8	1	1	0	1	0	0	1	0	0	0	4
hsa-miR-708	DGKQ	1	1	0	1	0	0	1	0	0	1	5
hsa-miR-708	DHRS2	1	1	0	1	0	0	1	0	0	1	5
hsa-miR-708	DHRS3	1	1	0	1	0	0	1	0	0	1	5
hsa-miR-708	DHRS7B	1	1	0	1	0	0	1	0	0	1	5
hsa-miR-708	DHX16	1	1	0	1	0	0	0	0	0	1	4
hsa-miR-708	DHX33	1	1	0	1	0	0	1	0	0	1	5
hsa-miR-708	DHX35	1	1	0	1	0	0	1	0	0	1	5
hsa-miR-708	DHX40	1	1	0	1	0	0	1	0	0	0	4
hsa-miR-708	DIAPH1	1	1	0	1	0	0	1	0	0	1	5
hsa-miR-708	DIP2B	1	1	0	1	0	0	1	0	0	1	5
hsa-miR-708	DIP2C	1	1	0	1	0	0	1	0	0	1	5
hsa-miR-708	DIXDC1	1	0	0	1	0	0	1	0	0	1	4
hsa-miR-708	DKK3	1	1	0	1	0	0	1	0	0	1	5
hsa-miR-708	DLG3	1	1	0	1	0	0	1	0	0	0	4
hsa-miR-708	DLGAP2	1	1	0	1	0	0	1	0	0	1	5
hsa-miR-708	DLL4	1	1	0	1	0	0	1	0	0	1	5
hsa-miR-708	DMKN	0	1	0	1	0	0	1	0	0	1	4
hsa-miR-708	DNAJA4	1	1	0	0	0	0	1	0	0	1	4
hsa-miR-708	DNAJB14	1	1	0	1	0	0	0	0	0	1	4
hsa-miR-708	DNAJB5	1	0	0	1	0	0	1	0	0	1	4
hsa-miR-708	DNAJC16	1	1	0	1	0	0	1	0	0	1	5

hsa-miR-708	DNAJC8	1	1	0	1	0	0	1	0	0	1	5
hsa-miR-708	DNAL1	0	1	0	1	0	0	1	0	0	1	4
hsa-miR-708	DNASE2	1	1	0	1	0	0	1	0	0	1	5
hsa-miR-708	DNM3	0	1	1	1	0	0	1	0	0	1	5
hsa-miR-708	DNMBP	1	1	0	1	0	0	1	0	0	1	5
hsa-miR-708	DOCK1	1	1	0	1	0	0	1	0	0	1	5
hsa-miR-708	DOCK3	1	1	0	1	0	0	1	0	0	1	5
hsa-miR-708	DOCK4	1	1	0	1	0	0	1	0	0	1	5
hsa-miR-708	DOK4	1	1	0	1	0	0	1	0	0	0	4
hsa-miR-708	DPF1	1	0	0	1	0	0	1	0	0	1	4
hsa-miR-708	DPY19L1	1	0	0	1	0	0	1	0	0	1	4
hsa-miR-708	DPYSL2	1	1	0	1	0	0	1	0	0	1	5
hsa-miR-708	DRAM	1	1	0	1	0	0	1	0	0	1	5
hsa-miR-708	DSCR6	1	1	0	1	0	0	1	0	0	1	5
hsa-miR-708	DSEL	1	1	0	0	0	0	1	0	0	1	4
hsa-miR-708	DSG3	1	1	0	1	0	0	1	0	0	1	5
hsa-miR-708	DTNA	1	1	0	1	0	0	0	0	0	1	4
hsa-miR-708	DTNBP1	1	0	0	1	0	0	1	0	0	1	4
hsa-miR-708	DUOX2	1	1	0	1	0	0	1	0	0	1	5
hsa-miR-708	DUSP1	1	1	0	1	0	0	1	0	0	0	4
hsa-miR-708	DUSP3	1	1	0	1	0	0	1	0	0	1	5
hsa-miR-708	DUSP4	1	1	0	1	0	0	0	0	0	1	4
hsa-miR-708	DVL1	1	1	0	1	0	0	1	0	0	1	5
hsa-miR-708	DYNC1I1	1	1	0	1	0	0	1	0	0	0	4
hsa-miR-708	DYNC1LI2	1	1	1	1	0	0	1	0	0	1	6
hsa-miR-708	DYSF	1	1	0	1	0	0	0	0	0	1	4
hsa-miR-708	E2F4	1	1	0	1	0	0	1	0	0	1	5
hsa-miR-708	EAF1	1	1	0	1	0	0	1	0	0	1	5
hsa-miR-708	ECE2	0	1	0	1	0	0	1	0	0	1	4
hsa-miR-708	ECHDC3	1	1	0	1	0	0	1	0	0	0	4
hsa-miR-708	EDA2R	1	1	0	1	0	0	1	0	0	1	5
hsa-miR-708	EDARADD	1	0	0	1	0	0	1	0	0	1	4
hsa-miR-708	EDEM1	1	1	0	1	0	0	1	0	0	1	5
hsa-miR-708	EDG1	1	1	0	1	0	0	1	0	0	1	5
hsa-miR-708	EDG4	1	1	0	1	0	0	1	0	0	0	4
hsa-miR-708	EEF2K	1	1	0	1	0	0	1	0	0	0	4
hsa-miR-708	EEPD1	1	1	0	1	0	0	1	0	0	1	5
hsa-miR-708	EFHD1	1	1	0	1	0	0	1	0	0	0	4
hsa-miR-708	EFHD2	1	1	0	1	0	0	1	0	0	1	5
hsa-miR-708	EFNA5	1	1	0	1	0	0	1	0	0	1	5
hsa-miR-708	EFNB1	1	1	0	1	0	0	1	0	0	1	5
hsa-miR-708	EFNB3	1	1	0	1	0	0	1	0	0	1	5
hsa-miR-708	EFTUD2	1	1	0	1	0	0	1	0	0	1	5
hsa-miR-708	EGLN2	1	1	0	1	0	0	0	0	0	1	4

hsa-miR-708	EGR2	1	1	0	1	0	0	1	0	0	0	4
hsa-miR-708	EGR3	1	1	0	1	0	0	1	0	0	0	4
hsa-miR-708	EHD1	1	1	0	1	0	0	1	0	0	1	5
hsa-miR-708	EHF	1	1	0	1	0	0	1	0	0	1	5
hsa-miR-708	EIF2A	1	1	0	1	0	0	1	0	0	1	5
hsa-miR-708	EIF2AK1	1	1	1	1	0	0	1	0	0	1	6
hsa-miR-708	EIF2C1	1	1	1	1	0	0	1	0	0	1	6
hsa-miR-708	EIF4E3	1	1	0	1	0	0	0	0	0	1	4
hsa-miR-708	ELAVL1	0	1	0	1	0	0	1	0	0	1	4
hsa-miR-708	ELFN2	0	1	0	1	0	0	1	0	0	1	4
hsa-miR-708	ELOVL7	1	1	0	1	0	0	0	0	0	1	4
hsa-miR-708	EMID2	1	1	0	0	0	0	1	0	0	1	4
hsa-miR-708	EMILIN2	1	1	0	1	0	0	1	0	0	0	4
hsa-miR-708	EMX2	1	1	0	1	0	0	1	0	0	0	4
hsa-miR-708	EN2	1	1	1	1	0	0	1	0	0	1	6
hsa-miR-708	ENAH	1	1	0	1	0	0	1	0	0	1	5
hsa-miR-708	ENG	1	1	0	1	0	0	1	0	0	1	5
hsa-miR-708	ENO2	1	1	0	1	0	0	1	0	0	0	4
hsa-miR-708	ENPEP	0	1	0	1	0	0	1	0	0	1	4
hsa-miR-708	ENPP5	1	1	0	1	0	0	1	0	0	0	4
hsa-miR-708	ENTPD2	1	1	0	0	0	0	1	0	0	1	4
hsa-miR-708	EP400	1	1	0	1	0	0	1	0	0	1	5
hsa-miR-708	EPB41L1	1	1	0	1	0	0	1	0	0	1	5
hsa-miR-708	EPB41L4A	1	1	0	1	0	0	1	0	0	1	5
hsa-miR-708	EPB41L4B	1	1	0	1	0	0	1	0	0	1	5
hsa-miR-708	EPB41L5	1	1	0	1	0	0	1	0	0	1	5
hsa-miR-708	EPDR1	1	1	0	1	0	0	1	0	0	1	5
hsa-miR-708	EPHA3	1	1	0	1	0	0	1	0	0	1	5
hsa-miR-708	EPHA4	1	1	0	1	0	0	1	0	0	0	4
hsa-miR-708	EPHB1	1	1	0	1	0	0	1	0	0	1	5
hsa-miR-708	EPN2	1	1	0	1	0	0	1	0	0	1	5
hsa-miR-708	EPN3	1	1	0	1	0	0	1	0	0	1	5
hsa-miR-708	ERBB4	1	1	0	0	0	0	1	0	0	1	4
hsa-miR-708	ERC1	1	1	0	1	0	0	0	0	0	1	4
hsa-miR-708	EREG	1	1	0	1	0	0	1	0	0	0	4
hsa-miR-708	ERICH1	1	1	0	1	0	0	1	0	0	1	5
hsa-miR-708	ERLIN1	1	1	0	1	0	0	0	0	0	1	4
hsa-miR-708	ERLIN2	1	1	0	1	0	0	1	0	0	1	5
hsa-miR-708	ESCO2	1	1	1	1	0	0	1	0	0	1	6
hsa-miR-708	ETF1	1	1	1	1	0	0	1	0	0	1	6
hsa-miR-708	ETNK1	1	1	0	0	0	0	1	0	0	1	4
hsa-miR-708	ETNK2	1	1	0	1	0	0	1	0	0	1	5
hsa-miR-708	ETS2	1	1	0	1	0	0	1	0	0	0	4
hsa-miR-708	ETV6	1	1	0	1	0	0	1	0	0	1	5

hsa-miR-708	EVC	1	1	0	1	0	0	1	0	0	1	5
hsa-miR-708	EXDL1	1	1	0	1	0	0	1	0	0	0	4
hsa-miR-708	EXOC2	1	1	0	1	0	0	1	0	0	0	4
hsa-miR-708	EXOC3	1	1	0	1	0	0	1	0	0	0	4
hsa-miR-708	EXOC7	1	0	0	1	0	0	1	0	0	1	4
hsa-miR-708	EXOSC5	1	1	0	1	0	0	1	0	0	1	5
hsa-miR-708	EXTL3	1	1	0	1	0	0	1	0	0	1	5
hsa-miR-708	EYA3	1	1	0	0	0	0	1	0	0	1	4
hsa-miR-708	EZH1	1	1	0	1	0	0	1	0	0	1	5
hsa-miR-708	F2RL2	1	1	0	1	0	0	1	0	0	1	5
hsa-miR-708	F8	1	1	0	1	0	0	1	0	0	0	4
hsa-miR-708	FADD	1	1	0	1	0	0	1	0	0	1	5
hsa-miR-708	FAIM2	1	1	1	1	0	0	1	0	0	1	6
hsa-miR-708	FAM100A	1	1	0	0	0	0	1	0	0	1	4
hsa-miR-708	FAM102A	1	1	0	1	0	0	1	0	0	1	5
hsa-miR-708	FAM105B	1	1	0	1	0	0	1	0	0	1	5
hsa-miR-708	FAM107A	1	0	1	1	0	0	1	0	0	1	5
hsa-miR-708	FAM107B	1	1	0	1	0	0	1	0	0	0	4
hsa-miR-708	FAM109A	1	1	0	1	0	0	1	0	0	1	5
hsa-miR-708	FAM119B	1	1	0	1	0	0	1	0	0	1	5
hsa-miR-708	FAM123B	1	1	0	1	0	0	1	0	0	1	5
hsa-miR-708	FAM123C	0	1	0	1	0	0	1	0	0	1	4
hsa-miR-708	FAM125B	1	1	0	1	0	0	1	0	0	1	5
hsa-miR-708	FAM126A	1	1	1	1	0	0	1	0	0	1	6
hsa-miR-708	FAM127B	1	1	0	1	0	0	1	0	0	1	5
hsa-miR-708	FAM129B	1	1	0	1	0	0	1	0	0	0	4
hsa-miR-708	FAM131B	1	1	0	1	0	0	1	0	0	1	5
hsa-miR-708	FAM134A	1	1	0	1	0	0	1	0	0	1	5
hsa-miR-708	FAM134C	1	1	0	1	0	0	1	0	0	1	5
hsa-miR-708	FAM135B	1	1	0	1	0	0	1	0	0	1	5
hsa-miR-708	FAM18A	1	1	0	1	0	0	1	0	0	0	4
hsa-miR-708	FAM18B2	1	1	0	1	0	0	1	0	0	1	5
hsa-miR-708	FAM26B	1	1	0	1	0	0	1	0	0	1	5
hsa-miR-708	FAM26E	1	1	0	1	0	0	1	0	0	1	5
hsa-miR-708	FAM32A	1	1	0	1	0	0	1	0	0	1	5
hsa-miR-708	FAM38B	1	1	0	1	0	0	1	0	0	1	5
hsa-miR-708	FAM3D	1	1	0	1	0	0	1	0	0	1	5
hsa-miR-708	FAM43A	0	1	0	1	0	0	1	0	0	1	4
hsa-miR-708	FAM46C	1	1	0	1	0	0	1	0	0	1	5
hsa-miR-708	FAM53B	1	1	0	0	0	0	1	0	0	1	4
hsa-miR-708	FAM53C	1	0	0	1	0	0	1	0	0	1	4
hsa-miR-708	FAM5B	1	1	0	1	0	0	1	0	0	1	5
hsa-miR-708	FAM62A	1	1	0	1	0	0	1	0	0	1	5
hsa-miR-708	FAM71F2	1	1	0	1	0	0	1	0	0	0	4

hsa-miR-708	FAM73A	1	1	0	1	0	0	1	0	0	1	5
hsa-miR-708	FAM78A	1	1	0	1	0	0	1	0	0	1	5
hsa-miR-708	FAM84A	1	1	0	1	0	0	1	0	0	1	5
hsa-miR-708	FAM91A1	1	1	0	1	0	0	1	0	0	1	5
hsa-miR-708	FAM96B	1	1	0	1	0	0	1	0	0	1	5
hsa-miR-708	FAM9A	1	1	0	1	0	0	1	0	0	1	5
hsa-miR-708	FAM9B	1	1	0	0	0	0	1	0	0	1	4
hsa-miR-708	FASLG	1	1	0	1	0	0	1	0	0	1	5
hsa-miR-708	FBXL16	1	1	0	0	0	0	1	0	0	1	4
hsa-miR-708	FBXL18	1	1	1	1	0	0	1	0	0	1	6
hsa-miR-708	FBXO21	1	1	0	1	0	0	1	0	0	1	5
hsa-miR-708	FBXO40	1	1	0	1	0	0	1	0	0	1	5
hsa-miR-708	FBXO41	1	1	0	1	0	0	1	0	0	1	5
hsa-miR-708	FBXO45	1	1	1	1	0	0	1	0	0	1	6
hsa-miR-708	FBXW2	1	1	0	1	0	0	1	0	0	1	5
hsa-miR-708	FBXW4	1	1	0	1	0	0	1	0	0	1	5
hsa-miR-708	FCRL1	1	1	0	1	0	0	1	0	0	0	4
hsa-miR-708	FCRL3	1	1	0	1	0	0	1	0	0	1	5
hsa-miR-708	FECH	1	1	0	1	0	0	1	0	0	1	5
hsa-miR-708	FEM1C	1	1	0	1	0	0	1	0	0	1	5
hsa-miR-708	FERMT1	1	1	0	1	0	0	1	0	0	1	5
hsa-miR-708	FES	1	1	0	1	0	0	1	0	0	1	5
hsa-miR-708	FEV	1	1	1	1	0	0	1	0	0	1	6
hsa-miR-708	FGD2	1	1	0	1	0	0	1	0	0	1	5
hsa-miR-708	FGF11	1	1	0	1	0	0	1	0	0	1	5
hsa-miR-708	FGF23	1	1	1	1	0	0	1	0	0	1	6
hsa-miR-708	FGF3	1	1	0	1	0	0	1	0	0	1	5
hsa-miR-708	FHDC1	1	1	0	1	0	0	1	0	0	1	5
hsa-miR-708	FHL3	1	1	0	1	0	0	1	0	0	0	4
hsa-miR-708	FHOD1	1	1	0	1	0	0	1	0	0	1	5
hsa-miR-708	FIBIN	1	1	0	1	0	0	1	0	0	1	5
hsa-miR-708	FIGN	1	1	0	1	0	0	1	0	0	0	4
hsa-miR-708	FKBP10	1	1	0	1	0	0	1	0	0	1	5
hsa-miR-708	FKTN	0	0	1	1	0	0	1	0	0	1	4
hsa-miR-708	FLJ10213	0	1	1	1	0	0	1	0	0	1	5
hsa-miR-708	FLJ10357	1	1	0	1	0	0	1	0	0	1	5
hsa-miR-708	FLJ10490	0	1	0	1	0	0	1	0	0	1	4
hsa-miR-708	FLJ10769	1	1	0	1	0	0	1	0	0	1	5
hsa-miR-708	FLJ11151	0	1	0	1	0	0	1	0	0	1	4
hsa-miR-708	FLJ11184	1	1	1	1	0	0	1	0	0	1	6
hsa-miR-708	FLJ11506	1	1	0	0	0	0	1	0	0	1	4
hsa-miR-708	FLJ12529	1	1	0	1	0	0	1	0	0	1	5
hsa-miR-708	FLJ14213	1	1	0	1	0	0	1	0	0	1	5
hsa-miR-708	FLJ20309	1	1	0	1	0	0	1	0	0	1	5

hsa-miR-708	FLJ20489	1	1	0	1	0	0	1	0	0	0	4
hsa-miR-708	FLJ20674	1	1	0	1	0	0	1	0	0	1	5
hsa-miR-708	FLJ21062	1	1	0	1	0	0	0	0	0	1	4
hsa-miR-708	FLJ21865	1	1	0	1	0	0	1	0	0	1	5
hsa-miR-708	FLJ30851	1	1	0	1	0	0	1	0	0	1	5
hsa-miR-708	FLJ36031	0	1	0	1	0	0	1	0	0	1	4
hsa-miR-708	FLJ36070	1	1	0	1	0	0	0	0	0	1	4
hsa-miR-708	FLJ37078	1	1	0	1	0	0	0	0	0	1	4
hsa-miR-708	FLJ39743	0	1	0	1	0	0	1	0	0	1	4
hsa-miR-708	FLJ45224	1	1	0	1	0	0	1	0	0	0	4
hsa-miR-708	FLYWCH1	1	1	0	1	0	0	1	0	0	1	5
hsa-miR-708	FMNL3	1	1	1	1	0	0	1	0	0	1	6
hsa-miR-708	FMO2	1	1	0	1	0	0	1	0	0	1	5
hsa-miR-708	FNBP1	1	1	0	1	0	0	1	0	0	1	5
hsa-miR-708	FNDC5	1	1	0	1	0	0	1	0	0	0	4
hsa-miR-708	FNDC7	0	0	1	1	0	0	1	0	0	1	4
hsa-miR-708	FNTA	1	1	0	1	0	0	1	0	0	1	5
hsa-miR-708	FOSL2	1	1	0	1	0	0	1	0	0	1	5
hsa-miR-708	FOXJ3	1	1	1	0	0	0	1	0	0	1	5
hsa-miR-708	FOXK1	1	1	0	1	0	0	1	0	0	1	5
hsa-miR-708	FOXK2	1	1	0	1	0	0	1	0	0	1	5
hsa-miR-708	FOXL2	0	1	0	1	0	0	1	0	0	1	4
hsa-miR-708	FOXN1	1	1	0	1	0	0	1	0	0	1	5
hsa-miR-708	FOXN3	1	1	0	1	0	0	0	0	0	1	4
hsa-miR-708	FOXO1	1	1	1	1	0	0	1	0	0	1	6
hsa-miR-708	FOXO3	0	1	0	1	0	0	1	0	0	1	4
hsa-miR-708	FOXO4	1	1	0	1	0	0	1	0	0	0	4
hsa-miR-708	FOXP1	1	1	0	0	0	0	1	0	0	1	4
hsa-miR-708	FRAS1	1	1	0	1	0	0	1	0	0	1	5
hsa-miR-708	FREM2	1	1	0	0	0	0	1	0	0	1	4
hsa-miR-708	FREQ	0	1	0	1	0	0	1	0	0	1	4
hsa-miR-708	FRMD4A	1	1	0	0	0	0	1	0	0	1	4
hsa-miR-708	FRMD4B	1	1	0	1	0	0	1	0	0	1	5
hsa-miR-708	FRMD8	1	1	0	1	0	0	1	0	0	0	4
hsa-miR-708	FRS2	1	1	0	1	0	0	0	0	0	1	4
hsa-miR-708	FSHB	1	0	1	1	0	0	1	0	0	1	5
hsa-miR-708	FSTL3	1	1	0	1	0	0	1	0	0	0	4
hsa-miR-708	FSTL4	1	1	0	0	0	0	1	0	0	1	4
hsa-miR-708	FTSJ2	1	1	1	1	0	0	1	0	0	1	6
hsa-miR-708	FUT8	1	0	0	1	0	0	1	0	0	1	4
hsa-miR-708	FXC1	1	1	0	1	0	0	1	0	0	0	4
hsa-miR-708	FYCO1	1	1	0	0	0	0	1	0	0	1	4
hsa-miR-708	FZD1	1	1	0	1	0	0	1	0	0	1	5
hsa-miR-708	FZD10	1	1	0	1	0	0	1	0	0	1	5

hsa-miR-708	FZD4	1	1	0	1	0	0	1	0	0	1	5
hsa-miR-708	FZD5	1	1	0	1	0	0	1	0	0	0	4
hsa-miR-708	FZD8	1	1	0	1	0	0	1	0	0	1	5
hsa-miR-708	FZR1	1	0	0	1	0	0	1	0	0	1	4
hsa-miR-708	GAA	1	0	0	1	0	0	1	0	0	1	4
hsa-miR-708	GABRQ	1	1	0	1	0	0	1	0	0	0	4
hsa-miR-708	GAL3ST2	1	1	0	1	0	0	1	0	0	1	5
hsa-miR-708	ALNAC4S-6S	1	1	0	1	0	0	1	0	0	1	5
hsa-miR-708	GALNT6	1	1	1	1	0	0	1	0	0	1	6
hsa-miR-708	GALNT9	1	1	0	1	0	0	0	0	0	1	4
hsa-miR-708	GALP	1	1	0	0	0	0	1	0	0	1	4
hsa-miR-708	GANAB	1	1	0	1	0	0	1	0	0	0	4
hsa-miR-708	GAPVD1	1	1	0	1	0	0	1	0	0	1	5
hsa-miR-708	GARNL4	0	1	0	1	0	0	1	0	0	1	4
hsa-miR-708	GAS1	1	1	0	1	0	0	1	0	0	1	5
hsa-miR-708	GAS2L1	1	1	0	1	0	0	1	0	0	0	4
hsa-miR-708	GAS7	1	1	0	1	0	0	1	0	0	1	5
hsa-miR-708	GATAD2A	0	1	0	1	0	0	1	0	0	1	4
hsa-miR-708	GBP6	1	1	0	1	0	0	1	0	0	0	4
hsa-miR-708	GCHFR	1	1	0	1	0	0	1	0	0	1	5
hsa-miR-708	GCNT3	1	1	0	1	0	0	1	0	0	1	5
hsa-miR-708	GDA	1	1	0	1	0	0	1	0	0	1	5
hsa-miR-708	GDPD5	1	1	0	1	0	0	1	0	0	1	5
hsa-miR-708	GFI1B	0	1	0	1	0	0	1	0	0	1	4
hsa-miR-708	GFOD1	1	1	0	1	0	0	1	0	0	1	5
hsa-miR-708	GFPT2	1	1	0	1	0	0	1	0	0	0	4
hsa-miR-708	GGA2	1	1	0	1	0	0	1	0	0	1	5
hsa-miR-708	GGA3	1	1	0	1	0	0	1	0	0	1	5
hsa-miR-708	GIGYF1	1	1	0	1	0	0	1	0	0	1	5
hsa-miR-708	GIT2	1	1	1	1	0	0	1	0	0	1	6
hsa-miR-708	GJA3	1	1	0	1	0	0	1	0	0	1	5
hsa-miR-708	GJA5	1	0	0	1	0	0	1	0	0	1	4
hsa-miR-708	GJC2	1	1	0	1	0	0	1	0	0	1	5
hsa-miR-708	GLCCI1	1	1	0	1	0	0	1	0	0	0	4
hsa-miR-708	GLCE	1	1	0	1	0	0	1	0	0	0	4
hsa-miR-708	GLG1	1	1	0	1	0	0	1	0	0	1	5
hsa-miR-708	GLIPR1	1	1	0	1	0	0	1	0	0	0	4
hsa-miR-708	GLIS2	1	1	0	1	0	0	1	0	0	1	5
hsa-miR-708	GLIS3	1	1	0	0	0	0	1	0	0	1	4
hsa-miR-708	GLRX	1	1	0	1	0	0	1	0	0	1	5
hsa-miR-708	GLYAT	1	1	0	1	0	0	1	0	0	1	5
hsa-miR-708	GMPPA	1	1	0	1	0	0	0	0	0	1	4
hsa-miR-708	GNA12	1	1	0	1	0	0	1	0	0	1	5
hsa-miR-708	GNAI1	1	1	0	1	0	0	1	0	0	1	5

hsa-miR-708	GNAO1	1	1	0	1	0	0	0	0	0	1	4
hsa-miR-708	GNB4	0	1	0	1	0	0	1	0	0	1	4
hsa-miR-708	GNG12	1	1	0	1	0	0	1	0	0	1	5
hsa-miR-708	GNG2	1	1	0	1	0	0	1	0	0	1	5
hsa-miR-708	GNPNAT1	1	1	0	1	0	0	1	0	0	0	4
hsa-miR-708	GON4L	1	1	1	1	0	0	1	0	0	1	6
hsa-miR-708	GORASP2	1	1	0	1	0	0	1	0	0	0	4
hsa-miR-708	GOSR1	1	0	0	1	0	0	1	0	0	1	4
hsa-miR-708	GPA33	1	1	0	0	0	0	1	0	0	1	4
hsa-miR-708	GPD1	1	1	0	1	0	0	1	0	0	1	5
hsa-miR-708	GPER	1	0	0	1	0	0	1	0	0	1	4
hsa-miR-708	GPM6A	1	0	1	1	0	0	1	0	0	1	5
hsa-miR-708	GPR107	1	1	0	1	0	0	1	0	0	1	5
hsa-miR-708	GPR116	1	1	0	1	0	0	1	0	0	1	5
hsa-miR-708	GPR158	1	1	0	1	0	0	1	0	0	1	5
hsa-miR-708	GPR26	1	1	1	1	0	0	1	0	0	1	6
hsa-miR-708	GPR37L1	1	1	0	1	0	0	1	0	0	0	4
hsa-miR-708	GPR44	1	1	0	1	0	0	1	0	0	1	5
hsa-miR-708	GPR45	1	1	0	1	0	0	1	0	0	1	5
hsa-miR-708	GPR64	1	1	0	1	0	0	0	0	0	1	4
hsa-miR-708	GPR81	0	1	0	1	0	0	1	0	0	1	4
hsa-miR-708	GPR83	1	1	0	1	0	0	1	0	0	1	5
hsa-miR-708	GPR88	1	1	0	1	0	0	1	0	0	1	5
hsa-miR-708	GPR97	1	1	0	1	0	0	1	0	0	1	5
hsa-miR-708	GPRC5A	1	1	0	1	0	0	1	0	0	0	4
hsa-miR-708	GREM2	1	1	0	1	0	0	1	0	0	1	5
hsa-miR-708	GRHL2	1	1	0	1	0	0	1	0	0	1	5
hsa-miR-708	GRIA4	1	1	0	1	0	0	1	0	0	1	5
hsa-miR-708	GRIK3	0	1	0	1	0	0	1	0	0	1	4
hsa-miR-708	GRIN2B	1	1	0	1	0	0	1	0	0	1	5
hsa-miR-708	GRIN3A	1	1	0	1	0	0	1	0	0	1	5
hsa-miR-708	GRLF1	0	1	0	1	0	0	1	0	0	1	4
hsa-miR-708	GRM1	1	1	0	1	0	0	0	0	0	1	4
hsa-miR-708	GRM4	1	1	0	1	0	0	1	0	0	1	5
hsa-miR-708	GRSF1	0	1	0	1	0	0	1	0	0	1	4
hsa-miR-708	GSG1L	1	1	0	1	0	0	1	0	0	0	4
hsa-miR-708	GSK3A	1	1	0	1	0	0	1	0	0	1	5
hsa-miR-708	GSTCD	1	0	0	1	0	0	1	0	0	1	4
hsa-miR-708	GTF3C1	1	1	0	1	0	0	1	0	0	0	4
hsa-miR-708	GTSE1	1	0	0	1	0	0	1	0	0	1	4
hsa-miR-708	GUCA2B	1	1	0	1	0	0	1	0	0	1	5
hsa-miR-708	GUCY2D	1	1	0	1	0	0	1	0	0	0	4
hsa-miR-708	GZF1	1	1	0	1	0	0	1	0	0	1	5
hsa-miR-708	H3F3B	1	1	1	1	0	0	1	0	0	1	6

hsa-miR-708	HACE1	1	1	1	1	0	0	1	0	0	1	6
hsa-miR-708	HADHB	1	1	0	1	0	0	1	0	0	1	5
hsa-miR-708	HAPLN4	1	1	0	1	0	0	1	0	0	1	5
hsa-miR-708	HBP1	1	1	0	1	0	0	1	0	0	1	5
hsa-miR-708	HDGF	1	1	0	1	0	0	1	0	0	1	5
hsa-miR-708	HDLBP	1	0	0	1	0	0	1	0	0	1	4
hsa-miR-708	HECA	1	1	0	1	0	0	1	0	0	1	5
hsa-miR-708	HEG1	1	1	0	0	0	0	1	0	0	1	4
hsa-miR-708	HELZ	0	1	0	1	0	0	1	0	0	1	4
hsa-miR-708	HEPH	1	1	0	1	0	0	0	0	0	1	4
hsa-miR-708	HGSNAT	0	1	0	1	0	0	1	0	0	1	4
hsa-miR-708	HHAT	1	0	0	1	0	0	1	0	0	1	4
hsa-miR-708	HIF1AN	1	1	0	1	0	0	1	0	0	1	5
hsa-miR-708	HIF3A	1	0	0	1	0	0	1	0	0	1	4
hsa-miR-708	HIG2	1	1	0	1	0	0	0	0	0	1	4
hsa-miR-708	HIPK1	1	1	0	1	0	0	1	0	0	1	5
hsa-miR-708	HIPK4	1	1	0	1	0	0	1	0	0	0	4
hsa-miR-708	HIST2H2BE	1	1	0	1	0	0	1	0	0	0	4
hsa-miR-708	HJURP	1	1	0	1	0	0	1	0	0	1	5
hsa-miR-708	HK2	1	1	0	1	0	0	1	0	0	1	5
hsa-miR-708	HLA-DOA	1	1	0	0	0	0	1	0	0	1	4
hsa-miR-708	HLA-DRB1	1	1	0	1	0	0	0	0	0	1	4
hsa-miR-708	HMG20B	1	1	0	1	0	0	1	0	0	0	4
hsa-miR-708	HMGA1	1	1	0	1	0	0	1	0	0	0	4
hsa-miR-708	HMGB3	1	1	0	1	0	0	1	0	0	1	5
hsa-miR-708	HMGCLL1	1	1	0	1	0	0	1	0	0	1	5
hsa-miR-708	HMP19	1	1	0	1	0	0	1	0	0	1	5
hsa-miR-708	HNF1A	1	1	0	1	0	0	1	0	0	1	5
hsa-miR-708	HNRPA3	1	1	0	1	0	0	1	0	0	0	4
hsa-miR-708	HNRPDL	1	1	0	0	0	0	1	0	0	1	4
hsa-miR-708	HNRPK	1	1	0	1	0	0	1	0	0	1	5
hsa-miR-708	HNRPUL1	1	1	0	1	0	0	1	0	0	0	4
hsa-miR-708	HNT	1	0	0	1	0	0	1	0	0	1	4
hsa-miR-708	HOXA1	1	1	1	1	0	0	1	0	0	1	6
hsa-miR-708	HOXB2	1	1	0	1	0	0	1	0	0	0	4
hsa-miR-708	HOXB3	1	1	1	1	0	0	1	0	0	1	6
hsa-miR-708	HOXB4	1	1	0	1	0	0	1	0	0	1	5
hsa-miR-708	HOXB9	1	1	0	1	0	0	1	0	0	1	5
hsa-miR-708	HOXC10	1	1	0	1	0	0	1	0	0	0	4
hsa-miR-708	HPS1	1	1	0	0	0	0	1	0	0	1	4
hsa-miR-708	HRH4	1	1	0	1	0	0	1	0	0	1	5
hsa-miR-708	HS2ST1	1	1	0	1	0	0	1	0	0	1	5
hsa-miR-708	HS6ST3	0	1	0	1	0	0	1	0	0	1	4
hsa-miR-708	HSPA12A	1	1	0	1	0	0	1	0	0	0	4

hsa-miR-708	HSPBP1	1	1	0	1	0	0	0	0	0	1	4
hsa-miR-708	HTRA2	1	1	0	1	0	0	1	0	0	1	5
hsa-miR-708	HTT	1	1	0	1	0	0	1	0	0	1	5
hsa-miR-708	HUNK	1	1	0	1	0	0	1	0	0	1	5
hsa-miR-708	HUS1	1	1	0	1	0	0	1	0	0	1	5
hsa-miR-708	HYOU1	1	1	0	1	0	0	0	0	0	1	4
hsa-miR-708	ICMT	1	1	0	1	0	0	1	0	0	1	5
hsa-miR-708	ICOS	1	1	0	1	0	0	1	0	0	1	5
hsa-miR-708	ICOSLG	1	1	0	0	0	0	1	0	0	1	4
hsa-miR-708	IDI1	1	1	0	1	0	0	1	0	0	0	4
hsa-miR-708	IER3IP1	1	1	1	1	0	0	1	0	0	1	6
hsa-miR-708	IFIT5	1	1	0	1	0	0	1	0	0	1	5
hsa-miR-708	IGF2BP1	1	1	0	1	0	0	1	0	0	1	5
hsa-miR-708	IGFBP1	1	1	0	1	0	0	1	0	0	1	5
hsa-miR-708	IGSF2	1	1	0	1	0	0	1	0	0	1	5
hsa-miR-708	IGSF9B	0	1	1	1	0	0	1	0	0	1	5
hsa-miR-708	IHPK1	1	1	0	1	0	0	1	0	0	0	4
hsa-miR-708	IKBKB	1	1	1	1	0	0	1	0	0	1	6
hsa-miR-708	IKZF1	0	1	0	1	0	0	1	0	0	1	4
hsa-miR-708	IKZF4	1	1	1	1	0	0	1	0	0	1	6
hsa-miR-708	IL16	1	1	0	1	0	0	1	0	0	1	5
hsa-miR-708	IL17RD	1	1	0	1	0	0	1	0	0	1	5
hsa-miR-708	IL17RE	1	1	0	1	0	0	1	0	0	1	5
hsa-miR-708	IL20RB	1	1	0	1	0	0	1	0	0	0	4
hsa-miR-708	ILF3	1	1	0	1	0	0	1	0	0	1	5
hsa-miR-708	IMPACT	1	1	0	1	0	0	1	0	0	1	5
hsa-miR-708	IMPAD1	0	1	0	1	0	0	1	0	0	1	4
hsa-miR-708	IMPDH1	1	1	0	1	0	0	1	0	0	1	5
hsa-miR-708	INCENP	1	1	0	1	0	0	1	0	0	1	5
hsa-miR-708	INPP5D	1	0	0	1	0	0	1	0	0	1	4
hsa-miR-708	INPP5E	1	1	0	1	0	0	1	0	0	1	5
hsa-miR-708	INSL3	0	1	0	1	0	0	1	0	0	1	4
hsa-miR-708	INSR	1	1	0	1	0	0	1	0	0	1	5
hsa-miR-708	IPO9	1	1	0	1	0	0	1	0	0	1	5
hsa-miR-708	IPPK	1	1	0	1	0	0	1	0	0	1	5
hsa-miR-708	IQGAP1	1	1	1	1	0	0	1	0	0	1	6
hsa-miR-708	IQSEC2	1	1	0	1	0	0	0	0	0	1	4
hsa-miR-708	ISG20L1	1	1	0	1	0	0	1	0	0	0	4
hsa-miR-708	ISLR	1	1	0	1	0	0	0	0	0	1	4
hsa-miR-708	ISOC1	1	1	0	1	0	0	1	0	0	1	5
hsa-miR-708	ISX	1	1	0	1	0	0	1	0	0	1	5
hsa-miR-708	ITGA10	1	1	0	0	0	0	1	0	0	1	4
hsa-miR-708	ITGA5	1	1	0	0	0	0	1	0	0	1	4
hsa-miR-708	ITGAL	1	1	0	1	0	0	1	0	0	1	5

hsa-miR-708	ITGB3	1	1	0	1	0	0	1	0	0	1	5
hsa-miR-708	ITIH3	1	1	0	1	0	0	0	0	0	1	4
hsa-miR-708	ITIH5	1	1	0	1	0	0	1	0	0	1	5
hsa-miR-708	ITPKB	1	1	0	1	0	0	1	0	0	1	5
hsa-miR-708	ITSN1	1	1	0	1	0	0	1	0	0	0	4
hsa-miR-708	IYD	1	1	0	1	0	0	1	0	0	1	5
hsa-miR-708	JAGN1	1	1	1	1	0	0	1	0	0	1	6
hsa-miR-708	JAK1	1	1	0	0	0	0	1	0	0	1	4
hsa-miR-708	JAM3	1	1	0	1	0	0	1	0	0	1	5
hsa-miR-708	JARID1C	1	1	0	0	0	0	1	0	0	1	4
hsa-miR-708	JMJD1B	0	1	0	1	0	0	1	0	0	1	4
hsa-miR-708	JMJD1C	1	1	0	1	0	0	1	0	0	1	5
hsa-miR-708	JMJD5	1	0	0	1	0	0	1	0	0	1	4
hsa-miR-708	JMJD6	0	1	0	1	0	0	1	0	0	1	4
hsa-miR-708	JPH1	1	1	0	1	0	0	1	0	0	1	5
hsa-miR-708	JPH2	1	1	0	1	0	0	1	0	0	1	5
hsa-miR-708	JRK	0	1	0	1	0	0	1	0	0	1	4
hsa-miR-708	JUB	1	1	0	0	0	0	1	0	0	1	4
hsa-miR-708	JUP	1	1	0	1	0	0	1	0	0	0	4
hsa-miR-708	KAL1	1	1	0	0	0	0	1	0	0	1	4
hsa-miR-708	KATNAL1	1	1	0	1	0	0	0	0	0	1	4
hsa-miR-708	KBTBD11	1	1	0	1	0	0	1	0	0	1	5
hsa-miR-708	KCNA7	1	1	0	1	0	0	1	0	0	1	5
hsa-miR-708	KCNC4	1	1	0	1	0	0	1	0	0	0	4
hsa-miR-708	KCNE4	1	1	0	1	0	0	1	0	0	1	5
hsa-miR-708	KCNH2	1	1	0	1	0	0	1	0	0	0	4
hsa-miR-708	KCNH8	1	1	0	1	0	0	1	0	0	1	5
hsa-miR-708	KCNJ1	1	0	1	1	0	0	1	0	0	1	5
hsa-miR-708	KCNJ12	1	1	0	1	0	0	1	0	0	0	4
hsa-miR-708	KCNK5	1	1	0	1	0	0	1	0	0	1	5
hsa-miR-708	KCNMA1	1	1	0	1	0	0	1	0	0	1	5
hsa-miR-708	KCNS1	1	1	0	1	0	0	1	0	0	0	4
hsa-miR-708	KCTD14	1	1	0	1	0	0	1	0	0	1	5
hsa-miR-708	KCTD15	1	1	0	1	0	0	1	0	0	1	5
hsa-miR-708	KCTD17	1	1	0	1	0	0	1	0	0	0	4
hsa-miR-708	KCTD2	1	1	0	1	0	0	1	0	0	1	5
hsa-miR-708	KCTD21	1	1	0	0	0	0	1	0	0	1	4
hsa-miR-708	KCTD5	1	1	0	1	0	0	1	0	0	1	5
hsa-miR-708	KDR	1	1	0	1	0	0	1	0	0	0	4
hsa-miR-708	KIAA0152	1	1	0	0	0	0	1	0	0	1	4
hsa-miR-708	KIAA0226	1	1	0	0	0	0	1	0	0	1	4
hsa-miR-708	KIAA0247	1	1	0	1	0	0	1	0	0	1	5
hsa-miR-708	KIAA0265	1	1	0	1	0	0	1	0	0	1	5
hsa-miR-708	KIAA0284	1	1	0	1	0	0	0	0	0	1	4

hsa-miR-708	KIAA0323	1	1	0	1	0	0	1	0	0	1	5
hsa-miR-708	KIAA0355	1	1	1	1	0	0	1	0	0	1	6
hsa-miR-708	KIAA0427	1	0	0	1	0	0	1	0	0	1	4
hsa-miR-708	KIAA0467	1	1	0	1	0	0	1	0	0	1	5
hsa-miR-708	KIAA0494	1	1	0	1	0	0	1	0	0	1	5
hsa-miR-708	KIAA0513	1	1	0	1	0	0	1	0	0	1	5
hsa-miR-708	KIAA0515	1	1	0	1	0	0	1	0	0	1	5
hsa-miR-708	KIAA0556	1	1	0	1	0	0	1	0	0	1	5
hsa-miR-708	KIAA0652	1	0	0	1	0	0	1	0	0	1	4
hsa-miR-708	KIAA0774	0	1	0	1	0	0	1	0	0	1	4
hsa-miR-708	KIAA0841	0	1	0	1	0	0	1	0	0	1	4
hsa-miR-708	KIAA0888	1	1	0	1	0	0	1	0	0	1	5
hsa-miR-708	KIAA1009	1	1	0	1	0	0	1	0	0	1	5
hsa-miR-708	KIAA1012	0	1	0	1	0	0	1	0	0	1	4
hsa-miR-708	KIAA1045	1	1	0	1	0	0	1	0	0	1	5
hsa-miR-708	KIAA1128	1	1	0	1	0	0	1	0	0	1	5
hsa-miR-708	KIAA1147	1	1	0	1	0	0	1	0	0	1	5
hsa-miR-708	KIAA1199	1	1	0	1	0	0	1	0	0	1	5
hsa-miR-708	KIAA1211	1	1	0	1	0	0	1	0	0	1	5
hsa-miR-708	KIAA1303	1	1	0	1	0	0	1	0	0	1	5
hsa-miR-708	KIAA1377	1	1	0	1	0	0	1	0	0	0	4
hsa-miR-708	KIAA1430	0	1	0	1	0	0	1	0	0	1	4
hsa-miR-708	KIAA1467	1	1	0	1	0	0	1	0	0	0	4
hsa-miR-708	KIAA1522	1	1	0	0	0	0	1	0	0	1	4
hsa-miR-708	KIAA1553	1	1	0	0	0	0	1	0	0	1	4
hsa-miR-708	KIAA1576	1	1	0	1	0	0	1	0	0	1	5
hsa-miR-708	KIAA1632	1	1	0	1	0	0	1	0	0	1	5
hsa-miR-708	KIAA1715	1	1	1	1	0	0	1	0	0	1	6
hsa-miR-708	KIAA1737	1	1	0	1	0	0	1	0	0	1	5
hsa-miR-708	KIAA1751	1	1	0	1	0	0	1	0	0	1	5
hsa-miR-708	KIF1A	1	1	0	0	0	0	1	0	0	1	4
hsa-miR-708	KIF1B	1	1	0	1	0	0	1	0	0	1	5
hsa-miR-708	KIF21B	1	1	0	1	0	0	1	0	0	0	4
hsa-miR-708	KIF3B	1	1	0	1	0	0	1	0	0	0	4
hsa-miR-708	KIF3C	1	1	0	1	0	0	1	0	0	1	5
hsa-miR-708	KIF6	1	1	0	1	0	0	1	0	0	1	5
hsa-miR-708	KL	1	1	0	1	0	0	1	0	0	0	4
hsa-miR-708	KLF17	1	1	0	1	0	0	1	0	0	1	5
hsa-miR-708	KLHDC3	1	1	0	1	0	0	1	0	0	0	4
hsa-miR-708	KLHDC8A	1	1	0	0	0	0	1	0	0	1	4
hsa-miR-708	KLHDC8B	1	1	0	1	0	0	1	0	0	0	4
hsa-miR-708	KLHL18	1	1	0	1	0	0	1	0	0	1	5
hsa-miR-708	KLHL21	1	1	0	1	0	0	1	0	0	1	5
hsa-miR-708	KLHL25	1	1	0	1	0	0	1	0	0	1	5

hsa-miR-708	KLHL26	1	1	0	1	0	0	1	0	0	0	4
hsa-miR-708	KLHL6	1	1	0	1	0	0	1	0	0	0	4
hsa-miR-708	KLK11	1	1	0	1	0	0	1	0	0	1	5
hsa-miR-708	KLK13	1	1	0	1	0	0	1	0	0	0	4
hsa-miR-708	KNDC1	1	1	0	1	0	0	1	0	0	1	5
hsa-miR-708	KPNA6	1	1	0	1	0	0	1	0	0	1	5
hsa-miR-708	KPNB1	1	1	0	0	0	0	1	0	0	1	4
hsa-miR-708	KREMEN1	1	1	0	1	0	0	1	0	0	1	5
hsa-miR-708	KRT1	1	1	0	1	0	0	1	0	0	1	5
hsa-miR-708	KRT2	1	1	0	1	0	0	1	0	0	1	5
hsa-miR-708	KRT32	1	1	0	1	0	0	1	0	0	0	4
hsa-miR-708	KRT6B	1	1	0	1	0	0	1	0	0	1	5
hsa-miR-708	KRT80	1	1	0	1	0	0	1	0	0	1	5
hsa-miR-708	KRT82	1	1	0	1	0	0	1	0	0	1	5
hsa-miR-708	KRTAP4-4	1	1	0	1	0	0	1	0	0	1	5
hsa-miR-708	KRTAP5-9	0	1	0	1	0	0	1	0	0	1	4
hsa-miR-708	KSR2	0	1	0	1	0	0	1	0	0	1	4
hsa-miR-708	KY	1	1	0	1	0	0	1	0	0	1	5
hsa-miR-708	L2HGDH	1	1	0	1	0	0	1	0	0	1	5
hsa-miR-708	LAD1	1	1	0	1	0	0	1	0	0	1	5
hsa-miR-708	LAIR1	1	1	0	1	0	0	1	0	0	1	5
hsa-miR-708	LAMP2	1	1	0	1	0	0	0	0	0	1	4
hsa-miR-708	LARP1	0	1	0	1	0	0	1	0	0	1	4
hsa-miR-708	LASP1	1	1	0	1	0	0	1	0	0	1	5
hsa-miR-708	LBH	1	1	0	1	0	0	1	0	0	1	5
hsa-miR-708	LCE3D	1	1	0	1	0	0	1	0	0	1	5
hsa-miR-708	LDB3	1	1	0	1	0	0	1	0	0	1	5
hsa-miR-708	LDLRAP1	1	1	0	1	0	0	1	0	0	1	5
hsa-miR-708	LDOC1	1	1	0	1	0	0	1	0	0	1	5
hsa-miR-708	LDOC1L	1	1	0	1	0	0	1	0	0	1	5
hsa-miR-708	LEMD2	1	1	0	1	0	0	1	0	0	1	5
hsa-miR-708	LEPREL2	1	0	0	1	0	0	1	0	0	1	4
hsa-miR-708	LETM1	1	1	0	1	0	0	1	0	0	0	4
hsa-miR-708	LGALS9	1	1	0	1	0	0	1	0	0	0	4
hsa-miR-708	LGI2	1	1	0	1	0	0	1	0	0	1	5
hsa-miR-708	LGI3	1	1	0	1	0	0	1	0	0	0	4
hsa-miR-708	LIF	1	1	0	1	0	0	1	0	0	1	5
hsa-miR-708	LIFR	1	1	0	1	0	0	0	0	0	1	4
hsa-miR-708	LIMD1	1	1	0	1	0	0	1	0	0	1	5
hsa-miR-708	LIMD2	1	1	0	1	0	0	1	0	0	1	5
hsa-miR-708	LIMK2	1	1	0	1	0	0	0	0	0	1	4
hsa-miR-708	LMBR1	1	1	0	1	0	0	1	0	0	1	5
hsa-miR-708	LMCD1	1	1	0	1	0	0	1	0	0	1	5
hsa-miR-708	LMOD3	0	1	0	1	0	0	1	0	0	1	4

hsa-miR-708	LOC129881	0	1	0	1	0	0	1	0	0	1	4
hsa-miR-708	LOC137886	1	1	0	1	0	0	1	0	0	1	5
hsa-miR-708	LOC150383	1	1	0	1	0	0	1	0	0	1	5
hsa-miR-708	LOC155006	1	1	0	1	0	0	1	0	0	1	5
hsa-miR-708	LOC196415	0	1	0	1	0	0	1	0	0	1	4
hsa-miR-708	LOC201164	1	1	0	1	0	0	1	0	0	0	4
hsa-miR-708	LOC283514	1	1	0	1	0	0	1	0	0	1	5
hsa-miR-708	LOC283871	1	1	0	1	0	0	1	0	0	0	4
hsa-miR-708	LOC285382	1	1	0	1	0	0	1	0	0	0	4
hsa-miR-708	LOC285636	1	1	0	1	0	0	1	0	0	0	4
hsa-miR-708	LOC388610	0	1	0	1	0	0	1	0	0	1	4
hsa-miR-708	LOC400120	1	1	0	1	0	0	1	0	0	1	5
hsa-miR-708	LOC400451	1	1	0	1	0	0	1	0	0	0	4
hsa-miR-708	LOC401052	0	1	0	1	0	0	1	0	0	1	4
hsa-miR-708	LOC63920	0	1	0	1	0	0	1	0	0	1	4
hsa-miR-708	LOC653319	1	1	0	1	0	0	1	0	0	1	5
hsa-miR-708	LOC729830	0	1	0	1	0	0	1	0	0	1	4
hsa-miR-708	LOC751071	1	1	0	1	0	0	1	0	0	1	5
hsa-miR-708	LONRF2	1	1	0	1	0	0	1	0	0	0	4
hsa-miR-708	LOXL2	1	1	0	0	0	0	1	0	0	1	4
hsa-miR-708	LOXL4	1	1	0	1	0	0	1	0	0	1	5
hsa-miR-708	LPHN1	1	1	1	1	0	0	1	0	0	1	6
hsa-miR-708	LPIN2	1	1	0	1	0	0	1	0	0	1	5
hsa-miR-708	LPIN3	1	1	0	1	0	0	1	0	0	0	4
hsa-miR-708	LPL	1	1	0	1	0	0	1	0	0	0	4
hsa-miR-708	LRIT1	1	1	0	1	0	0	1	0	0	0	4
hsa-miR-708	LRP1	1	1	0	1	0	0	1	0	0	1	5
hsa-miR-708	LRRC1	1	1	0	1	0	0	1	0	0	0	4
hsa-miR-708	LRRC14	1	1	0	1	0	0	1	0	0	0	4
hsa-miR-708	LRRC15	1	1	0	1	0	0	1	0	0	1	5
hsa-miR-708	LRRC25	1	1	0	1	0	0	1	0	0	1	5
hsa-miR-708	LRRC27	1	1	0	1	0	0	1	0	0	1	5
hsa-miR-708	LRRC59	1	1	0	1	0	0	1	0	0	1	5
hsa-miR-708	LRRC61	0	1	0	1	0	0	1	0	0	1	4
hsa-miR-708	LRRFIP2	1	1	0	1	0	0	1	0	0	0	4
hsa-miR-708	LRRK1	1	1	0	1	0	0	1	0	0	0	4
hsa-miR-708	LRRK2	1	1	0	0	0	0	1	0	0	1	4
hsa-miR-708	LRRTM1	1	1	0	1	0	0	1	0	0	0	4
hsa-miR-708	LRTM2	1	0	0	1	0	0	1	0	0	1	4
hsa-miR-708	LTBR	1	1	0	1	0	0	1	0	0	0	4
hsa-miR-708	LY86	1	1	0	1	0	0	1	0	0	1	5
hsa-miR-708	LYK5	1	1	0	1	0	0	1	0	0	0	4
hsa-miR-708	LYPD3	1	1	0	1	0	0	1	0	0	1	5
hsa-miR-708	LYPLA3	1	1	0	1	0	0	1	0	0	1	5

hsa-miR-708	LYRM2	0	1	0	1	0	0	1	0	0	1	4
hsa-miR-708	LYVE1	1	1	0	1	0	0	1	0	0	1	5
hsa-miR-708	LZTR1	1	1	0	1	0	0	1	0	0	1	5
hsa-miR-708	MACROD2	1	1	0	1	0	0	1	0	0	1	5
hsa-miR-708	MAD2L1	1	1	0	1	0	0	1	0	0	1	5
hsa-miR-708	MAFF	1	0	0	1	0	0	1	0	0	1	4
hsa-miR-708	MAFG	1	0	0	1	0	0	1	0	0	1	4
hsa-miR-708	MAFK	1	1	0	1	0	0	1	0	0	0	4
hsa-miR-708	MAML1	1	1	0	1	0	0	1	0	0	1	5
hsa-miR-708	MAML3	0	1	0	1	0	0	1	0	0	1	4
hsa-miR-708	MANEAL	1	1	0	1	0	0	0	0	0	1	4
hsa-miR-708	MAP1A	1	1	0	1	0	0	1	0	0	1	5
hsa-miR-708	MAP1B	1	1	0	1	0	0	1	0	0	1	5
hsa-miR-708	MAP1LC3A	1	1	0	1	0	0	1	0	0	1	5
hsa-miR-708	MAP3K2	1	1	0	1	0	0	1	0	0	1	5
hsa-miR-708	MAP7	1	1	0	1	0	0	1	0	0	1	5
hsa-miR-708	MAPK1	1	1	0	1	0	0	1	0	0	1	5
hsa-miR-708	MAPK4	1	1	0	1	0	0	1	0	0	1	5
hsa-miR-708	MAPK8IP3	1	1	0	1	0	0	1	0	0	1	5
hsa-miR-708	MAPRE1	1	1	0	1	0	0	1	0	0	0	4
hsa-miR-708	MARK4	1	1	0	1	0	0	1	0	0	1	5
hsa-miR-708	MASP1	1	1	0	1	0	0	1	0	0	1	5
hsa-miR-708	MAT1A	1	1	0	1	0	0	1	0	0	1	5
hsa-miR-708	MAT2A	1	1	0	1	0	0	1	0	0	1	5
hsa-miR-708	MAX	1	1	0	1	0	0	1	0	0	1	5
hsa-miR-708	MAZ	1	1	0	1	0	0	1	0	0	1	5
hsa-miR-708	MBD3	1	1	0	1	0	0	1	0	0	1	5
hsa-miR-708	MBP	1	1	0	1	0	0	1	0	0	0	4
hsa-miR-708	MCART6	1	1	0	1	0	0	1	0	0	0	4
hsa-miR-708	MCAT	1	1	0	1	0	0	1	0	0	1	5
hsa-miR-708	MCC	1	1	0	1	0	0	1	0	0	1	5
hsa-miR-708	MCM9	0	1	0	1	0	0	1	0	0	1	4
hsa-miR-708	MDGA1	1	1	0	1	0	0	1	0	0	1	5
hsa-miR-708	MED19	1	1	0	1	0	0	1	0	0	1	5
hsa-miR-708	MED22	1	1	0	1	0	0	1	0	0	1	5
hsa-miR-708	MED24	1	1	0	1	0	0	1	0	0	1	5
hsa-miR-708	MED29	1	1	0	1	0	0	1	0	0	1	5
hsa-miR-708	MED9	1	1	0	1	0	0	1	0	0	1	5
hsa-miR-708	MEF2C	1	1	0	1	0	0	1	0	0	0	4
hsa-miR-708	MEF2D	1	1	0	1	0	0	1	0	0	1	5
hsa-miR-708	MEGF6	1	1	0	0	0	0	1	0	0	1	4
hsa-miR-708	MEGF8	0	1	0	1	0	0	1	0	0	1	4
hsa-miR-708	MEN1	1	1	0	1	0	0	0	0	0	1	4
hsa-miR-708	MEOX2	1	1	0	1	0	0	1	0	0	1	5

hsa-miR-708	MERTK	1	1	0	1	0	0	1	0	0	0	4
hsa-miR-708	MESDC2	1	1	0	1	0	0	1	0	0	1	5
hsa-miR-708	MEST	1	1	0	1	0	0	1	0	0	1	5
hsa-miR-708	METAP2	1	1	0	1	0	0	1	0	0	1	5
hsa-miR-708	METT10D	1	1	0	1	0	0	1	0	0	1	5
hsa-miR-708	METTL7B	1	1	0	1	0	0	1	0	0	1	5
hsa-miR-708	METTL8	1	1	0	1	0	0	1	0	0	1	5
hsa-miR-708	MFAP3L	1	1	0	1	0	0	1	0	0	1	5
hsa-miR-708	MFNG	1	1	0	1	0	0	1	0	0	1	5
hsa-miR-708	MFRP	0	1	0	1	0	0	1	0	0	1	4
hsa-miR-708	MFSD10	1	0	0	1	0	0	1	0	0	1	4
hsa-miR-708	MFSD4	1	1	0	1	0	0	1	0	0	1	5
hsa-miR-708	MFSD7	1	1	0	1	0	0	1	0	0	0	4
hsa-miR-708	MGC13057	1	0	0	1	0	0	1	0	0	1	4
hsa-miR-708	MGC13379	0	1	0	1	0	0	1	0	0	1	4
hsa-miR-708	MGC24039	1	1	0	1	0	0	1	0	0	1	5
hsa-miR-708	MGC4655	0	1	0	1	0	0	1	0	0	1	4
hsa-miR-708	MGLL	1	1	0	1	0	0	0	0	0	1	4
hsa-miR-708	MICALL1	1	1	0	1	0	0	1	0	0	1	5
hsa-miR-708	MIOX	1	1	0	1	0	0	1	0	0	1	5
hsa-miR-708	MIS12	1	1	0	1	0	0	1	0	0	0	4
hsa-miR-708	MKL1	1	1	0	1	0	0	1	0	0	0	4
hsa-miR-708	MKLN1	1	1	0	0	0	0	1	0	0	1	4
hsa-miR-708	MLC1	1	0	0	1	0	0	1	0	0	1	4
hsa-miR-708	MLKL	1	1	0	1	0	0	1	0	0	1	5
hsa-miR-708	MLL	1	1	0	1	0	0	1	0	0	1	5
hsa-miR-708	MLLT6	1	1	0	0	0	0	1	0	0	1	4
hsa-miR-708	MLX	1	1	0	0	0	0	1	0	0	1	4
hsa-miR-708	MMP14	1	1	0	1	0	0	1	0	0	0	4
hsa-miR-708	MMP15	1	1	0	1	0	0	1	0	0	1	5
hsa-miR-708	MMP19	1	1	0	1	0	0	1	0	0	1	5
hsa-miR-708	MOBKL1A	1	1	0	1	0	0	1	0	0	0	4
hsa-miR-708	MOBKL2B	1	1	1	0	0	0	1	0	0	1	5
hsa-miR-708	MOBP	1	1	0	1	0	0	1	0	0	1	5
hsa-miR-708	MOCS1	1	1	0	1	0	0	1	0	0	0	4
hsa-miR-708	MOG	1	1	0	1	0	0	0	0	0	1	4
hsa-miR-708	MORF4L1	1	1	0	1	0	0	1	0	0	1	5
hsa-miR-708	MORF4L2	1	0	1	1	0	0	1	0	0	1	5
hsa-miR-708	MOSC1	1	1	0	1	0	0	1	0	0	0	4
hsa-miR-708	MPL	1	1	0	1	0	0	1	0	0	1	5
hsa-miR-708	MPP2	1	1	0	1	0	0	1	0	0	1	5
hsa-miR-708	MRC2	1	1	0	1	0	0	1	0	0	1	5
hsa-miR-708	MRE11A	1	0	1	1	0	0	1	0	0	1	5
hsa-miR-708	MRGPRF	1	1	0	1	0	0	0	0	0	1	4

hsa-miR-708	MRPL11	1	1	0	1	0	0	0	0	0	1	4
hsa-miR-708	MRPL17	1	1	0	1	0	0	0	0	0	1	4
hsa-miR-708	MRPL28	0	1	0	1	0	0	1	0	0	1	4
hsa-miR-708	MRPL35	1	1	0	1	0	0	1	0	0	1	5
hsa-miR-708	MRPL4	1	1	0	1	0	0	1	0	0	0	4
hsa-miR-708	MS4A1	1	1	0	1	0	0	0	0	0	1	4
hsa-miR-708	MSL2L1	1	1	0	1	0	0	1	0	0	1	5
hsa-miR-708	MSL3L1	1	1	0	1	0	0	1	0	0	1	5
hsa-miR-708	MSN	1	1	0	1	0	0	1	0	0	1	5
hsa-miR-708	MST150	1	1	0	0	0	0	1	0	0	1	4
hsa-miR-708	MTCP1	1	1	1	1	0	0	1	0	0	1	6
hsa-miR-708	MTF1	1	1	0	1	0	0	1	0	0	1	5
hsa-miR-708	MTHFR	1	1	0	1	0	0	1	0	0	1	5
hsa-miR-708	MTMR3	1	1	0	1	0	0	1	0	0	1	5
hsa-miR-708	MTSS1	1	1	1	1	0	0	1	0	0	1	6
hsa-miR-708	MUM1L1	1	1	0	1	0	0	1	0	0	1	5
hsa-miR-708	MVK	1	0	0	1	0	0	1	0	0	1	4
hsa-miR-708	MXD1	1	1	0	1	0	0	1	0	0	0	4
hsa-miR-708	MXD4	1	1	0	1	0	0	1	0	0	1	5
hsa-miR-708	MYCL1	1	0	0	1	0	0	1	0	0	1	4
hsa-miR-708	MYEOV	1	1	0	1	0	0	1	0	0	1	5
hsa-miR-708	MYH15	1	1	0	1	0	0	1	0	0	1	5
hsa-miR-708	MYH7	1	1	0	1	0	0	1	0	0	1	5
hsa-miR-708	MYLK2	1	1	0	1	0	0	1	0	0	1	5
hsa-miR-708	MYO16	1	1	0	1	0	0	1	0	0	1	5
hsa-miR-708	MYO1C	0	1	0	1	0	0	1	0	0	1	4
hsa-miR-708	MYO5A	1	1	0	1	0	0	1	0	0	1	5
hsa-miR-708	MYOC	1	1	0	1	0	0	1	0	0	1	5
hsa-miR-708	MYST3	1	0	0	1	0	0	1	0	0	1	4
hsa-miR-708	N4BP1	1	1	0	0	0	0	1	0	0	1	4
hsa-miR-708	N4BP3	1	1	0	1	0	0	1	0	0	1	5
hsa-miR-708	N6AMT1	1	1	0	1	0	0	1	0	0	0	4
hsa-miR-708	NAB2	1	1	0	1	0	0	1	0	0	0	4
hsa-miR-708	NAGPA	1	1	0	1	0	0	1	0	0	1	5
hsa-miR-708	NANOS1	1	1	0	1	0	0	1	0	0	1	5
hsa-miR-708	NAPA	1	1	0	1	0	0	1	0	0	0	4
hsa-miR-708	NAT14	1	1	0	1	0	0	1	0	0	1	5
hsa-miR-708	NAT9	1	1	0	1	0	0	1	0	0	1	5
hsa-miR-708	NAV1	1	1	0	1	0	0	1	0	0	1	5
hsa-miR-708	NBPF3	1	1	0	1	0	0	1	0	0	0	4
hsa-miR-708	NCAM1	1	1	0	1	0	0	1	0	0	1	5
hsa-miR-708	NCAN	1	1	0	1	0	0	1	0	0	1	5
hsa-miR-708	NCAPD3	1	1	0	1	0	0	1	0	0	1	5
hsa-miR-708	NCLN	1	1	0	1	0	0	1	0	0	0	4

hsa-miR-708	NCOA5	1	1	0	1	0	0	1	0	0	1	5
hsa-miR-708	NDE1	1	1	0	1	0	0	1	0	0	0	4
hsa-miR-708	NDRG2	1	0	0	1	0	0	1	0	0	1	4
hsa-miR-708	NDRG3	1	1	0	1	0	0	1	0	0	0	4
hsa-miR-708	NDST1	1	1	0	1	0	0	1	0	0	1	5
hsa-miR-708	NECAP2	1	1	0	1	0	0	1	0	0	1	5
hsa-miR-708	NELL1	1	1	0	1	0	0	1	0	0	1	5
hsa-miR-708	NETO1	0	1	0	1	0	0	1	0	0	1	4
hsa-miR-708	NEU3	1	1	0	1	0	0	1	0	0	1	5
hsa-miR-708	NEURL	1	1	0	1	0	0	1	0	0	1	5
hsa-miR-708	NF2	1	1	0	1	0	0	1	0	0	0	4
hsa-miR-708	NFASC	1	0	0	1	0	0	1	0	0	1	4
hsa-miR-708	NFAT5	1	1	0	1	0	0	0	0	0	1	4
hsa-miR-708	NFE2L1	1	1	0	1	0	0	1	0	0	0	4
hsa-miR-708	NFE2L2	1	1	1	1	0	0	1	0	0	1	6
hsa-miR-708	NFIX	1	1	0	1	0	0	1	0	0	0	4
hsa-miR-708	NGB	1	1	0	1	0	0	1	0	0	1	5
hsa-miR-708	NGDN	1	1	0	1	0	0	1	0	0	0	4
hsa-miR-708	NGFR	1	1	0	1	0	0	1	0	0	1	5
hsa-miR-708	NHLH1	1	1	0	0	0	0	1	0	0	1	4
hsa-miR-708	NICN1	1	1	0	1	0	0	1	0	0	1	5
hsa-miR-708	NID1	1	1	0	1	0	0	1	0	0	1	5
hsa-miR-708	NIP30	1	1	0	1	0	0	1	0	0	1	5
hsa-miR-708	NKX2-3	0	1	0	1	0	0	1	0	0	1	4
hsa-miR-708	NLGN2	1	1	0	1	0	0	1	0	0	1	5
hsa-miR-708	NLRC3	1	1	0	1	0	0	1	0	0	1	5
hsa-miR-708	NMNAT3	1	1	0	1	0	0	1	0	0	1	5
hsa-miR-708	NMT1	1	1	0	1	0	0	1	0	0	1	5
hsa-miR-708	NMT2	1	1	0	1	0	0	1	0	0	0	4
hsa-miR-708	NNAT	1	1	0	1	0	0	1	0	0	1	5
hsa-miR-708	NOL12	0	1	0	1	0	0	1	0	0	1	4
hsa-miR-708	NOPE	1	1	0	1	0	0	1	0	0	1	5
hsa-miR-708	NOS1	1	1	0	1	0	0	1	0	0	1	5
hsa-miR-708	NOS1AP	1	1	0	1	0	0	0	0	0	1	4
hsa-miR-708	NOTCH1	1	1	0	1	0	0	1	0	0	1	5
hsa-miR-708	N-PAC	1	1	0	1	0	0	1	0	0	1	5
hsa-miR-708	NPNT	1	1	0	1	0	0	1	0	0	0	4
hsa-miR-708	NPY2R	1	1	1	1	0	0	1	0	0	1	6
hsa-miR-708	NR1H4	1	1	0	1	0	0	1	0	0	1	5
hsa-miR-708	NR2C2	1	1	0	1	0	0	1	0	0	0	4
hsa-miR-708	NR3C2	1	1	0	1	0	0	1	0	0	1	5
hsa-miR-708	NR4A3	0	0	1	1	0	0	1	0	0	1	4
hsa-miR-708	NRAS	1	1	1	1	0	0	1	0	0	1	6
hsa-miR-708	NRBP2	1	0	0	1	0	0	1	0	0	1	4

hsa-miR-708	NRF1	1	1	0	1	0	0	1	0	0	1	5
hsa-miR-708	NRIP3	1	1	0	1	0	0	1	0	0	0	4
hsa-miR-708	NRSN2	1	1	0	0	0	0	1	0	0	1	4
hsa-miR-708	NSFL1C	1	1	0	1	0	0	1	0	0	0	4
hsa-miR-708	NT5C3	0	0	1	1	0	0	1	0	0	1	4
hsa-miR-708	NT5DC3	1	1	0	1	0	0	0	0	0	1	4
hsa-miR-708	NT5E	1	1	0	1	0	0	1	0	0	1	5
hsa-miR-708	NTN1	0	1	0	1	0	0	1	0	0	1	4
hsa-miR-708	NTRK2	1	0	0	1	0	0	1	0	0	1	4
hsa-miR-708	NTRK3	1	1	0	1	0	0	1	0	0	1	5
hsa-miR-708	NUAK1	1	1	0	1	0	0	1	0	0	1	5
hsa-miR-708	NUAK2	1	1	0	1	0	0	1	0	0	1	5
hsa-miR-708	NUDCD3	1	1	0	1	0	0	1	0	0	1	5
hsa-miR-708	NUDT11	1	1	0	1	0	0	1	0	0	1	5
hsa-miR-708	NUFIP1	1	1	0	1	0	0	1	0	0	1	5
hsa-miR-708	NUP210	1	1	0	1	0	0	1	0	0	1	5
hsa-miR-708	NUP98	1	1	0	1	0	0	1	0	0	1	5
hsa-miR-708	NUPL1	0	1	0	1	0	0	1	0	0	1	4
hsa-miR-708	NXF1	0	1	0	1	0	0	1	0	0	1	4
hsa-miR-708	NXF3	1	1	0	1	0	0	1	0	0	1	5
hsa-miR-708	OAS1	1	1	0	1	0	0	1	0	0	0	4
hsa-miR-708	OAS3	0	1	0	1	0	0	1	0	0	1	4
hsa-miR-708	OCRL	1	1	0	0	0	0	1	0	0	1	4
hsa-miR-708	ODZ3	0	1	0	1	0	0	1	0	0	1	4
hsa-miR-708	ODZ4	1	1	0	1	0	0	1	0	0	1	5
hsa-miR-708	OGDH	1	1	0	0	0	0	1	0	0	1	4
hsa-miR-708	OGDHL	1	1	0	1	0	0	1	0	0	1	5
hsa-miR-708	OLA1	1	1	0	1	0	0	1	0	0	1	5
hsa-miR-708	OLFM1	1	1	0	0	0	0	1	0	0	1	4
hsa-miR-708	OLFM4	1	1	0	0	0	0	1	0	0	1	4
hsa-miR-708	OLFML2A	1	1	0	1	0	0	1	0	0	1	5
hsa-miR-708	OLFML3	1	1	0	1	0	0	1	0	0	1	5
hsa-miR-708	OPA3	0	1	0	1	0	0	1	0	0	1	4
hsa-miR-708	OPN4	1	1	0	1	0	0	1	0	0	1	5
hsa-miR-708	OPN5	1	1	0	1	0	0	1	0	0	1	5
hsa-miR-708	OR11A1	1	1	0	1	0	0	0	0	0	1	4
hsa-miR-708	ORC6L	1	1	0	1	0	0	1	0	0	1	5
hsa-miR-708	OSBPL10	1	1	0	1	0	0	1	0	0	1	5
hsa-miR-708	OSBPL2	1	1	0	1	0	0	1	0	0	0	4
hsa-miR-708	OSBPL3	1	1	1	1	0	0	1	0	0	1	6
hsa-miR-708	OSBPL5	0	1	0	1	0	0	1	0	0	1	4
hsa-miR-708	OSBPL7	1	1	0	1	0	0	1	0	0	1	5
hsa-miR-708	OTOF	1	1	0	1	0	0	1	0	0	1	5
hsa-miR-708	OTP	1	1	0	0	0	0	1	0	0	1	4

hsa-miR-708	OTUB1	1	1	0	1	0	0	1	0	0	1	5
hsa-miR-708	OTUD4	1	1	0	1	0	0	1	0	0	1	5
hsa-miR-708	OTUD7B	1	1	0	0	0	0	1	0	0	1	4
hsa-miR-708	OVOL1	1	1	0	1	0	0	1	0	0	1	5
hsa-miR-708	OXGR1	1	1	0	1	0	0	1	0	0	1	5
hsa-miR-708	OXNAD1	1	1	0	1	0	0	1	0	0	1	5
hsa-miR-708	P15RS	1	1	1	1	0	0	1	0	0	1	6
hsa-miR-708	P2RX1	1	1	1	1	0	0	1	0	0	1	6
hsa-miR-708	P2RX4	1	1	0	1	0	0	1	0	0	1	5
hsa-miR-708	P4HA1	1	1	0	1	0	0	1	0	0	1	5
hsa-miR-708	PABPC5	1	1	0	1	0	0	1	0	0	0	4
hsa-miR-708	PACS1	1	1	0	1	0	0	1	0	0	0	4
hsa-miR-708	PACS2	1	1	0	1	0	0	1	0	0	1	5
hsa-miR-708	PACSIN1	1	1	0	0	0	0	1	0	0	1	4
hsa-miR-708	PADI1	1	1	0	1	0	0	1	0	0	1	5
hsa-miR-708	PADI2	1	1	0	1	0	0	1	0	0	1	5
hsa-miR-708	PADI3	1	1	0	1	0	0	1	0	0	1	5
hsa-miR-708	PAFAH2	1	1	0	1	0	0	1	0	0	1	5
hsa-miR-708	PAG1	1	1	0	1	0	0	1	0	0	1	5
hsa-miR-708	PAK2	1	1	1	1	0	0	1	0	0	1	6
hsa-miR-708	PAN2	1	1	0	1	0	0	0	0	0	1	4
hsa-miR-708	PAP2D	1	1	0	1	0	0	1	0	0	1	5
hsa-miR-708	PAPD5	1	1	0	1	0	0	1	0	0	1	5
hsa-miR-708	PAPLN	1	1	0	1	0	0	1	0	0	0	4
hsa-miR-708	PAPOLA	1	1	0	1	0	0	1	0	0	1	5
hsa-miR-708	PAPPA	1	1	0	1	0	0	1	0	0	1	5
hsa-miR-708	PAQR4	1	1	1	1	0	0	1	0	0	1	6
hsa-miR-708	PAQR5	1	0	0	1	0	0	1	0	0	1	4
hsa-miR-708	PAQR7	1	1	0	1	0	0	1	0	0	1	5
hsa-miR-708	PARK2	1	1	0	1	0	0	1	0	0	1	5
hsa-miR-708	PARP16	1	1	0	1	0	0	1	0	0	0	4
hsa-miR-708	PAX3	1	1	0	1	0	0	1	0	0	0	4
hsa-miR-708	PAX4	1	1	0	1	0	0	1	0	0	0	4
hsa-miR-708	PAX8	1	1	0	1	0	0	1	0	0	1	5
hsa-miR-708	PBK	1	1	0	1	0	0	1	0	0	1	5
hsa-miR-708	PCDH1	1	1	0	1	0	0	1	0	0	0	4
hsa-miR-708	PCDH20	1	1	0	1	0	0	1	0	0	0	4
hsa-miR-708	PCDH21	1	1	0	1	0	0	1	0	0	0	4
hsa-miR-708	PCDHA6	1	1	0	1	0	0	1	0	0	0	4
hsa-miR-708	PCDHA9	1	1	0	1	0	0	1	0	0	1	5
hsa-miR-708	PCGF3	1	1	0	1	0	0	1	0	0	1	5
hsa-miR-708	PCGF5	1	1	0	1	0	0	1	0	0	1	5
hsa-miR-708	PCNX	0	1	0	1	0	0	1	0	0	1	4
hsa-miR-708	PCSK7	1	1	0	1	0	0	1	0	0	0	4

hsa-miR-708	PCYOX1	1	1	0	1	0	0	1	0	0	1	5
hsa-miR-708	PCYOX1L	1	1	0	1	0	0	1	0	0	1	5
hsa-miR-708	PCYT1B	1	1	0	1	0	0	1	0	0	1	5
hsa-miR-708	PDCD1	1	1	0	1	0	0	1	0	0	1	5
hsa-miR-708	PDCD1LG2	0	1	0	1	0	0	1	0	0	1	4
hsa-miR-708	PDE1B	1	1	0	1	0	0	1	0	0	1	5
hsa-miR-708	PDE2A	1	1	0	1	0	0	1	0	0	0	4
hsa-miR-708	PDE8A	1	1	0	1	0	0	1	0	0	1	5
hsa-miR-708	PDGFB	1	1	0	1	0	0	1	0	0	0	4
hsa-miR-708	PDK4	1	1	1	0	0	0	1	0	0	1	5
hsa-miR-708	PDLIM4	1	1	0	1	0	0	1	0	0	1	5
hsa-miR-708	PDPR	1	1	0	1	0	0	1	0	0	1	5
hsa-miR-708	PDZD4	1	1	0	1	0	0	1	0	0	1	5
hsa-miR-708	PELI1	1	1	0	1	0	0	1	0	0	0	4
hsa-miR-708	PEX11G	1	1	0	0	0	0	1	0	0	1	4
hsa-miR-708	PEX12	1	1	0	1	0	0	1	0	0	1	5
hsa-miR-708	PFDN1	1	1	0	1	0	0	1	0	0	1	5
hsa-miR-708	PFKM	1	1	0	1	0	0	1	0	0	1	5
hsa-miR-708	PFTK1	1	1	0	0	0	0	1	0	0	1	4
hsa-miR-708	PGF	1	1	0	0	0	0	1	0	0	1	4
hsa-miR-708	PGPEP1	1	1	0	1	0	0	1	0	0	1	5
hsa-miR-708	PHCA	1	1	0	1	0	0	1	0	0	1	5
hsa-miR-708	PHF15	1	1	0	1	0	0	1	0	0	1	5
hsa-miR-708	PHF19	1	1	0	1	0	0	1	0	0	1	5
hsa-miR-708	PHF21A	0	1	0	1	0	0	1	0	0	1	4
hsa-miR-708	PHF6	1	1	0	1	0	0	1	0	0	1	5
hsa-miR-708	PHKB	1	1	0	1	0	0	0	0	0	1	4
hsa-miR-708	PHKG2	1	1	0	1	0	0	1	0	0	1	5
hsa-miR-708	PHLDA1	1	1	0	1	0	0	1	0	0	1	5
hsa-miR-708	PHLPPL	1	1	0	1	0	0	1	0	0	1	5
hsa-miR-708	PHOX2A	1	1	0	1	0	0	1	0	0	1	5
hsa-miR-708	PHYHIP	1	0	0	1	0	0	1	0	0	1	4
hsa-miR-708	PI15	1	1	0	1	0	0	1	0	0	1	5
hsa-miR-708	PIGR	1	1	0	1	0	0	1	0	0	1	5
hsa-miR-708	PIGZ	1	1	0	1	0	0	1	0	0	1	5
hsa-miR-708	PIK3IP1	1	1	0	1	0	0	1	0	0	1	5
hsa-miR-708	PIK3R3	1	1	0	1	0	0	1	0	0	1	5
hsa-miR-708	PIN4	1	1	0	1	0	0	1	0	0	0	4
hsa-miR-708	PIP4K2C	1	1	0	1	0	0	1	0	0	0	4
hsa-miR-708	PIP5K1A	1	0	0	1	0	0	1	0	0	1	4
hsa-miR-708	PITPNM2	1	1	0	1	0	0	1	0	0	0	4
hsa-miR-708	PITX2	1	1	0	1	0	0	1	0	0	1	5
hsa-miR-708	PKD1L2	1	1	0	1	0	0	0	0	0	1	4
hsa-miR-708	PKMYT1	1	1	0	1	0	0	1	0	0	0	4

hsa-miR-708	PKN2	1	1	0	1	0	0	1	0	0	0	4
hsa-miR-708	PKNOX1	1	1	0	1	0	0	1	0	0	1	5
hsa-miR-708	PKNOX2	1	1	0	1	0	0	1	0	0	1	5
hsa-miR-708	PLA2G2F	1	1	0	1	0	0	1	0	0	1	5
hsa-miR-708	PLA2G4C	1	1	0	1	0	0	1	0	0	0	4
hsa-miR-708	PLA2G4F	1	1	0	1	0	0	1	0	0	1	5
hsa-miR-708	PLA2G5	0	1	0	1	0	0	1	0	0	1	4
hsa-miR-708	PLAG1	1	1	0	1	0	0	0	0	0	1	4
hsa-miR-708	PLCD3	1	1	0	1	0	0	1	0	0	1	5
hsa-miR-708	PLD2	1	1	0	0	0	0	1	0	0	1	4
hsa-miR-708	PLD5	1	1	0	1	0	0	1	0	0	0	4
hsa-miR-708	PLEK	1	1	0	1	0	0	1	0	0	1	5
hsa-miR-708	PLEKHA2	0	1	0	1	0	0	1	0	0	1	4
hsa-miR-708	PLEKHA6	1	1	0	1	0	0	1	0	0	1	5
hsa-miR-708	PLEKHA7	1	1	0	1	0	0	1	0	0	1	5
hsa-miR-708	PLEKHB1	1	1	0	1	0	0	0	0	0	1	4
hsa-miR-708	PLEKHG4B	1	1	0	1	0	0	1	0	0	0	4
hsa-miR-708	PLEKHH1	1	1	0	0	0	0	1	0	0	1	4
hsa-miR-708	PLEKHN1	1	1	0	1	0	0	1	0	0	1	5
hsa-miR-708	PLK3	1	1	0	0	0	0	1	0	0	1	4
hsa-miR-708	PLVAP	1	1	0	1	0	0	1	0	0	0	4
hsa-miR-708	PLXDC1	1	1	0	1	0	0	1	0	0	1	5
hsa-miR-708	PLXNA1	1	1	0	1	0	0	1	0	0	1	5
hsa-miR-708	PLXNA2	1	1	0	1	0	0	1	0	0	1	5
hsa-miR-708	PLXNA3	1	1	0	1	0	0	1	0	0	0	4
hsa-miR-708	PLXNA4	0	1	0	1	0	0	1	0	0	1	4
hsa-miR-708	PMAIP1	1	1	0	1	0	0	1	0	0	0	4
hsa-miR-708	PML	1	1	0	1	0	0	1	0	0	1	5
hsa-miR-708	PNMA3	1	1	0	0	0	0	1	0	0	1	4
hsa-miR-708	PNMA6A	0	1	0	1	0	0	1	0	0	1	4
hsa-miR-708	PNMAL1	1	1	0	1	0	0	1	0	0	1	5
hsa-miR-708	PNMAL2	0	1	0	1	0	0	1	0	0	1	4
hsa-miR-708	PODXL2	1	1	0	1	0	0	1	0	0	0	4
hsa-miR-708	POFUT1	1	1	0	1	0	0	1	0	0	1	5
hsa-miR-708	POFUT2	1	1	0	1	0	0	1	0	0	0	4
hsa-miR-708	POLA1	1	1	0	1	0	0	1	0	0	1	5
hsa-miR-708	POLR1D	1	1	0	1	0	0	1	0	0	0	4
hsa-miR-708	POU2AF1	1	1	0	1	0	0	1	0	0	0	4
hsa-miR-708	POU6F1	1	1	0	0	0	0	1	0	0	1	4
hsa-miR-708	PPARA	1	1	0	1	0	0	0	0	0	1	4
hsa-miR-708	PPARD	1	1	0	1	0	0	1	0	0	1	5
hsa-miR-708	PPFIA1	1	1	1	1	0	0	1	0	0	1	6
hsa-miR-708	PPFIA4	1	0	0	1	0	0	1	0	0	1	4
hsa-miR-708	PPIE	1	1	0	0	0	0	1	0	0	1	4

hsa-miR-708	PPIF	1	1	0	1	0	0	1	0	0	1	5
hsa-miR-708	PPIL2	1	1	0	1	0	0	1	0	0	1	5
hsa-miR-708	PPM1E	1	1	0	1	0	0	1	0	0	1	5
hsa-miR-708	PPM1F	1	1	0	1	0	0	1	0	0	1	5
hsa-miR-708	PPM1H	0	1	0	1	0	0	1	0	0	1	4
hsa-miR-708	PPME1	0	1	0	1	0	0	1	0	0	1	4
hsa-miR-708	PPP1R12B	0	1	0	1	0	0	1	0	0	1	4
hsa-miR-708	PPP1R14D	1	1	0	1	0	0	0	0	0	1	4
hsa-miR-708	PPP1R16B	1	1	0	1	0	0	1	0	0	1	5
hsa-miR-708	PPP1R3A	1	1	0	1	0	0	1	0	0	0	4
hsa-miR-708	PPP2R2C	1	0	0	1	0	0	1	0	0	1	4
hsa-miR-708	PPP3R2	1	1	0	1	0	0	1	0	0	1	5
hsa-miR-708	PRCC	1	1	0	1	0	0	1	0	0	1	5
hsa-miR-708	PRDM16	1	1	0	1	0	0	1	0	0	0	4
hsa-miR-708	PRDM2	1	1	0	1	0	0	1	0	0	1	5
hsa-miR-708	PRELP	1	0	0	1	0	0	1	0	0	1	4
hsa-miR-708	PREPL	1	1	1	1	0	0	1	0	0	1	6
hsa-miR-708	PRICKLE2	1	1	0	1	0	0	1	0	0	1	5
hsa-miR-708	PRKAA1	1	1	0	1	0	0	0	0	0	1	4
hsa-miR-708	PRKAB1	1	1	0	1	0	0	1	0	0	1	5
hsa-miR-708	PRKAG3	1	1	0	1	0	0	1	0	0	1	5
hsa-miR-708	PRKAR2A	1	1	0	1	0	0	1	0	0	0	4
hsa-miR-708	PRKCI	1	1	0	1	0	0	1	0	0	1	5
hsa-miR-708	PRKDC	1	1	0	1	0	0	1	0	0	0	4
hsa-miR-708	PRKRIP1	1	1	0	1	0	0	1	0	0	1	5
hsa-miR-708	PRLR	1	1	0	0	0	0	1	0	0	1	4
hsa-miR-708	PRMT8	1	1	0	1	0	0	1	0	0	1	5
hsa-miR-708	PROCR	1	1	0	1	0	0	1	0	0	1	5
hsa-miR-708	PRR18	0	1	0	1	0	0	1	0	0	1	4
hsa-miR-708	PRRX1	1	1	0	1	0	0	1	0	0	0	4
hsa-miR-708	PRSS16	1	1	0	1	0	0	1	0	0	1	5
hsa-miR-708	PRSS23	1	1	0	1	0	0	1	0	0	0	4
hsa-miR-708	PRSS27	1	1	0	1	0	0	1	0	0	0	4
hsa-miR-708	PRSS8	1	1	0	1	0	0	1	0	0	1	5
hsa-miR-708	PSAP	1	0	0	1	0	0	1	0	0	1	4
hsa-miR-708	PSCD1	1	0	0	1	0	0	1	0	0	1	4
hsa-miR-708	PSCD2	0	1	0	1	0	0	1	0	0	1	4
hsa-miR-708	PSCDBP	1	1	0	1	0	0	1	0	0	1	5
hsa-miR-708	PSD2	1	1	0	1	0	0	1	0	0	1	5
hsa-miR-708	PSD3	1	1	0	0	0	0	1	0	0	1	4
hsa-miR-708	PSD4	1	1	0	1	0	0	1	0	0	0	4
hsa-miR-708	PSENEN	1	1	0	1	0	0	1	0	0	0	4
hsa-miR-708	PSG2	0	1	1	1	0	0	1	0	0	1	5
hsa-miR-708	PSMA5	1	1	0	1	0	0	1	0	0	1	5

hsa-miR-708	PSMD9	1	1	0	1	0	0	1	0	0	1	5
hsa-miR-708	PSME3	1	1	0	1	0	0	0	0	0	1	4
hsa-miR-708	PSMF1	1	1	0	0	0	0	1	0	0	1	4
hsa-miR-708	PSTPIP2	1	1	0	1	0	0	1	0	0	0	4
hsa-miR-708	PTCH1	1	1	0	1	0	0	0	0	0	1	4
hsa-miR-708	PTEN	1	1	0	1	0	0	1	0	0	1	5
hsa-miR-708	PTGER3	1	1	1	1	0	0	1	0	0	1	6
hsa-miR-708	PTGFRN	1	1	0	0	0	0	1	0	0	1	4
hsa-miR-708	PTGS2	1	1	0	1	0	0	1	0	0	1	5
hsa-miR-708	PTPN21	0	1	1	1	0	0	1	0	0	1	5
hsa-miR-708	PTPN3	1	1	0	1	0	0	1	0	0	1	5
hsa-miR-708	PTPN7	1	1	0	1	0	0	0	0	0	1	4
hsa-miR-708	PTPRB	1	1	0	0	0	0	1	0	0	1	4
hsa-miR-708	PTPRF	1	1	0	1	0	0	1	0	0	1	5
hsa-miR-708	PTPRJ	0	1	0	1	0	0	1	0	0	1	4
hsa-miR-708	PTPRN2	1	1	0	1	0	0	1	0	0	1	5
hsa-miR-708	PTPRR	1	1	0	1	0	0	1	0	0	1	5
hsa-miR-708	PTPRT	1	1	0	0	0	0	1	0	0	1	4
hsa-miR-708	PTPRU	1	1	0	1	0	0	1	0	0	1	5
hsa-miR-708	PTRF	1	1	0	1	0	0	1	0	0	1	5
hsa-miR-708	PTTG1IP	1	1	0	1	0	0	1	0	0	0	4
hsa-miR-708	PVR	1	1	0	1	0	0	1	0	0	1	5
hsa-miR-708	PXDN	1	1	0	1	0	0	1	0	0	1	5
hsa-miR-708	PXMP3	1	1	0	1	0	0	0	0	0	1	4
hsa-miR-708	PXMP4	1	1	0	1	0	0	1	0	0	1	5
hsa-miR-708	PYCRL	1	1	0	1	0	0	1	0	0	1	5
hsa-miR-708	QKI	1	1	0	1	0	0	1	0	0	1	5
hsa-miR-708	QSOX1	1	1	0	1	0	0	1	0	0	1	5
hsa-miR-708	QSOX2	1	1	0	1	0	0	1	0	0	0	4
hsa-miR-708	R3HDM2	1	1	0	1	0	0	1	0	0	1	5
hsa-miR-708	RAB11FIP4	1	1	0	0	0	0	1	0	0	1	4
hsa-miR-708	RAB11FIP5	1	1	0	1	0	0	1	0	0	1	5
hsa-miR-708	RAB14	1	1	0	1	0	0	1	0	0	1	5
hsa-miR-708	RAB15	1	1	0	1	0	0	1	0	0	1	5
hsa-miR-708	RAB18	1	1	0	1	0	0	1	0	0	1	5
hsa-miR-708	RAB1A	1	1	0	1	0	0	1	0	0	0	4
hsa-miR-708	RAB22A	1	1	0	1	0	0	1	0	0	1	5
hsa-miR-708	RAB23	1	1	0	1	0	0	1	0	0	1	5
hsa-miR-708	RAB24	1	1	0	1	0	0	1	0	0	1	5
hsa-miR-708	RAB28	1	1	0	1	0	0	1	0	0	1	5
hsa-miR-708	RAB2B	1	1	0	1	0	0	1	0	0	1	5
hsa-miR-708	RAB31	1	1	0	1	0	0	1	0	0	0	4
hsa-miR-708	RAB33B	1	1	0	1	0	0	1	0	0	1	5
hsa-miR-708	RAB36	1	1	0	1	0	0	1	0	0	1	5

hsa-miR-708	RAB6B	1	1	0	1	0	0	1	0	0	1	5
hsa-miR-708	RAB7A	1	1	0	1	0	0	1	0	0	0	4
hsa-miR-708	RAB8B	1	1	0	1	0	0	1	0	0	1	5
hsa-miR-708	RABIF	1	1	0	1	0	0	1	0	0	1	5
hsa-miR-708	RABL5	1	1	0	1	0	0	0	0	0	1	4
hsa-miR-708	RAD52	1	1	0	1	0	0	1	0	0	0	4
hsa-miR-708	RALBP1	1	1	0	1	0	0	1	0	0	1	5
hsa-miR-708	RALGPS1	1	1	0	1	0	0	1	0	0	0	4
hsa-miR-708	RAMP3	1	1	0	1	0	0	1	0	0	1	5
hsa-miR-708	RANBP10	1	1	0	1	0	0	1	0	0	1	5
hsa-miR-708	RAP1A	1	0	0	1	0	0	1	0	0	1	4
hsa-miR-708	RAP1GDS1	0	1	0	1	0	0	1	0	0	1	4
hsa-miR-708	RAPGEF1	1	1	0	0	0	0	1	0	0	1	4
hsa-miR-708	RAPGEF5	1	1	0	1	0	0	1	0	0	1	5
hsa-miR-708	RAPGEFL1	1	1	0	1	0	0	1	0	0	1	5
hsa-miR-708	RAPH1	1	1	0	0	0	0	1	0	0	1	4
hsa-miR-708	RARG	1	1	0	0	0	0	1	0	0	1	4
hsa-miR-708	RASA3	1	1	0	1	0	0	1	0	0	0	4
hsa-miR-708	RASD2	1	1	0	1	0	0	1	0	0	1	5
hsa-miR-708	RASGEF1A	1	1	0	1	0	0	1	0	0	1	5
hsa-miR-708	RASGRP1	1	1	0	1	0	0	1	0	0	0	4
hsa-miR-708	RASGRP4	0	1	0	1	0	0	1	0	0	1	4
hsa-miR-708	RASL12	1	1	0	1	0	0	1	0	0	0	4
hsa-miR-708	RASSF2	1	0	0	1	0	0	1	0	0	1	4
hsa-miR-708	RASSF4	1	1	0	1	0	0	1	0	0	1	5
hsa-miR-708	RAX	1	1	0	1	0	0	1	0	0	1	5
hsa-miR-708	RBBP5	1	1	0	1	0	0	1	0	0	1	5
hsa-miR-708	RBED1	1	1	0	1	0	0	1	0	0	1	5
hsa-miR-708	RBL1	1	1	0	0	0	0	1	0	0	1	4
hsa-miR-708	RBM13	1	1	0	1	0	0	1	0	0	0	4
hsa-miR-708	RBM23	1	1	0	1	0	0	1	0	0	1	5
hsa-miR-708	RBM33	1	1	0	1	0	0	0	0	0	1	4
hsa-miR-708	RBM35B	1	1	0	1	0	0	1	0	0	1	5
hsa-miR-708	RBM5	1	1	0	1	0	0	1	0	0	0	4
hsa-miR-708	RBMXL2	1	1	1	1	0	0	1	0	0	1	6
hsa-miR-708	RCAN1	1	1	0	1	0	0	1	0	0	1	5
hsa-miR-708	RCC2	1	0	0	1	0	0	1	0	0	1	4
hsa-miR-708	RCE1	1	1	0	1	0	0	1	0	0	1	5
hsa-miR-708	RCVRN	1	1	0	1	0	0	1	0	0	1	5
hsa-miR-708	RDX	1	1	0	0	0	0	1	0	0	1	4
hsa-miR-708	RELT	1	0	0	1	0	0	1	0	0	1	4
hsa-miR-708	REN	1	1	0	0	0	0	1	0	0	1	4
hsa-miR-708	REPS2	1	1	0	0	0	0	1	0	0	1	4
hsa-miR-708	RER1	1	1	0	1	0	0	1	0	0	1	5

hsa-miR-708	REV1	1	1	0	1	0	0	1	0	0	0	4
hsa-miR-708	REXO2	1	1	0	1	0	0	1	0	0	1	5
hsa-miR-708	REXO4	1	1	0	1	0	0	1	0	0	1	5
hsa-miR-708	RFX5	1	1	0	1	0	0	1	0	0	1	5
hsa-miR-708	RGL1	1	1	0	1	0	0	1	0	0	1	5
hsa-miR-708	RGMA	1	1	0	1	0	0	1	0	0	1	5
hsa-miR-708	RGS16	1	1	0	1	0	0	1	0	0	1	5
hsa-miR-708	RGS4	1	1	0	1	0	0	1	0	0	1	5
hsa-miR-708	RGS6	1	1	0	1	0	0	1	0	0	0	4
hsa-miR-708	RHBDD1	1	1	0	1	0	0	1	0	0	1	5
hsa-miR-708	RHBDD3	1	1	0	1	0	0	1	0	0	1	5
hsa-miR-708	RHBG	1	1	0	1	0	0	1	0	0	0	4
hsa-miR-708	RHO	1	1	1	1	0	0	1	0	0	1	6
hsa-miR-708	RHOF	1	1	0	1	0	0	1	0	0	1	5
hsa-miR-708	RHPN1	1	1	0	1	0	0	1	0	0	1	5
hsa-miR-708	RICH2	1	1	1	1	0	0	1	0	0	1	6
hsa-miR-708	RIMS3	1	1	0	1	0	0	1	0	0	1	5
hsa-miR-708	RIMS4	1	1	0	1	0	0	1	0	0	1	5
hsa-miR-708	RIN3	1	1	0	1	0	0	1	0	0	1	5
hsa-miR-708	RIPK1	1	1	0	0	0	0	1	0	0	1	4
hsa-miR-708	RNASE2	1	1	0	1	0	0	1	0	0	1	5
hsa-miR-708	RNASE3	1	1	0	1	0	0	1	0	0	1	5
hsa-miR-708	RNASEL	1	1	0	1	0	0	1	0	0	1	5
hsa-miR-708	RND2	1	1	0	1	0	0	1	0	0	1	5
hsa-miR-708	RNF138	1	1	0	1	0	0	1	0	0	1	5
hsa-miR-708	RNF141	1	1	0	1	0	0	1	0	0	1	5
hsa-miR-708	RNF144A	1	1	0	0	0	0	1	0	0	1	4
hsa-miR-708	RNF150	1	1	0	1	0	0	1	0	0	1	5
hsa-miR-708	RNF165	1	1	0	1	0	0	1	0	0	1	5
hsa-miR-708	RNF186	1	1	0	0	0	0	1	0	0	1	4
hsa-miR-708	RNF207	1	1	0	1	0	0	1	0	0	1	5
hsa-miR-708	RNF213	1	1	0	1	0	0	1	0	0	1	5
hsa-miR-708	RNF38	1	1	0	1	0	0	0	0	0	1	4
hsa-miR-708	RNF43	1	1	0	1	0	0	1	0	0	0	4
hsa-miR-708	RNGTT	1	1	0	1	0	0	1	0	0	0	4
hsa-miR-708	RNMTL1	1	1	0	1	0	0	1	0	0	1	5
hsa-miR-708	ROBO3	1	1	0	1	0	0	1	0	0	1	5
hsa-miR-708	ROD1	1	0	0	1	0	0	1	0	0	1	4
hsa-miR-708	RP13-347D8.3	0	1	0	1	0	0	1	0	0	1	4
hsa-miR-708	RP1L1	1	1	0	0	0	0	1	0	0	1	4
hsa-miR-708	RP3-402G11.5	1	1	0	0	0	0	1	0	0	1	4
hsa-miR-708	RP5-1022P6.2	1	1	0	1	0	0	1	0	0	0	4
hsa-miR-708	RPH3AL	1	1	0	1	0	0	1	0	0	1	5
hsa-miR-708	RPL15	1	1	0	1	0	0	1	0	0	1	5

hsa-miR-708	RPL22	1	1	0	1	0	0	1	0	0	0	4
hsa-miR-708	RPL28	1	1	0	1	0	0	1	0	0	1	5
hsa-miR-708	RPP25	1	1	0	1	0	0	1	0	0	1	5
hsa-miR-708	RPS6KA1	1	0	0	1	0	0	1	0	0	1	4
hsa-miR-708	RPS6KL1	1	1	0	1	0	0	1	0	0	1	5
hsa-miR-708	RPTN	1	1	1	1	0	0	0	0	0	0	4
hsa-miR-708	RREB1	1	1	0	0	0	0	1	0	0	1	4
hsa-miR-708	RRH	1	1	0	1	0	0	1	0	0	1	5
hsa-miR-708	RRM2B	1	1	0	1	0	0	1	0	0	1	5
hsa-miR-708	RS1	1	1	0	1	0	0	1	0	0	1	5
hsa-miR-708	RSAD1	1	1	0	1	0	0	1	0	0	0	4
hsa-miR-708	RSL1D1	1	1	0	1	0	0	1	0	0	1	5
hsa-miR-708	RSPO4	1	1	0	1	0	0	1	0	0	0	4
hsa-miR-708	RTKN2	1	1	0	1	0	0	1	0	0	1	5
hsa-miR-708	RTN4RL1	0	1	0	1	0	0	1	0	0	1	4
hsa-miR-708	RUFY2	1	1	0	1	0	0	1	0	0	0	4
hsa-miR-708	RUFY3	1	1	0	0	0	0	1	0	0	1	4
hsa-miR-708	RUNDC1	1	1	0	1	0	0	1	0	0	1	5
hsa-miR-708	S100A16	1	1	0	1	0	0	1	0	0	1	5
hsa-miR-708	S100A3	1	1	0	1	0	0	1	0	0	0	4
hsa-miR-708	SACM1L	1	1	1	1	0	0	1	0	0	1	6
hsa-miR-708	SAMD12	0	1	0	1	0	0	1	0	0	1	4
hsa-miR-708	SARM1	1	1	1	0	0	0	1	0	0	1	5
hsa-miR-708	SART1	1	1	0	1	0	0	1	0	0	0	4
hsa-miR-708	SC65	1	1	0	0	0	0	1	0	0	1	4
hsa-miR-708	SCARB2	1	1	1	1	0	0	1	0	0	1	6
hsa-miR-708	SCMH1	1	1	0	1	0	0	1	0	0	1	5
hsa-miR-708	SCML4	1	1	0	1	0	0	1	0	0	0	4
hsa-miR-708	SCN1B	1	0	0	1	0	0	1	0	0	1	4
hsa-miR-708	SCN4A	1	1	1	1	0	0	1	0	0	1	6
hsa-miR-708	SCN4B	1	1	0	1	0	0	1	0	0	1	5
hsa-miR-708	SCN5A	1	0	0	1	0	0	1	0	0	1	4
hsa-miR-708	SCRN2	1	1	0	1	0	0	1	0	0	0	4
hsa-miR-708	SCUBE1	1	1	0	1	0	0	1	0	0	1	5
hsa-miR-708	SDC1	1	0	0	1	0	0	1	0	0	1	4
hsa-miR-708	SDC3	1	1	1	1	0	0	0	0	0	0	4
hsa-miR-708	SDC4	1	1	0	0	0	0	1	0	0	1	4
hsa-miR-708	SDCCAG8	1	1	0	1	0	0	1	0	0	1	5
hsa-miR-708	SDF4	1	1	0	1	0	0	1	0	0	1	5
hsa-miR-708	SDHC	1	1	0	1	0	0	1	0	0	0	4
hsa-miR-708	SEC16A	0	1	0	1	0	0	1	0	0	1	4
hsa-miR-708	SECISBP2	1	1	0	1	0	0	1	0	0	1	5
hsa-miR-708	SELL	1	1	0	0	0	0	1	0	0	1	4
hsa-miR-708	SEMA4C	1	1	0	1	0	0	1	0	0	1	5

hsa-miR-708	SEMA4F	1	1	1	1	0	0	1	0	0	1	6
hsa-miR-708	SEMA4G	1	1	0	1	0	0	1	0	0	1	5
hsa-miR-708	SEMA5A	1	1	0	1	0	0	1	0	0	1	5
hsa-miR-708	SEMA6D	1	1	0	1	0	0	1	0	0	0	4
hsa-miR-708	SEMA7A	1	1	0	1	0	0	1	0	0	1	5
hsa-miR-708	SENP1	1	1	0	1	0	0	1	0	0	1	5
hsa-miR-708	SERBP1	1	1	0	1	0	0	1	0	0	0	4
hsa-miR-708	SERF2	1	1	0	1	0	0	1	0	0	0	4
hsa-miR-708	SERPINB8	1	1	0	1	0	0	0	0	0	1	4
hsa-miR-708	SERPINF2	1	1	0	1	0	0	1	0	0	1	5
hsa-miR-708	SESN1	1	1	1	1	0	0	1	0	0	1	6
hsa-miR-708	SETD3	0	1	0	1	0	0	1	0	0	1	4
hsa-miR-708	SETDB1	1	0	0	1	0	0	1	0	0	1	4
hsa-miR-708	SEZ6	1	1	0	1	0	0	1	0	0	0	4
hsa-miR-708	SF1	1	1	0	1	0	0	1	0	0	0	4
hsa-miR-708	SF3A1	1	1	0	0	0	0	1	0	0	1	4
hsa-miR-708	SF3B4	1	1	0	1	0	0	1	0	0	1	5
hsa-miR-708	SFRS11	1	1	1	1	0	0	1	0	0	1	6
hsa-miR-708	SFRS14	1	1	0	0	0	0	1	0	0	1	4
hsa-miR-708	SFRS6	1	1	1	1	0	0	1	0	0	1	6
hsa-miR-708	SFT2D3	1	1	0	0	0	0	1	0	0	1	4
hsa-miR-708	SFTPB	1	1	0	1	0	0	1	0	0	1	5
hsa-miR-708	SFXN1	1	1	0	0	0	0	1	0	0	1	4
hsa-miR-708	SFXN2	1	1	0	1	0	0	1	0	0	1	5
hsa-miR-708	SFXN5	1	1	0	1	0	0	1	0	0	1	5
hsa-miR-708	SGCA	1	1	0	1	0	0	1	0	0	1	5
hsa-miR-708	SGCD	1	0	0	1	0	0	1	0	0	1	4
hsa-miR-708	SGK269	1	1	1	1	0	0	1	0	0	1	6
hsa-miR-708	SGPL1	1	1	0	1	0	0	1	0	0	0	4
hsa-miR-708	SGSM1	1	1	0	1	0	0	1	0	0	1	5
hsa-miR-708	SGSM2	1	1	0	1	0	0	1	0	0	1	5
hsa-miR-708	SH2B1	1	1	0	1	0	0	1	0	0	1	5
hsa-miR-708	SH2B3	1	1	0	1	0	0	1	0	0	1	5
hsa-miR-708	SH3BP1	1	1	0	0	0	0	1	0	0	1	4
hsa-miR-708	SH3GL2	1	1	0	1	0	0	1	0	0	1	5
hsa-miR-708	SH3GLB1	1	1	0	1	0	0	1	0	0	1	5
hsa-miR-708	SH3PXD2B	1	1	0	1	0	0	1	0	0	1	5
hsa-miR-708	SH3TC2	1	1	1	0	0	0	1	0	0	1	5
hsa-miR-708	SHANK2	1	1	1	1	0	0	1	0	0	1	6
hsa-miR-708	SHANK3	0	1	0	1	0	0	1	0	0	1	4
hsa-miR-708	SHB	1	1	0	1	0	0	1	0	0	1	5
hsa-miR-708	SHF	1	1	0	1	0	0	1	0	0	0	4
hsa-miR-708	SHROOM2	1	1	0	1	0	0	1	0	0	1	5
hsa-miR-708	SIDT1	1	1	0	1	0	0	1	0	0	1	5

hsa-miR-708	SIDT2	1	1	0	1	0	0	1	0	0	1	5
hsa-miR-708	SIGLEC1	1	1	0	1	0	0	1	0	0	1	5
hsa-miR-708	SIGLEC11	1	1	0	1	0	0	1	0	0	1	5
hsa-miR-708	SIM1	1	1	0	1	0	0	1	0	0	0	4
hsa-miR-708	SIM2	1	1	0	1	0	0	1	0	0	1	5
hsa-miR-708	SIPA1L3	1	1	0	1	0	0	1	0	0	1	5
hsa-miR-708	SIRPG	1	1	0	1	0	0	1	0	0	0	4
hsa-miR-708	SIRT2	1	1	0	1	0	0	1	0	0	1	5
hsa-miR-708	SIRT3	1	1	1	1	0	0	1	0	0	1	6
hsa-miR-708	SKAP2	1	1	0	1	0	0	1	0	0	1	5
hsa-miR-708	SLAMF6	1	1	0	1	0	0	1	0	0	1	5
hsa-miR-708	SLBP	1	1	0	1	0	0	1	0	0	1	5
hsa-miR-708	SLC12A5	1	0	0	1	0	0	1	0	0	1	4
hsa-miR-708	SLC13A3	1	0	1	1	0	0	1	0	0	1	5
hsa-miR-708	SLC13A5	1	1	1	1	0	0	1	0	0	1	6
hsa-miR-708	SLC1A2	1	1	0	1	0	0	1	0	0	1	5
hsa-miR-708	SLC1A7	1	1	0	0	0	0	1	0	0	1	4
hsa-miR-708	SLC22A3	1	1	0	1	0	0	1	0	0	1	5
hsa-miR-708	SLC24A1	1	1	0	0	0	0	1	0	0	1	4
hsa-miR-708	SLC24A2	1	1	0	1	0	0	1	0	0	0	4
hsa-miR-708	SLC24A3	1	1	0	1	0	0	1	0	0	1	5
hsa-miR-708	SLC25A10	1	1	0	1	0	0	1	0	0	1	5
hsa-miR-708	SLC25A12	1	1	0	1	0	0	1	0	0	1	5
hsa-miR-708	SLC25A25	1	1	0	1	0	0	1	0	0	0	4
hsa-miR-708	SLC25A29	1	1	0	1	0	0	1	0	0	1	5
hsa-miR-708	SLC25A44	1	1	0	1	0	0	1	0	0	0	4
hsa-miR-708	SLC25A45	1	1	0	1	0	0	1	0	0	1	5
hsa-miR-708	SLC27A1	1	1	0	1	0	0	1	0	0	1	5
hsa-miR-708	SLC2A1	1	1	0	1	0	0	1	0	0	0	4
hsa-miR-708	SLC2A12	1	1	0	1	0	0	1	0	0	1	5
hsa-miR-708	SLC2A6	1	1	0	1	0	0	1	0	0	1	5
hsa-miR-708	SLC2A8	1	1	0	1	0	0	1	0	0	1	5
hsa-miR-708	SLC30A2	1	1	0	1	0	0	1	0	0	1	5
hsa-miR-708	SLC31A1	1	1	0	1	0	0	1	0	0	1	5
hsa-miR-708	SLC31A2	1	1	0	1	0	0	1	0	0	1	5
hsa-miR-708	SLC35C1	1	0	0	1	0	0	1	0	0	1	4
hsa-miR-708	SLC35E1	1	1	0	1	0	0	1	0	0	1	5
hsa-miR-708	SLC37A2	1	1	0	1	0	0	1	0	0	0	4
hsa-miR-708	SLC37A4	1	0	1	1	0	0	1	0	0	1	5
hsa-miR-708	SLC38A2	1	1	0	1	0	0	1	0	0	1	5
hsa-miR-708	SLC39A9	1	1	0	1	0	0	1	0	0	1	5
hsa-miR-708	SLC45A4	1	1	0	1	0	0	1	0	0	1	5
hsa-miR-708	SLC46A3	1	1	0	1	0	0	1	0	0	1	5
hsa-miR-708	SLC4A11	1	1	1	1	0	0	1	0	0	1	6

hsa-miR-708	SLC6A1	1	1	0	1	0	0	1	0	0	1	5
hsa-miR-708	SLC6A12	1	1	0	1	0	0	0	0	0	1	4
hsa-miR-708	SLC6A2	1	1	0	0	0	0	1	0	0	1	4
hsa-miR-708	SLC6A6	1	1	0	1	0	0	0	0	0	1	4
hsa-miR-708	SLC6A7	1	1	0	1	0	0	1	0	0	0	4
hsa-miR-708	SLC7A1	1	1	0	1	0	0	1	0	0	1	5
hsa-miR-708	SLC7A11	1	1	0	0	0	0	1	0	0	1	4
hsa-miR-708	SLC7A2	1	1	0	1	0	0	1	0	0	0	4
hsa-miR-708	SLC7A5	1	1	0	0	0	0	1	0	0	1	4
hsa-miR-708	SLC8A1	1	1	0	1	0	0	1	0	0	0	4
hsa-miR-708	SLC9A1	1	1	0	1	0	0	1	0	0	1	5
hsa-miR-708	SLC9A6	1	1	0	1	0	0	1	0	0	0	4
hsa-miR-708	SLCO1C1	1	1	0	1	0	0	1	0	0	1	5
hsa-miR-708	SLITRK1	1	1	0	1	0	0	1	0	0	1	5
hsa-miR-708	SMAD3	1	1	0	0	0	0	1	0	0	1	4
hsa-miR-708	SMAD4	1	1	0	1	0	0	1	0	0	1	5
hsa-miR-708	SMAP2	1	1	0	1	0	0	1	0	0	1	5
hsa-miR-708	SMARCC1	1	1	0	1	0	0	1	0	0	1	5
hsa-miR-708	SMARCC2	1	1	0	1	0	0	1	0	0	1	5
hsa-miR-708	SMARCD1	1	1	0	0	0	0	1	0	0	1	4
hsa-miR-708	SMC1A	1	1	0	1	0	0	1	0	0	1	5
hsa-miR-708	SMC1B	1	1	0	1	0	0	1	0	0	1	5
hsa-miR-708	SMG1	1	1	0	0	0	0	1	0	0	1	4
hsa-miR-708	SMG5	1	1	0	1	0	0	1	0	0	1	5
hsa-miR-708	SMG7	1	1	0	1	0	0	1	0	0	1	5
hsa-miR-708	SMURF2	1	1	1	1	0	0	1	0	0	1	6
hsa-miR-708	SMYD1	1	1	0	1	0	0	1	0	0	1	5
hsa-miR-708	SMYD5	1	1	0	1	0	0	1	0	0	1	5
hsa-miR-708	SNAP23	1	1	0	1	0	0	1	0	0	1	5
hsa-miR-708	SNAP25	1	1	0	1	0	0	1	0	0	1	5
hsa-miR-708	SNCAIP	1	1	0	1	0	0	1	0	0	1	5
hsa-miR-708	SNF1LK2	1	1	1	1	0	0	1	0	0	1	6
hsa-miR-708	SNIP	1	1	0	1	0	0	1	0	0	1	5
hsa-miR-708	SNPH	1	1	0	0	0	0	1	0	0	1	4
hsa-miR-708	SNTB2	1	1	0	1	0	0	1	0	0	1	5
hsa-miR-708	SNUPN	1	1	0	1	0	0	0	0	0	1	4
hsa-miR-708	SNX1	1	1	0	1	0	0	1	0	0	1	5
hsa-miR-708	SNX11	1	1	0	1	0	0	0	0	0	1	4
hsa-miR-708	SNX19	1	1	0	1	0	0	1	0	0	1	5
hsa-miR-708	SNX22	1	1	0	1	0	0	1	0	0	1	5
hsa-miR-708	SNX27	1	1	0	1	0	0	1	0	0	1	5
hsa-miR-708	SNX29	1	1	0	1	0	0	1	0	0	1	5
hsa-miR-708	SNX33	1	1	0	1	0	0	1	0	0	1	5
hsa-miR-708	SOBP	1	1	0	1	0	0	1	0	0	0	4

hsa-miR-708	SOCS7	1	1	0	1	0	0	1	0	0	1	5
hsa-miR-708	SOD3	1	1	0	1	0	0	1	0	0	1	5
hsa-miR-708	SORCS1	1	1	0	1	0	0	1	0	0	1	5
hsa-miR-708	SORCS2	1	1	0	1	0	0	1	0	0	1	5
hsa-miR-708	SORT1	1	1	0	0	0	0	1	0	0	1	4
hsa-miR-708	SOX10	1	1	0	1	0	0	1	0	0	1	5
hsa-miR-708	SOX3	1	1	0	1	0	0	1	0	0	0	4
hsa-miR-708	SP1	1	1	0	0	0	0	1	0	0	1	4
hsa-miR-708	SP100	0	1	0	1	0	0	1	0	0	1	4
hsa-miR-708	SPAG1	1	0	0	1	0	0	1	0	0	1	4
hsa-miR-708	SPARC	0	1	1	1	0	0	1	0	0	1	5
hsa-miR-708	SPATA8	1	1	0	1	0	0	1	0	0	1	5
hsa-miR-708	SPHK1	1	1	0	1	0	0	1	0	0	1	5
hsa-miR-708	SPINT1	1	0	0	1	0	0	1	0	0	1	4
hsa-miR-708	SPOCK2	1	1	0	1	0	0	1	0	0	1	5
hsa-miR-708	SPRR1A	1	1	0	1	0	0	1	0	0	0	4
hsa-miR-708	SPRYD3	0	1	0	1	0	0	1	0	0	1	4
hsa-miR-708	SPTA1	0	1	0	1	0	0	1	0	0	1	4
hsa-miR-708	SPTB	1	1	0	1	0	0	1	0	0	1	5
hsa-miR-708	SPTBN5	1	1	0	0	0	0	1	0	0	1	4
hsa-miR-708	SPTLC2	1	1	0	1	0	0	1	0	0	1	5
hsa-miR-708	SQSTM1	1	1	0	1	0	0	1	0	0	0	4
hsa-miR-708	SRCRB4D	1	1	0	0	0	0	1	0	0	1	4
hsa-miR-708	SRD5A1	1	1	0	1	0	0	1	0	0	1	5
hsa-miR-708	SRF	1	1	0	1	0	0	1	0	0	1	5
hsa-miR-708	SRGAP2	1	1	0	0	0	0	1	0	0	1	4
hsa-miR-708	SRGAP3	1	1	0	1	0	0	1	0	0	1	5
hsa-miR-708	SRL	1	1	0	0	0	0	1	0	0	1	4
hsa-miR-708	SRP72	1	1	0	1	0	0	1	0	0	1	5
hsa-miR-708	SRPR	1	1	0	1	0	0	1	0	0	1	5
hsa-miR-708	SRPRB	1	1	1	1	0	0	1	0	0	1	6
hsa-miR-708	SRPX2	1	1	0	1	0	0	1	0	0	1	5
hsa-miR-708	SRRD	1	1	1	1	0	0	1	0	0	1	6
hsa-miR-708	SRY	1	1	0	1	0	0	1	0	0	0	4
hsa-miR-708	SS18L2	1	1	0	1	0	0	1	0	0	0	4
hsa-miR-708	SSH2	1	1	0	1	0	0	1	0	0	1	5
hsa-miR-708	SSRP1	1	1	0	1	0	0	1	0	0	1	5
hsa-miR-708	ST7	1	1	0	1	0	0	1	0	0	1	5
hsa-miR-708	ST7L	1	1	0	1	0	0	1	0	0	1	5
hsa-miR-708	ST8SIA2	1	1	0	1	0	0	1	0	0	1	5
hsa-miR-708	ST8SIA3	1	1	0	1	0	0	1	0	0	1	5
hsa-miR-708	STAC2	1	1	0	1	0	0	1	0	0	0	4
hsa-miR-708	STAM2	1	1	0	1	0	0	1	0	0	1	5
hsa-miR-708	STARD8	1	0	0	1	0	0	1	0	0	1	4

hsa-miR-708	STC1	1	1	0	1	0	0	1	0	0	0	4
hsa-miR-708	STC2	1	1	0	1	0	0	1	0	0	1	5
hsa-miR-708	STEAP4	1	1	0	1	0	0	1	0	0	0	4
hsa-miR-708	STK24	1	1	0	1	0	0	1	0	0	1	5
hsa-miR-708	STK32A	1	1	0	1	0	0	0	0	0	1	4
hsa-miR-708	STK35	1	1	0	1	0	0	1	0	0	1	5
hsa-miR-708	STK36	1	1	0	1	0	0	1	0	0	1	5
hsa-miR-708	STK4	1	1	0	1	0	0	1	0	0	1	5
hsa-miR-708	STOM	1	1	0	1	0	0	1	0	0	1	5
hsa-miR-708	STOML3	1	1	0	1	0	0	1	0	0	1	5
hsa-miR-708	STRN4	1	1	0	1	0	0	1	0	0	1	5
hsa-miR-708	STS	1	1	0	1	0	0	1	0	0	1	5
hsa-miR-708	STX16	1	1	1	0	0	0	1	0	0	1	5
hsa-miR-708	STX6	1	1	0	1	0	0	1	0	0	1	5
hsa-miR-708	STXBP6	1	1	0	1	0	0	1	0	0	0	4
hsa-miR-708	SUFU	1	1	0	1	0	0	1	0	0	1	5
hsa-miR-708	SUPT16H	1	1	0	1	0	0	1	0	0	0	4
hsa-miR-708	SUPT4H1	1	1	0	1	0	0	1	0	0	1	5
hsa-miR-708	SUSD1	1	1	0	1	0	0	1	0	0	1	5
hsa-miR-708	SUV39H1	1	1	0	1	0	0	1	0	0	1	5
hsa-miR-708	SV2A	1	1	0	1	0	0	1	0	0	1	5
hsa-miR-708	SYCE2	1	1	0	1	0	0	1	0	0	1	5
hsa-miR-708	SYDE1	1	1	0	1	0	0	1	0	0	1	5
hsa-miR-708	SYN2	1	1	0	1	0	0	1	0	0	1	5
hsa-miR-708	SYNGR1	1	1	0	1	0	0	1	0	0	1	5
hsa-miR-708	SYNJ2	1	1	0	1	0	0	1	0	0	1	5
hsa-miR-708	SYNJ2BP	0	1	0	1	0	0	1	0	0	1	4
hsa-miR-708	SYNPO2	1	1	0	1	0	0	0	0	0	1	4
hsa-miR-708	SYPL2	1	1	0	1	0	0	1	0	0	1	5
hsa-miR-708	SYT11	1	1	0	1	0	0	1	0	0	1	5
hsa-miR-708	SYT13	1	1	0	1	0	0	1	0	0	0	4
hsa-miR-708	SYT7	1	1	0	1	0	0	1	0	0	1	5
hsa-miR-708	SYT9	1	1	0	1	0	0	1	0	0	1	5
hsa-miR-708	TAF4	1	1	0	1	0	0	1	0	0	0	4
hsa-miR-708	TAGAP	1	1	0	1	0	0	1	0	0	1	5
hsa-miR-708	TAGLN	1	0	0	1	0	0	1	0	0	1	4
hsa-miR-708	TAGLN2	1	1	0	1	0	0	1	0	0	1	5
hsa-miR-708	TAGLN3	1	1	0	1	0	0	0	0	0	1	4
hsa-miR-708	TAL1	1	1	0	1	0	0	1	0	0	0	4
hsa-miR-708	TANC1	1	1	0	1	0	0	1	0	0	1	5
hsa-miR-708	TAP1	1	1	0	1	0	0	0	0	0	1	4
hsa-miR-708	TAZ	1	0	0	1	0	0	1	0	0	1	4
hsa-miR-708	TBC1D13	1	1	0	1	0	0	1	0	0	1	5
hsa-miR-708	TBC1D16	1	1	0	1	0	0	1	0	0	1	5

hsa-miR-708	TBC1D24	1	1	0	0	0	0	1	0	0	1	4
hsa-miR-708	TBC1D26	0	1	0	1	0	0	1	0	0	1	4
hsa-miR-708	TBC1D2B	1	1	0	1	0	0	0	0	0	1	4
hsa-miR-708	TBC1D4	1	1	0	1	0	0	1	0	0	1	5
hsa-miR-708	TBC1D5	1	0	0	1	0	0	1	0	0	1	4
hsa-miR-708	TBCB	1	1	0	1	0	0	1	0	0	1	5
hsa-miR-708	TBKBP1	1	1	0	1	0	0	1	0	0	1	5
hsa-miR-708	TC2N	1	1	0	1	0	0	1	0	0	1	5
hsa-miR-708	TCF7	1	1	0	1	0	0	1	0	0	1	5
hsa-miR-708	TCTA	1	1	0	0	0	0	1	0	0	1	4
hsa-miR-708	TEAD3	1	1	0	1	0	0	1	0	0	1	5
hsa-miR-708	TEF	1	1	0	1	0	0	1	0	0	1	5
hsa-miR-708	TEGT	1	1	0	1	0	0	1	0	0	1	5
hsa-miR-708	TERF2IP	1	1	0	0	0	0	1	0	0	1	4
hsa-miR-708	TEX14	1	1	0	1	0	0	1	0	0	1	5
hsa-miR-708	TEX261	1	1	0	1	0	0	1	0	0	1	5
hsa-miR-708	TFAP2B	1	1	0	1	0	0	1	0	0	1	5
hsa-miR-708	TFCP2	1	1	0	1	0	0	1	0	0	0	4
hsa-miR-708	TFCP2L1	1	1	0	1	0	0	1	0	0	0	4
hsa-miR-708	TFEB	1	1	0	1	0	0	1	0	0	1	5
hsa-miR-708	TFR2	1	1	0	1	0	0	1	0	0	1	5
hsa-miR-708	TFRC	1	1	0	1	0	0	0	0	0	1	4
hsa-miR-708	TGM2	1	1	0	1	0	0	1	0	0	1	5
hsa-miR-708	TGM4	1	1	0	1	0	0	1	0	0	0	4
hsa-miR-708	TGM7	1	1	0	1	0	0	1	0	0	0	4
hsa-miR-708	TGOLN2	1	1	0	1	0	0	1	0	0	1	5
hsa-miR-708	THG1L	1	1	0	1	0	0	1	0	0	0	4
hsa-miR-708	THRB	1	1	0	1	0	0	0	0	0	1	4
hsa-miR-708	THSD4	1	1	0	1	0	0	1	0	0	1	5
hsa-miR-708	TIE1	1	1	0	1	0	0	1	0	0	1	5
hsa-miR-708	TIMELESS	1	1	0	1	0	0	1	0	0	1	5
hsa-miR-708	TIMM17B	1	1	0	1	0	0	1	0	0	1	5
hsa-miR-708	TLK1	1	0	0	1	0	0	1	0	0	1	4
hsa-miR-708	TLN2	1	1	0	1	0	0	1	0	0	1	5
hsa-miR-708	TLR4	1	1	0	1	0	0	1	0	0	0	4
hsa-miR-708	TLR6	1	1	0	1	0	0	1	0	0	1	5
hsa-miR-708	TLR7	1	1	0	1	0	0	1	0	0	1	5
hsa-miR-708	TM4SF5	1	1	0	1	0	0	1	0	0	0	4
hsa-miR-708	TMCC1	1	1	0	1	0	0	1	0	0	0	4
hsa-miR-708	TMCC2	1	1	0	1	0	0	1	0	0	1	5
hsa-miR-708	TMCO3	1	1	0	1	0	0	1	0	0	0	4
hsa-miR-708	TMCO6	1	1	0	1	0	0	1	0	0	1	5
hsa-miR-708	TMCO7	1	1	0	1	0	0	1	0	0	0	4
hsa-miR-708	TMEM1	1	1	0	1	0	0	1	0	0	0	4

hsa-miR-708	TMEM104	0	1	0	1	0	0	1	0	0	1	4
hsa-miR-708	TMEM106B	1	1	0	1	0	0	0	0	0	1	4
hsa-miR-708	TMEM109	1	1	0	1	0	0	1	0	0	1	5
hsa-miR-708	TMEM115	1	1	0	1	0	0	1	0	0	1	5
hsa-miR-708	TMEM121	1	1	0	1	0	0	1	0	0	0	4
hsa-miR-708	TMEM127	1	1	1	1	0	0	1	0	0	1	6
hsa-miR-708	TMEM132B	1	1	0	1	0	0	1	0	0	1	5
hsa-miR-708	TMEM156	1	1	0	1	0	0	1	0	0	1	5
hsa-miR-708	TMEM16G	0	1	0	1	0	0	1	0	0	1	4
hsa-miR-708	TMEM16K	1	1	0	1	0	0	1	0	0	0	4
hsa-miR-708	TMEM170	1	1	1	1	0	0	1	0	0	1	6
hsa-miR-708	TMEM18	1	1	0	1	0	0	1	0	0	1	5
hsa-miR-708	TMEM180	1	1	0	1	0	0	1	0	0	1	5
hsa-miR-708	TMEM184A	1	1	0	1	0	0	1	0	0	1	5
hsa-miR-708	TMEM184B	1	1	0	1	0	0	1	0	0	1	5
hsa-miR-708	TMEM188	0	1	0	1	0	0	1	0	0	1	4
hsa-miR-708	TMEM194	1	1	0	1	0	0	0	0	0	1	4
hsa-miR-708	TMEM200B	0	1	0	1	0	0	1	0	0	1	4
hsa-miR-708	TMEM201	1	1	0	1	0	0	0	0	0	1	4
hsa-miR-708	TMEM28	1	1	0	1	0	0	1	0	0	0	4
hsa-miR-708	TMEM32	1	1	0	1	0	0	1	0	0	1	5
hsa-miR-708	TMEM43	1	1	0	1	0	0	1	0	0	1	5
hsa-miR-708	TMEM46	1	1	0	1	0	0	1	0	0	1	5
hsa-miR-708	TMEM59	1	1	0	1	0	0	1	0	0	0	4
hsa-miR-708	TMEM79	1	1	0	0	0	0	1	0	0	1	4
hsa-miR-708	TMEM92	1	1	0	1	0	0	1	0	0	1	5
hsa-miR-708	TMIE	1	1	0	1	0	0	1	0	0	0	4
hsa-miR-708	TMPRSS11D	1	1	0	1	0	0	1	0	0	1	5
hsa-miR-708	TMPRSS3	1	1	0	1	0	0	0	0	0	1	4
hsa-miR-708	TMPRSS4	0	1	0	1	0	0	1	0	0	1	4
hsa-miR-708	TMTC1	1	1	0	1	0	0	1	0	0	1	5
hsa-miR-708	TMUB1	1	0	0	1	0	0	1	0	0	1	4
hsa-miR-708	TNFRSF10D	1	1	0	1	0	0	1	0	0	1	5
hsa-miR-708	TNFRSF12A	1	1	0	1	0	0	1	0	0	1	5
hsa-miR-708	TNFRSF13C	1	1	0	1	0	0	1	0	0	1	5
hsa-miR-708	TNFRSF19	1	1	0	1	0	0	1	0	0	0	4
hsa-miR-708	TNK1	1	1	0	1	0	0	1	0	0	1	5
hsa-miR-708	TNNI1	1	1	0	1	0	0	1	0	0	1	5
hsa-miR-708	TNNT2	1	1	0	1	0	0	1	0	0	1	5
hsa-miR-708	TNPO1	1	0	0	1	0	0	1	0	0	1	4
hsa-miR-708	TNRC6A	1	1	0	1	0	0	1	0	0	0	4
hsa-miR-708	TNS1	1	1	0	1	0	0	1	0	0	1	5
hsa-miR-708	TNS3	1	1	0	1	0	0	1	0	0	1	5
hsa-miR-708	TNS4	1	1	1	1	0	0	1	0	0	1	6

hsa-miR-708	TOM1L2	0	1	0	1	0	0	1	0	0	1	4
hsa-miR-708	TOMM34	1	1	0	1	0	0	1	0	0	1	5
hsa-miR-708	TOR1A	1	1	0	1	0	0	1	0	0	1	5
hsa-miR-708	TOR1B	0	1	0	1	0	0	1	0	0	1	4
hsa-miR-708	TP53INP1	1	1	0	1	0	0	1	0	0	0	4
hsa-miR-708	TP63	1	1	0	1	0	0	1	0	0	1	5
hsa-miR-708	TPI1	1	1	0	1	0	0	1	0	0	1	5
hsa-miR-708	TPMT	1	1	1	1	0	0	1	0	0	1	6
hsa-miR-708	TPPP	1	1	0	1	0	0	1	0	0	0	4
hsa-miR-708	TPRG1L	1	1	0	1	0	0	1	0	0	1	5
hsa-miR-708	TRAF1	1	1	0	1	0	0	1	0	0	1	5
hsa-miR-708	TRAF7	1	1	0	1	0	0	1	0	0	1	5
hsa-miR-708	TRAM2	1	1	0	1	0	0	1	0	0	1	5
hsa-miR-708	TRAT1	1	1	0	1	0	0	1	0	0	1	5
hsa-miR-708	TREH	0	1	0	1	0	0	1	0	0	1	4
hsa-miR-708	TREML4	1	1	0	1	0	0	1	0	0	1	5
hsa-miR-708	TRH	1	1	0	1	0	0	1	0	0	0	4
hsa-miR-708	TRIB2	1	1	0	1	0	0	1	0	0	1	5
hsa-miR-708	TRIM14	1	1	0	1	0	0	1	0	0	1	5
hsa-miR-708	TRIM23	1	1	1	1	0	0	1	0	0	1	6
hsa-miR-708	TRIM25	1	1	0	1	0	0	1	0	0	1	5
hsa-miR-708	TRIM33	1	1	0	1	0	0	1	0	0	0	4
hsa-miR-708	TRIM48	1	1	0	1	0	0	1	0	0	0	4
hsa-miR-708	TRIM65	1	1	0	1	0	0	1	0	0	1	5
hsa-miR-708	TRIM67	0	1	0	1	0	0	1	0	0	1	4
hsa-miR-708	TRIT1	1	1	0	1	0	0	1	0	0	1	5
hsa-miR-708	TRPC4AP	1	1	0	1	0	0	1	0	0	1	5
hsa-miR-708	TRPS1	1	1	0	1	0	0	1	0	0	1	5
hsa-miR-708	TRUB1	1	1	0	0	0	0	1	0	0	1	4
hsa-miR-708	TSC22D1	1	1	0	1	0	0	1	0	0	1	5
hsa-miR-708	TSPAN14	0	1	0	1	0	0	1	0	0	1	4
hsa-miR-708	TSPAN17	1	1	0	1	0	0	1	0	0	1	5
hsa-miR-708	TSPAN18	1	1	0	1	0	0	1	0	0	1	5
hsa-miR-708	TSPAN6	1	1	0	1	0	0	1	0	0	0	4
hsa-miR-708	TSPAN7	1	1	0	1	0	0	1	0	0	1	5
hsa-miR-708	TSPYL4	1	1	0	1	0	0	1	0	0	1	5
hsa-miR-708	TSSC4	0	1	0	1	0	0	1	0	0	1	4
hsa-miR-708	TTBK1	1	1	0	1	0	0	1	0	0	1	5
hsa-miR-708	TTC23	1	0	0	1	0	0	1	0	0	1	4
hsa-miR-708	TTC31	1	1	0	0	0	0	1	0	0	1	4
hsa-miR-708	TTC9	1	1	0	1	0	0	1	0	0	1	5
hsa-miR-708	TTL	1	1	0	0	0	0	1	0	0	1	4
hsa-miR-708	TTLL12	1	1	0	1	0	0	1	0	0	1	5
hsa-miR-708	TTLL3	1	1	0	1	0	0	0	0	0	1	4

hsa-miR-708	TUB	1	1	0	1	0	0	1	0	0	1	5
hsa-miR-708	TUBB	1	1	0	1	0	0	0	0	0	1	4
hsa-miR-708	TUBB4	1	1	0	1	0	0	1	0	0	1	5
hsa-miR-708	TUSC5	1	1	0	0	0	0	1	0	0	1	4
hsa-miR-708	TXLNA	1	1	0	1	0	0	1	0	0	0	4
hsa-miR-708	TXNDC4	1	1	0	1	0	0	1	0	0	1	5
hsa-miR-708	TXNL1	1	1	0	1	0	0	1	0	0	1	5
hsa-miR-708	TXNL4B	1	0	0	1	0	0	1	0	0	1	4
hsa-miR-708	TXNRD2	1	1	0	1	0	0	1	0	0	1	5
hsa-miR-708	TYRO3	1	1	0	0	0	0	1	0	0	1	4
hsa-miR-708	UBA6	1	1	0	1	0	0	1	0	0	1	5
hsa-miR-708	UBAP1	1	1	0	1	0	0	1	0	0	0	4
hsa-miR-708	UBE2A	1	1	0	1	0	0	0	0	0	1	4
hsa-miR-708	UBE2G1	1	1	0	1	0	0	1	0	0	1	5
hsa-miR-708	UBE2G2	1	1	0	1	0	0	0	0	0	1	4
hsa-miR-708	UBE2J2	1	1	0	1	0	0	0	0	0	1	4
hsa-miR-708	UBE2N	1	1	0	1	0	0	1	0	0	1	5
hsa-miR-708	UBE2NL	1	1	0	1	0	0	1	0	0	0	4
hsa-miR-708	UBE2R2	1	1	1	1	0	0	1	0	0	1	6
hsa-miR-708	UBE2Z	1	1	0	1	0	0	1	0	0	1	5
hsa-miR-708	UBE3C	1	1	0	0	0	0	1	0	0	1	4
hsa-miR-708	UBFD1	1	1	0	1	0	0	1	0	0	1	5
hsa-miR-708	UBL4A	1	1	0	1	0	0	1	0	0	1	5
hsa-miR-708	UBN1	1	0	0	1	0	0	1	0	0	1	4
hsa-miR-708	UBQLNL	1	1	0	1	0	0	1	0	0	1	5
hsa-miR-708	UBTD1	1	1	0	1	0	0	1	0	0	0	4
hsa-miR-708	UBTD2	1	1	0	1	0	0	1	0	0	1	5
hsa-miR-708	UBTF	1	1	0	1	0	0	0	0	0	1	4
hsa-miR-708	UBXD7	1	1	0	1	0	0	1	0	0	0	4
hsa-miR-708	UBXD8	1	1	0	1	0	0	1	0	0	1	5
hsa-miR-708	UCP2	1	1	0	1	0	0	1	0	0	0	4
hsa-miR-708	ULBP1	1	1	0	1	0	0	1	0	0	1	5
hsa-miR-708	ULK1	1	1	0	1	0	0	1	0	0	1	5
hsa-miR-708	ULK3	1	1	0	1	0	0	1	0	0	1	5
hsa-miR-708	UNC119	1	1	0	1	0	0	1	0	0	0	4
hsa-miR-708	UNC119B	1	1	0	1	0	0	1	0	0	1	5
hsa-miR-708	UNC45B	1	1	0	1	0	0	1	0	0	1	5
hsa-miR-708	UNC5A	1	1	0	0	0	0	1	0	0	1	4
hsa-miR-708	UNK	1	1	1	1	0	0	1	0	0	1	6
hsa-miR-708	UPB1	1	1	0	1	0	0	1	0	0	1	5
hsa-miR-708	UPF1	1	1	0	1	0	0	1	0	0	0	4
hsa-miR-708	UPK3B	0	1	0	1	0	0	1	0	0	1	4
hsa-miR-708	UQCR	1	1	0	1	0	0	1	0	0	1	5
hsa-miR-708	URG4	1	1	0	1	0	0	0	0	0	1	4

hsa-miR-708	UROD	1	1	0	1	0	0	1	0	0	0	4
hsa-miR-708	USH1G	1	1	0	1	0	0	1	0	0	1	5
hsa-miR-708	USH2A	1	1	0	0	0	0	1	0	0	1	4
hsa-miR-708	USP22	1	1	0	1	0	0	1	0	0	1	5
hsa-miR-708	USP31	0	1	0	1	0	0	1	0	0	1	4
hsa-miR-708	USP39	1	1	0	1	0	0	1	0	0	0	4
hsa-miR-708	USP9X	1	1	1	1	0	0	1	0	0	1	6
hsa-miR-708	USP9Y	1	1	1	1	0	0	1	0	0	1	6
hsa-miR-708	UVRAG	1	1	0	0	0	0	1	0	0	1	4
hsa-miR-708	VAC14	1	1	0	1	0	0	1	0	0	1	5
hsa-miR-708	VAMP2	0	1	0	1	0	0	1	0	0	1	4
hsa-miR-708	VANGL1	1	1	0	1	0	0	1	0	0	1	5
hsa-miR-708	VANGL2	1	1	0	1	0	0	1	0	0	1	5
hsa-miR-708	VAPB	1	1	0	1	0	0	1	0	0	1	5
hsa-miR-708	VASH1	1	1	0	1	0	0	1	0	0	1	5
hsa-miR-708	VASH2	1	1	0	1	0	0	1	0	0	0	4
hsa-miR-708	VASP	1	1	0	1	0	0	1	0	0	0	4
hsa-miR-708	VAV2	1	1	0	1	0	0	0	0	0	1	4
hsa-miR-708	VEZF1	1	1	0	1	0	0	1	0	0	1	5
hsa-miR-708	VEZT	0	1	0	1	0	0	1	0	0	1	4
hsa-miR-708	VGLL3	1	1	0	0	0	0	1	0	0	1	4
hsa-miR-708	VGLL4	1	1	0	1	0	0	0	0	0	1	4
hsa-miR-708	VIPR1	1	1	0	1	0	0	1	0	0	1	5
hsa-miR-708	VIPR2	1	1	0	1	0	0	1	0	0	0	4
hsa-miR-708	VPS13A	1	1	0	1	0	0	1	0	0	1	5
hsa-miR-708	VPS13D	1	1	0	1	0	0	1	0	0	1	5
hsa-miR-708	VPS16	1	1	0	1	0	0	1	0	0	1	5
hsa-miR-708	VPS37B	1	1	0	1	0	0	1	0	0	1	5
hsa-miR-708	VPS39	1	1	0	1	0	0	1	0	0	1	5
hsa-miR-708	VPS4A	1	1	0	1	0	0	1	0	0	1	5
hsa-miR-708	VWA3A	1	1	0	1	0	0	1	0	0	1	5
hsa-miR-708	WASF3	1	1	0	1	0	0	1	0	0	1	5
hsa-miR-708	WDR26	1	1	0	1	0	0	1	0	0	0	4
hsa-miR-708	WDR27	1	1	0	1	0	0	1	0	0	1	5
hsa-miR-708	WDR32	1	1	0	1	0	0	1	0	0	1	5
hsa-miR-708	WDR35	1	1	0	1	0	0	1	0	0	0	4
hsa-miR-708	WDR37	1	1	0	1	0	0	1	0	0	1	5
hsa-miR-708	WDR40A	1	1	0	1	0	0	1	0	0	0	4
hsa-miR-708	WDR5	1	1	0	0	0	0	1	0	0	1	4
hsa-miR-708	WDR68	1	1	0	1	0	0	1	0	0	1	5
hsa-miR-708	WDR76	1	1	0	1	0	0	1	0	0	1	5
hsa-miR-708	WHSC1	1	1	0	1	0	0	0	0	0	1	4
hsa-miR-708	WIPF1	1	0	0	1	0	0	1	0	0	1	4
hsa-miR-708	WIPF2	1	1	1	1	0	0	1	0	0	1	6

hsa-miR-708	WIPI2	1	0	0	1	0	0	1	0	0	1	4
hsa-miR-708	WNT10B	1	1	0	1	0	0	1	0	0	0	4
hsa-miR-708	WNT2B	1	1	0	1	0	0	1	0	0	0	4
hsa-miR-708	WNT4	0	1	0	1	0	0	1	0	0	1	4
hsa-miR-708	WNT5B	1	1	0	1	0	0	1	0	0	1	5
hsa-miR-708	WNT7B	1	1	0	1	0	0	1	0	0	0	4
hsa-miR-708	WNT8B	1	1	0	1	0	0	1	0	0	1	5
hsa-miR-708	WSB2	1	1	1	1	0	0	1	0	0	1	6
hsa-miR-708	WSCD1	1	1	0	1	0	0	1	0	0	1	5
hsa-miR-708	XAF1	1	1	0	1	0	0	0	0	0	1	4
hsa-miR-708	XDH	1	1	0	1	0	0	1	0	0	1	5
hsa-miR-708	XIRP1	1	1	0	1	0	0	1	0	0	0	4
hsa-miR-708	XK	1	1	0	1	0	0	1	0	0	1	5
hsa-miR-708	XPR1	0	1	0	1	0	0	1	0	0	1	4
hsa-miR-708	XYLT1	1	1	0	0	0	0	1	0	0	1	4
hsa-miR-708	YEATS2	1	1	0	1	0	0	1	0	0	0	4
hsa-miR-708	YES1	1	1	0	1	0	0	1	0	0	1	5
hsa-miR-708	YIPF2	1	1	0	1	0	0	1	0	0	0	4
hsa-miR-708	YIPF3	1	1	0	1	0	0	1	0	0	1	5
hsa-miR-708	YPEL1	1	1	0	1	0	0	1	0	0	1	5
hsa-miR-708	YPEL2	1	1	0	1	0	0	1	0	0	1	5
hsa-miR-708	YPEL3	1	0	1	1	0	0	1	0	0	1	5
hsa-miR-708	YWHAH	1	1	0	1	0	0	1	0	0	1	5
hsa-miR-708	YWHAZ	1	0	0	1	0	0	1	0	0	1	4
hsa-miR-708	ZADH2	1	1	0	1	0	0	1	0	0	1	5
hsa-miR-708	ZAK	1	0	0	1	0	0	1	0	0	1	4
hsa-miR-708	ZBED4	1	1	0	1	0	0	1	0	0	1	5
hsa-miR-708	ZBTB46	1	1	0	1	0	0	1	0	0	1	5
hsa-miR-708	ZBTB47	1	1	0	1	0	0	1	0	0	1	5
hsa-miR-708	ZBTB5	1	1	0	1	0	0	1	0	0	1	5
hsa-miR-708	ZBTB6	1	1	0	1	0	0	1	0	0	1	5
hsa-miR-708	ZC3H12B	1	1	0	1	0	0	1	0	0	1	5
hsa-miR-708	ZC3H6	1	1	0	1	0	0	1	0	0	1	5
hsa-miR-708	ZC3HAV1L	1	1	0	1	0	0	1	0	0	1	5
hsa-miR-708	ZDHHC18	1	1	0	1	0	0	1	0	0	1	5
hsa-miR-708	ZFAT	1	1	0	1	0	0	1	0	0	1	5
hsa-miR-708	ZFHX3	1	1	0	0	0	0	1	0	0	1	4
hsa-miR-708	ZFP106	1	1	0	1	0	0	1	0	0	1	5
hsa-miR-708	ZFP14	1	1	0	1	0	0	1	0	0	1	5
hsa-miR-708	ZFP41	1	1	0	1	0	0	1	0	0	0	4
hsa-miR-708	ZFP91	1	1	0	1	0	0	1	0	0	1	5
hsa-miR-708	ZFYVE1	1	1	0	0	0	0	1	0	0	1	4
hsa-miR-708	ZFYVE21	1	1	0	1	0	0	1	0	0	0	4
hsa-miR-708	ZFYVE26	1	1	0	1	0	0	1	0	0	1	5

hsa-miR-708	ZHX3	1	1	0	1	0	0	1	0	0	1	5
hsa-miR-708	ZIC4	1	1	0	0	0	0	1	0	0	1	4
hsa-miR-708	ZIK1	1	1	1	1	0	0	1	0	0	1	6
hsa-miR-708	ZMIZ2	1	1	0	1	0	0	1	0	0	1	5
hsa-miR-708	ZMYND8	1	1	0	1	0	0	1	0	0	0	4
hsa-miR-708	ZNF10	1	1	0	1	0	0	1	0	0	1	5
hsa-miR-708	ZNF14	0	1	0	1	0	0	1	0	0	1	4
hsa-miR-708	ZNF19	1	1	0	1	0	0	1	0	0	1	5
hsa-miR-708	ZNF192	1	1	1	1	0	0	1	0	0	1	6
hsa-miR-708	ZNF213	1	1	0	1	0	0	1	0	0	0	4
hsa-miR-708	ZNF217	1	1	0	1	0	0	1	0	0	1	5
hsa-miR-708	ZNF22	1	1	0	1	0	0	1	0	0	1	5
hsa-miR-708	ZNF25	1	1	0	1	0	0	1	0	0	1	5
hsa-miR-708	ZNF275	1	1	0	1	0	0	1	0	0	1	5
hsa-miR-708	ZNF282	1	1	0	0	0	0	1	0	0	1	4
hsa-miR-708	ZNF319	1	1	0	1	0	0	1	0	0	1	5
hsa-miR-708	ZNF324	1	1	0	1	0	0	1	0	0	1	5
hsa-miR-708	ZNF324B	0	1	0	1	0	0	1	0	0	1	4
hsa-miR-708	ZNF35	1	1	0	1	0	0	1	0	0	1	5
hsa-miR-708	ZNF398	1	0	0	1	0	0	1	0	0	1	4
hsa-miR-708	ZNF41	1	0	0	1	0	0	1	0	0	1	4
hsa-miR-708	ZNF416	1	1	0	1	0	0	1	0	0	1	5
hsa-miR-708	ZNF425	1	1	0	1	0	0	1	0	0	0	4
hsa-miR-708	ZNF428	1	1	0	1	0	0	1	0	0	0	4
hsa-miR-708	ZNF436	1	0	0	1	0	0	1	0	0	1	4
hsa-miR-708	ZNF445	0	1	0	1	0	0	1	0	0	1	4
hsa-miR-708	ZNF462	1	1	0	1	0	0	1	0	0	0	4
hsa-miR-708	ZNF483	1	1	0	1	0	0	0	0	0	1	4
hsa-miR-708	ZNF488	1	1	0	1	0	0	1	0	0	1	5
hsa-miR-708	ZNF497	1	1	0	1	0	0	1	0	0	0	4
hsa-miR-708	ZNF507	1	1	0	1	0	0	1	0	0	1	5
hsa-miR-708	ZNF512	0	1	0	1	0	0	1	0	0	1	4
hsa-miR-708	ZNF518A	1	1	0	1	0	0	1	0	0	1	5
hsa-miR-708	ZNF557	1	1	0	1	0	0	0	0	0	1	4
hsa-miR-708	ZNF558	1	1	0	0	0	0	1	0	0	1	4
hsa-miR-708	ZNF559	1	1	0	1	0	0	1	0	0	0	4
hsa-miR-708	ZNF569	1	1	0	1	0	0	1	0	0	0	4
hsa-miR-708	ZNF584	1	1	0	1	0	0	1	0	0	0	4
hsa-miR-708	ZNF586	1	1	0	1	0	0	1	0	0	0	4
hsa-miR-708	ZNF589	1	1	0	1	0	0	1	0	0	1	5
hsa-miR-708	ZNF605	0	1	0	1	0	0	1	0	0	1	4
hsa-miR-708	ZNF614	1	1	0	1	0	0	1	0	0	1	5
hsa-miR-708	ZNF615	1	1	0	1	0	0	1	0	0	1	5
hsa-miR-708	ZNF618	0	1	0	1	0	0	1	0	0	1	4

hsa-miR-708	ZNF629	1	1	0	1	0	0	1	0	0	1	5
hsa-miR-708	ZNF652	0	1	0	1	0	0	1	0	0	1	4
hsa-miR-708	ZNF662	1	1	0	1	0	0	0	0	0	1	4
hsa-miR-708	ZNF664	0	1	0	1	0	0	1	0	0	1	4
hsa-miR-708	ZNF665	1	1	0	1	0	0	1	0	0	1	5
hsa-miR-708	ZNF74	1	1	0	0	0	0	1	0	0	1	4
hsa-miR-708	ZNF740	1	1	0	1	0	0	1	0	0	1	5
hsa-miR-708	ZNF776	1	1	0	1	0	0	1	0	0	1	5
hsa-miR-708	ZNF831	1	1	0	1	0	0	1	0	0	0	4
hsa-miR-708	ZNRF3	1	1	0	1	0	0	1	0	0	1	5
hsa-miR-708	ZSCAN22	1	1	0	1	0	0	1	0	0	0	4
hsa-miR-708	ZSCAN29	1	1	0	1	0	0	1	0	0	1	5
hsa-miR-708	ZSWIM5	1	1	0	1	0	0	1	0	0	1	5
hsa-miR-708	ZXDC	1	1	0	1	0	0	1	0	0	0	4

Table 9. GO enrichment analysis for the possible target genes of mir-708.

most significantly enriched GO term	count	*p*-Value	q-Value
GO:0006355 regulation of transcription, DNA-dependent	257	4.86736E-22	1.29796E-21
GO:0007050 cell cycle arrest	129	2.45324E-17	2.8037E-17
GO:0006350 transcription	148	2.49034E-16	2.21363E-16
GO:0030511 positive regulation of TGF beta receptor signaling pathway	102	2.64123E-06	3.38077E-07
GO:0008285 negative regulation of cell proliferation	86	7.90766E-12	4.86625E-12
GO:0007399 nervous system development	59	2.47789E-07	4.68473E-08
GO:0044419 interspecies interaction between organisms	63	4.52349E-16	3.61879E-16

(It is not all the consequence)
